# Efficacy and Key Materials of East Asian Herbal Medicine Combined with Conventional Medicine on Inflammatory Skin Lesion in Patients with *Psoriasis Vulgaris*: A Meta-Analysis, Integrated Data Mining, and Network Pharmacology

**DOI:** 10.3390/ph16081160

**Published:** 2023-08-15

**Authors:** Hee-Geun Jo, Hyehwa Kim, Eunhye Baek, Donghun Lee, Ji Hye Hwang

**Affiliations:** 1 Department of Herbal Pharmacology, College of Korean Medicine, Gachon University, 1342 Seongnamdae-ro, Sujeong-gu, Seongnam-si 13120, Gyeonggi-do, Republic of Korea; jho3366@hanmail.net; 2 Naturalis Inc. 6, Daewangpangyo-ro, Bundang-gu, Seongnam-si 13549, Gyeonggi-do, Republic of Korea; 3 KC Korean Medicine Hospital 12, Haeol 2-gil, Paju-si 10865, Gyeonggi-do, Republic of Korea; hh1635@hanmail.net; 4 RexSoft Inc., 1 Gwanak-ro, Gwanak-gu, Seoul 08826, Republic of Korea; 5 Department of Acupuncture and Moxibustion Medicine, College of Korean Medicine, Gachon University, 1342 Seongnamdae-ro, Sujeong-gu, Seongnam-si 13120, Gyeonggi-do, Republic of Korea

**Keywords:** East Asian herbal medicine, psoriasis, systematic review, chronic inflammation, network pharmacology, integrative medicine, social network analysis, association rule mining

## Abstract

Psoriasis is a chronic inflammatory disease that places a great burden on both individuals and society. The use of East Asian herbal medicine (EAHM) in combination with conventional medications is emerging as an effective strategy to control the complex immune-mediated inflammation of this disease from an integrative medicine (IM) perspective. The safety and efficacy of IM compared to conventional medicine (CM) were evaluated by collecting randomized controlled trial literature from ten multinational research databases. We then searched for important key materials based on integrated drug data mining. Network pharmacology analysis was performed to predict the mechanism of the anti-inflammatory effect. Data from 126 randomized clinical trials involving 11,139 patients were used. Compared with CM, IM using EAHM showed significant improvement in the Psoriasis Area Severity Index (PASI) 60 (RR: 1.4280; 95% CI: 1.3783–1.4794; *p* < 0.0001), PASI score (MD: −3.3544; 95% CI: −3.7608 to −2.9481; *p* < 0.0001), inflammatory skin lesion outcome, quality of life, serum inflammatory indicators, and safety index of psoriasis. Through integrated data mining of intervention data, we identified four herbs that were considered to be representative of the overall clinical effects of IM: *Rehmannia glutinosa* (Gaertn.) DC., *Isatis tinctoria* subsp. *athoa* (Boiss.) Papan., *Paeonia × suffruticosa* Andrews, and *Scrophularia ningpoensis* Hemsl. They were found to have mechanisms to inhibit pathological keratinocyte proliferation and immune-mediated inflammation, which are major pathologies of psoriasis, through multiple pharmacological actions on 19 gene targets and 8 pathways in network pharmacology analysis. However, the quality of the clinical trial design and pharmaceutical quality control data included in this study is still not optimal; therefore, more high-quality clinical and non-clinical studies are needed to firmly validate the information explored in this study. This study is informative in that it presents a focused hypothesis and methodology for the value and direction of such follow-up studies.

## 1. Introduction

Psoriasis is a chronic inflammatory disease of the skin that affects millions of people worldwide with a wide range of clinical symptoms [1]. A 2013 global epidemiological study found that adult prevalence ranged from 0.91 to 8.5%. The lifetime prevalence estimated by physicians was 6.3%, and a subsequent Danish cohort study supported this estimate [2,3]. Psoriasis is characterized by chronic inflammation resulting from the uncontrolled proliferation and differentiation of keratinocytes [4,5]. Inflammation of the skin on exposed areas of the body, such as the face and limbs, has a significant negative impact on the daily lives of the majority of people with psoriasis [6]. Many people with persistent psoriasis have a number of complications that might shorten their lifespans [7,8]. Recent studies have found a connection between psoriasis and other chronic diseases that may reduce life expectancy, such as psoriatic arthritis, hypertension, type 2 diabetes, dyslipidemia, myocardial infarction, and stroke [1,9,10,11]. In addition, a meta-analysis in 2022 revealed that the prevalence of psoriasis appears to increase the incidence of autoimmune thyroid disease [12]. According to these findings, psoriasis must be considered as a systemic disease that can increase the local skin condition of each patient’s burden on society [13]. Therefore, to reduce the severe impact of psoriasis on physical, social, and psychological well-being, it is necessary to find a way to effectively treat psoriasis.

Psoriasis can be clinically classified into four main subtypes: erythrodermic, guttate, pustular, and plaque psoriasis [1]. Skin-related lesions make up the majority of psoriasis lesions, with *psoriasis vulgaris* accounting for approximately 90% of all cases [1,4,7]. Erythematous, itchy plaques coated in silvery plaques are features of *psoriasis vulgaris* [4,14]. The scalp, elbows, face, and lumbosacral area are the most common places for plaques to appear as scaly skin lesions. They can vary widely in width and thickness [1,4]. When these plaques cover only 3–5% of the body surface in mild cases, topical treatment or phototherapy is often helpful [15]. However, systemic oral medications are required for moderate-to-severe plaque psoriasis [1,15]. Since psoriasis is a chronic disease that requires long-term medication, people with psoriasis often require lifelong therapy [16]. Consequently, all treatment plans must adhere to strict standards for patient safety. Although many conventional medicines (CMs) are available, there are still certain limitations to systemic therapy for psoriasis that need to be addressed. For example, acitretin is contraindicated in women of reproductive age due to its teratogenicity and because adverse events (AEs) such as dose-dependent alopecia and xerosis have been observed. Meanwhile, the long-used drug methotrexate has side effects including hepatotoxicity and bone marrow suppression that can lead to cirrhosis [4,17]. On the other hand, although methotrexate has been used for a long time, the possibility of inducing liver cirrhosis along with side effects such as hepatotoxicity and bone marrow suppression is still a concern [1,18,19].

Natural products have been considered as promising candidates for the treatment of various chronic diseases worldwide because they are safer than novel synthetic drugs even after prolonged administration and with high patient compliance [14,20,21,22]. Of these trends, the most active area in the discovery of promising materials related to psoriasis is East Asian herbal medicine (EAHM) [23,24,25,26,27,28,29]. The term “EAHM” refers to herbal therapies approved for use as medicines in a number of East Asian countries, including China, Taiwan, Korea, and Japan [23,24,25,26,27,30,31,32,33,34]. EAHM is significantly different from natural resources in other parts of the world as many similar medicinal plants are commonly listed in the pharmacopeia of East Asian countries [24,32,35]. At the same time, integrative medicine (IM) studies of chronic disease management are often conducted in countries with a history of medicinal herb use [36,37,38,39,40,41,42,43,44,45]. IM is a comprehensive strategy that uses both complementary and conventional therapies simultaneously. As a subset of IM, the combination of CMs and natural products has shown superior therapeutic efficacy and safety compared to standard therapies for a number of ailments, including COVID-19, cancer, stroke, chronic pruritus, and rheumatoid arthritis [36,40,46,47,48,49,50,51,52,53,54]. Meanwhile, a recent study using a bioinformatics approach reported that the effectiveness of widely used EAHM prescriptions in psoriasis is related to the suppression of oxidative stress and the alleviation of the resulting inflammatory pathology [55].

Considering several previous studies on this topic, it is reasonable to assume that IM may improve the psoriasis area and severity index (PASI) and clinical symptoms while reducing AEs associated with CMs in psoriasis patients [56,57,58,59,60]. However, several issues must be resolved before identifying candidate materials for optimal IM utilization and making robust clinical decisions. First, EAHM was administered in the form of a polyherbal prescription tailored to the clinical findings of each patient, and the maximization of the synergistic effect expressed in the appropriate combination of these herbs was estimated as the key mechanism of action [30,41,61,62,63]. Therefore, it is challenging to identify candidate materials for optimal combination pharmacotherapy and sufficient discussion cannot be performed based only on the existing studies. Moreover, despite the considerable amount of evidence gathered, a detailed analysis of the dosage route and composition of diverse materials is lacking. These are important factors that prevent coherent conclusions from being drawn. Therefore, more research is needed to inform whether IM with EAHMs is a useful intervention with a benefit in psoriasis, and specifically which EAHMs should be used.

In recent years, “reliable data” and the “integration of a wide range of analytical methodologies” have been suggested as prerequisites for natural product-based drug research [64]. The authors established the following research objectives based on their understanding and expertise from previous studies: (1) a systematic review of randomized controlled clinical trials (RCTs) will determine whether IM using EAHM is worthy of investigation for the treatment of inflammatory skin lesions in plaque psoriasis, and (2) further data analysis of herbal prescription data collected through this review will lead to hypotheses regarding promising candidates for the best IM for plaque psoriasis. Through the above research, the authors have attempted to open a discussion on a multifaceted analysis method that can overcome the complex variable problem of EAHM and produce useful information that can be used in useful follow-up studies.

## 2. Materials and Methods

### 2.1. Research Workflow through Integrated Methodology

**Figure 1:**
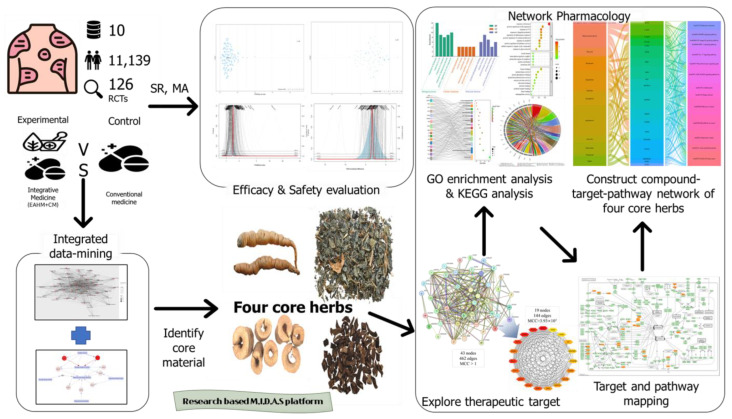
Study workflow of the multi-faceted analysis of IM for inflammatory skin lesion of psoriasis.

This study was carried out according to the following steps: (1) Clinical trial information was collected according to the systematic review methodology. At this stage, statistically valid evidence related to IM was obtained for efficacy and safety. Through a meta-analysis of the collected clinical trials, we first determined whether the IM approach using EAHM was a data pool worth searching for useful candidates. Once this condition was met, multiple data mining were performed as the second step, (2) deriving core materials through multiple data mining of drug information, was carried out. Through this, useful core materials predicted to exert the greatest weight in the pharmacology of EAHM theory were selected. Then, the appropriate dosage and duration of administration were investigated. (3) Prediction of the mechanism supporting the efficacy of the derived core material. The mechanism of action supporting the efficacy of the identified core herbs on inflammatory skin damage in psoriasis was predicted using systems biology methodology. Finally, based on the above steps, new IM information derived from clinical trial data was searched for crude drug usage patterns, doses, administration periods, compounds, targets, and pathways. This study was conducted as a process of building multidisciplinary-integrative-decision making-actual achievement-scientific creativity (M.I.D.A.S) research platform. The workflow of the methodology described above is summarized in Figure 1.

### 2.2. Data Sources and Search Strategy

The systematic exploration of clinical trial data for this study was conducted in accordance with the Preferred Reporting Items for Systematic Reviews and Meta-Analysis (PRISMA) 2020 statement [65], and the protocol was pre-registered in PROSPERO (registration number: CRD 42022296852, available from https://www.crd.york.ac.uk/PROSPERO/display_record.php?RecordID=296852). In addition, the protocol of this study has been formally published [66].

RCTs that evaluated the efficacy and safety of IM for *psoriasis vulgaris* were searched in the following 10 electronic databases from their inception until 29 July 2021: three English databases (PubMed, Cochrane Library, and Embase), four Korean databases (Korean Studies Information Service System, Research Information Service System, Oriental Medicine Advanced Searching Integrated System, and Korea Citation Index), two Chinese databases (Chinese National Knowledge Infrastructure Database, Wanfang data), and one Japanese database (Citation Information by National Institute of Informatics). The overall literature search procedure was carried out independently by two researchers (HGJ and HK). Detailed search strategies are presented in Supplementary Table S1.

### 2.3. Study Selection

#### 2.3.1. Type of Studies

Only RCTs evaluating the efficacy and safety of IM for inflammatory skin lesion in plaque psoriasis were included. There were no restrictions on the language or publication time. Studies that meet the following criteria were excluded: (a) studies that are not RCTs or quasi RCTs; (b) studies not related to plaque psoriasis or related diseases; (c) primary intervention not related to IM; (d) no oral administration of medications; (e) not a clinical trial; (f) case reports or reviews; (g) studies not published in scientific peer-reviewed journals, including postgraduate theses or dissertations; and (h) studies in which the experimental intervention was not based on an IM approach, such as EAHM monotherapy.

#### 2.3.2. Type of Participants

There were no restrictions on age, gender, or race, and studies were only eligible for inclusion if they were conducted in patients with a diagnosis of *psoriasis vulgaris*. Only studies that provided official or validated diagnostic criteria were included. Studies that included patients with additional psoriasis subtypes, such as psoriatic arthritis, guttate psoriasis, palmoplantar pulposus, and erythrodermic psoriasis, were excluded from the review because the focus of the review was on plaque psoriasis.

#### 2.3.3. Type of Interventions

RCTs evaluating the active intervention of IM (EAHM combined with CMs) in the treatment group versus CMs alone in the control group were included. For inflammatory skin lesions in psoriasis, all dosage forms of IM intervention were considered, including decoction, granules, capsules, and tablets. There were no restrictions on dosage or duration of therapy, but oral ingestion was the only acceptable method of administration. Trials that included non-drug treatment, acupuncture, massage, or other complementary therapies only in the experimental group and not in the control group were excluded. Studies that could not confirm the composition of specific herbal ingredients included in the EAHM formula were excluded.

#### 2.3.4. Type of Outcome Measures

The primary endpoint was the response rate of patients with a PASI improvement of 60% (PASI 60). The primary outcome was also the absolute difference in PASI scores between the groups. In the first set of secondary outcomes, an improved PASI of 70% (PASI 70), recurrence rate, dermatological quality of life index (DLQI), and visual analog scale (VAS) were adopted to measure the clinical response of inflammatory skin lesions in patients with psoriasis. As the second group of secondary outcomes, TNF-α, IL-8, IL-17, IL-22, IL-23, and IFN-γ were selected to evaluate changes in inflammation-related biomarkers that support improvement of inflammatory skin lesions. The safety of IM was assessed using the incidence rates of adverse events (AEs) in each group as the third set of secondary outcomes.

### 2.4. Data Extraction and Management

Two researchers (HGJ and HK) independently retrieved the titles and abstracts of potentially eligible articles using the search approach described above. The inclusion and exclusion criteria were then used to guide a full-text review. Two reviewers (HGJ and HK) independently retrieved data from the included studies. Two reviewers independently extracted the following data for the selected trials:

1. Publication information (title, first author, year of publication, and funding source).
2. Study characteristics (trial design, randomization method, sample size, treatment duration, and morbidity period).
3. Participants (age, sex, diagnostic criteria, and number of participants in each group).
4. Intervention (experimental intervention, comparator, ingredients, and detailed information on intervention frequency of medication, dosage, mode of delivery, and course of treatment).
5. Outcomes (primary and secondary outcomes, measurement point, blinding of outcome assessment, and AEs).

All disagreements were resolved through discussions with the researchers and the other author (DL).

### 2.5. Methodological Quality Assessment

The methodological quality of each included study was independently evaluated by two investigators (HGJ and HK) according to the revised version of risk of bias in randomized trials (RoB 2.0) [67]. Five areas of bias are addressed by RoB 2.0: bias arising from the randomization process, bias deviating from the intended intervention, bias due to the omission of outcome data, and bias in the selection of reported outcomes. There were three categories used to rate the study’s methodological quality: “high risk of bias,” “low risk of bias,” and “some concerns.”. Disagreements between investigators were resolved by consensus with the assistance of another author (DL).

### 2.6. Quality of Evidence according to Outcome Measures

The overall quality of evidence for each outcome was evaluated using the Grading of Recommendations Assessment, Development, and Evaluation (GRADE) pro framework [68]. GRADE evaluates the overall quality of evidence at four levels: very low, low, moderate, and high. The level of evidence is lowered according to factors such as the risk of bias, inconsistency, indirectness, imprecision, and publication bias.

### 2.7. Statistical Analysis

#### 2.7.1. Data Synthesis of Clinical Outcomes

In the meta-analysis of the included data from clinical trials, the effect size and 95% confidence intervals (CI) were estimated using only the random-effects model. When either the χ2 test was <0.10 or I^2^ was ≥50%, heterogeneity was considered statistically significant. Statistical synthesis of individual research results was performed using R software (version 4.1.2) and R studio program (Version 1.4.1106, Integrated Development for R. Rstudio, PBC, Boston, MA, USA) using the default settings of the “meta” and “metafor” packages [69].

Trials were categorized according to the type of intervention and comparator. The relative risk (RR) and 95% confidence interval (CI) were calculated for the PASI60, PASI70, and recurrence rates. The mean difference (MD) and 95% confidence interval (CI) were estimated for the PASI, DLQI, and VAS scores. For TNF-α, IL-8, IL-17, IL-22, IL-23, and IFN-γ, the standardized mean difference (SMD) and 95% confidence intervals (CIs) were computed to integrate the results of the indicators in different units related to the same measurement object. Due to the need to estimate a causal relationship, the odds ratio (OR) was used to quantify incidence rates of adverse events.

In addition to the forest plot, a drapery plot was used to better represent the effect size in this study, rather than relying solely on the primary outcome synthesis data with *p* < 0.05 as the level of significance [70]. Meanwhile, this study combined much more data than the previous meta-analysis. Forest plots cannot effectively represent such enormous amounts of data. After considering the results of more than 50 studies, an orchard plot was chosen to display the data instead of a forest plot [71].

When heterogeneity was identified in the primary outcomes of the meta-analysis, further analyses were performed to determine the explanation. First, a leave-one-out sensitivity analysis was performed to determine whether the included data were affected by outliers. If no outliers were identified, a subgroup analysis was performed after performing meta-regression analysis for the following seven pre-specified variables: (i) comparator drug, (ii) treatment duration, (iii) source of investigational medicine, (iv) formulation type, (v) sample size, (vi) overall risk of bias, and (vii) randomization method that caused a significant difference in results. A contour-enhanced funnel plot was used in the meta-analysis for the primary outcome of more than ten trials to distinguish publication bias [72]. Egger’s and Begg’s tests were performed to confirm the existence of publication bias for the asymmetry of the visually observed funnel plot [73,74].

#### 2.7.2. Deriving Core Herbs Based on Data Mining Approach

In order to maximize the synergistic effect, the principle of use for EAHM differs from that of other natural medicines in that it uses a polyherbal formulation. “Gun-Shin-Jwa-Sa”, also known as Sovereign-Minister-Assistant-Courier in the official nomenclature of the WHO, is the formulation theory for the ideal combination of EAHM [30]. The major pharmacological medication among them is referred to as the sovereign or monarch drug. EAHM prescriptions can be taken as a single dose due to the effects of this sovereign drug, while the other drugs both reduce the toxicity of the entire prescription and enhance the effects of the sovereign drugs [24,61,75]. Therefore, when data mining is used to identify core herbs that are likely to be sovereign drugs, their pharmacological effects can be considered representative of the overall clinical efficacy of EAHM. Based on this theoretical background, this review assumed that the improvement in psoriasis observed in the overall IM clinical data could be explained by the pharmacological effects of the core herbs, which were derived based on the following methodology.

First, the type of EAHM commonly used in more than 10% of the studies, the dose, and the duration of use of commonly prescribed EAHM were examined using a descriptive statistical approach.

Second, using social network analysis, we selected the EAHM component of the IM prescription construct, which plays a central role in the relationship between the different drugs. The social network analysis used in this study was divided into two parts. The network was assumed to be undirected and the degree distribution of the connections between the common EAHM substances utilized in each IM prescription was observed. The average degree of connection in this situation may be represented as follows because an undirected network is assumed:

$$A=\sum_{K=1}^{n}kP\left(k\right)=\frac{2E}{n}$$

where *n* is the number of nodes and *E* is the number of links.

By evaluating the power of specific materials on the association between frequently prescribed EAHMs, centrality was used to discover EAHM with relatively greater influence. The eigenvector centrality scale was used to analyze the association between each of the concurrently administered herbs.

$$Ci=\frac{1}{\lambda }\sum_{j\in N(i)}A_{ij}C_{j}$$

where N(*i*) represents the collection of herbs that are close to material *i* and λ is the eigenvalue of material *i*, a constant determined by the algorithm. If materials *i* and *j* are connected in the n × n-direction adjacency matrix A, A*ij* becomes “1”; otherwise, it becomes “0”. Herb *i* and its neighbors constitute herb *j*, which is the eigenvector centrality value of C*j*. Centrality measurements were performed on materials showing a frequency of use in more than 5% of the included trials.

Third, association rule mining was performed to discover a meaningful combination pattern among all materials included in the IM prescription [76]. The frequent combination pattern itself may be a relevant herb unit representing clinical tacit knowledge, as EAHM is administered in a combination to optimize synergy. Support, confidence, and lift were the primary measures used in association rule mining. The support of the itemset is the proportion of transactions in the dataset that contain it. Itemsets whose support exceeds a user-defined minimum support level are considered frequent. A rule X ⇒ Y’s confidence is expressed as conf (X ⇒ Y) = support(X∪Y)/support(X). Assuming that the transactions in question also contain the antecedent, this may be explained as an estimate of the probability P (Y|X) or the likelihood of finding the rule’s consequent in those transactions. Another index called the lift can be represented by the following equation:

$$lift\left(X\Rightarrow Y\right)=\frac{supp(X\cup Y)}{supp\left(X\right)supp(Y)}$$

The lift can be explained as the deviation of all rules supports from the support anticipated under independence, given the support of the rule on both sides. Stronger associations are indicated by higher lift values.

Finally, through the above three steps, EAHM that simultaneously satisfies the three conditions of “frequent use in clinical practice”, “central position within individual prescriptions”, and “strong association with other drugs” was selected as the core herbs. The representative values of the dosing period and dose of these drugs are presented together by calculating the inter-quartile range.

#### 2.7.3. Prediction of Anti-Inflammatory Mechanisms Based on Network Pharmacology

A network pharmacology analysis was performed to explore the anti-inflammatory mechanism of core herbs derived through the above data mining. The chemical ingredients in herbs were obtained from the TCMSP (Traditional Chinese medicine systems pharmacology database and analysis platform, https://therbsp-e.com/), TCMID (Traditional Chinese Medicine integrative database for herb molecular mechanism analysis, http://bidd.group/TCMID/), HERB (a high-throughput experiment-and reference-guided database of traditional Chinese medicine, http://herb.ac.cn/), and ETCM (an encyclopedia of traditional Chinese medicine, http://www.tcmip.cn/ETCM/) databases [77,78,79,80]. In this study, compounds with an oral bioavailability (OB) of 20% and a drug-like (DL) index of 0.1 were first screened according to the information provided by the TCMSP DB, and then a potential compound was finally selected by performing a second round of absorption, distribution, metabolism, and excretion (ADME) prediction using the SwissADME platform (http://www.swissadme.ch/). In the SwissADME platform, compounds were selected if at least three of the five ADME rules of Lipsinski, Ghose, Veber, Egan, and Muegge were evaluated as “yes” [81]. Potential target genes of selected active compounds were predicted by the SwissTargetPrediction platform (http://www.swisstargetprediction.ch) in the “*Homo sapiens*” setting and selected by the “Probability ≥ 0.1” criterion [82]. Using the “*Homo sapiens*” species filter in the Uniprot database (http://www.uniprot.org), the target information for active substances was standardized. Data on psoriasis-related target genes were retrieved from the GeneCards database (http://www.genecards.org) with “*psoriasis vulgaris*” as the keyword. For targets in GeneCards, only those with a score ≥ 10 were screened [83].

Using the “Bioinformatics & Evolutionary Genomics site” (https://bioinformatics.psb.ugent.be/webtools/Venn/), Venn diagrams for common targets between core herbs and psoriasis were created. To graphically represent the intricate interactions among chemicals and targets, a network comprising the elements of the core herbs and psoriasis targets was built using Cytoscape (version 3.10.0; https://cytoscape.org/). The protein classification “*Homo sapiens*” and the STRING protein analysis platform (12.0 beta; https://version-12-0.string-db.org/) were used to import the interaction gene targets of the core herbs and psoriasis [84]. The minimum required score to define a protein–protein interaction was set to “medium confidence (confidence score ≥ 0.4)”. We constructed the PPI network, removed unnecessary protein nodes, and then loaded the data into Cytoscape using the Cytohubba plugin for topological analysis of the PPI network [85,86]. The selection of hub gene targets was based on the score calculated by cytoHubba’s Maximum Clique Centrality (MCC) algorithm being greater than three fold of the median. The main method for characterizing the function of gene targets, including biological processes, cellular components, and molecular functions, was gene ontology (GO) functional analysis. The shared targets of the core herbs and psoriasis in signaling pathways were discovered using the Kyoto Encyclopedia of Genes and Genomes (KEGG) enrichment analysis. More than 40 gene function annotation datasets are included in the web tool for gene enrichment analysis Metascape (https://metascape.org/). For GO and KEGG analysis, the hub targets were uploaded to the Metascape platform [87]. The *p* < 0.05 data selection threshold was selected. The KEGG mapper (https://www.genome.jp/kegg/mapper/) was used to explore their underlying molecular mechanisms [88].

## 3. Results

### 3.1. Study Identification

**Figure 2:**
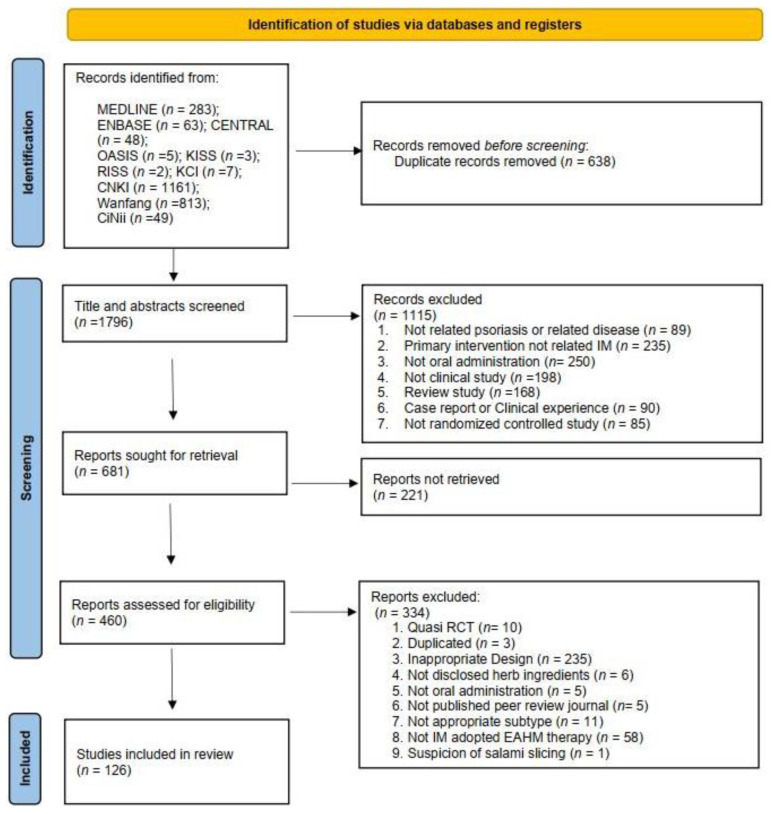
PRISMA 2020 flow diagram.

By implementing the search strategy, the electronic search of the 10 databases identified 2434 potentially relevant articles. After removing 638 duplicate records, a total of 1796 records were collected. After screening for titles and abstracts, 1115 articles that met at least one of the exclusion criteria were excluded. The full-text assessment was performed on the remaining 460 studies, and 334 articles were excluded for the reasons listed in Figure 2. Finally, 126 eligible studies were included in this meta-analysis [89,90,91,92,93,94,95,96,97,98,99,100,101,102,103,104,105,106,107,108,109,110,111,112,113,114,115,116,117,118,119,120,121,122,123,124,125,126,127,128,129,130,131,132,133,134,135,136,137,138,139,140,141,142,143,144,145,146,147,148,149,150,151,152,153,154,155,156,157,158,159,160,161,162,163,164,165,166,167,168,169,170,171,172,173,174,175,176,177,178,179,180,181,182,183,184,185,186,187,188,189,190,191,192,193,194,195,196,197,198,199,200,201,202,203,204,205,206,207,208,209,210,211,212,213,214]. The screening process is summarized in the PRISMA 2020 flow diagram (Figure 2).

### 3.2. Study Characteristics

The basic characteristics of the 126 included studies are summarized in Table 1. In general, the sample size of the included studies ranged from 34 to 260, and 11,139 participants were divided into an experimental (*n* = 5624) and a control group (*n* = 5515). Acitretin (78 trials), topical corticosteroids (28 trials), immunosuppressants (8 trials), methotrexate (4 trials), topical urea (3 trials), eritretin (1 trial), topical retinoid (1 trial), topical pyrithione zinc (1 trial), topical boric acid (1 trial), and an ascorbate and pyridoxine combination (1 trial) were administered to the control group in all the included studies. PASI 60 was measured as the primary outcome in 96 studies. The PASI score, a primary outcome measure of the extent of inflammatory skin lesions, was used in 69 studies. In terms of inflammatory skin lesion-related secondary outcomes, PASI 70 was the score measured in 13 studies. Another secondary endpoint, the recurrence rate, was reported in 12 studies. A change in the DLQI was observed in ten studies. The VAS scores were observed in three studies. As a secondary outcome to measure laboratory findings related to inflammatory skin lesions, TNF-α, IL-8, IL-17, IL-22, IL-23, and IFN-γ were reported in twelve, eight, ten, four, five, and six studies, respectively. As a secondary outcome to evaluate the safety of IM versus CM, 75 trials reported information that could be used to compare the incidence rates of AEs in the experimental and control groups. All the included studies reported the treatment duration. It ranged from 2 to 16 weeks, with 31 studies adopting a treatment period of ≥12 weeks.

### 3.3. Risk of Bias

**Figure 3:**
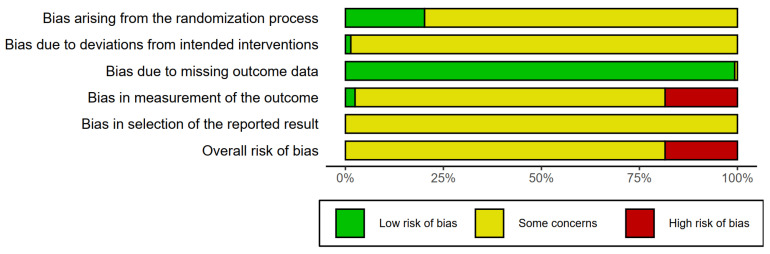
Risk of bias 2.0 summary: authors’ judgements for each risk of bias domain across all included trials.

The methodological quality of the 126 included studies is summarized in Figure 3 and Table 2. The risk of bias in the studies was assessed using the Rob 2.0 tool [67]. In 101 studies, the overall risk of bias, in one or more domains, was evaluated to be of “some concern”. In all other studies, the overall risk of bias was evaluated to be “high”. Twenty-six studies reported detailed information on the randomization process, and only five studies explicitly revealed that a single-blind design was adopted [129,152,161,163,202]. In the remaining studies, it was difficult to obtain specific information other than the fact that randomization was performed, and the adoption of a blinded design was uncertain. In addition, a potential common factor that could affect the risk of bias was the absence of a preregistered protocol that could prevent selective outcome reporting.

### 3.4. Primary Outcomes

#### 3.4.1. PASI 60

**Figure 4:**
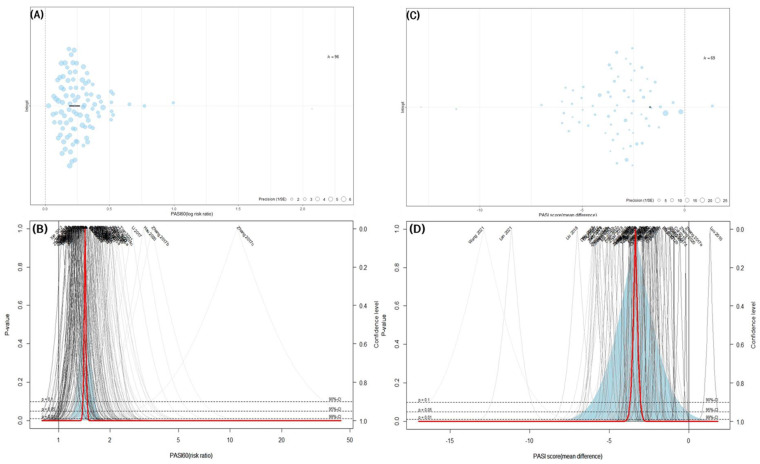
(**A**) Orchard plot of the trials that compared IM with CM for the PASI 60; (**B**) Drapery plot of the trials that compared IM with CM for the PASI 60; (**C**) Orchard plot of the trials that compared IM with CM for the PASI score; (**D**) Drapery plot of the trials that compared IM with CM for the PASI score. The red line in the drapery plot is the P-value curve of the pooled estimates; the light blue region in the drapery plot is the prediction region.

A meta-analysis was performed on 96 studies that reported PASI 60. The combined effect of IM on the response rate was significantly better than that of the CM control (96 trials, *n* = 8367; RR: 1.4280; 95% CI: 1.3783–1.4794; *p* < 0.0001, heterogeneity chi-square = 118.79, df = 95, I^2^ = 20.0%; *p* = 0.0498; Figure 4A,B).

#### 3.4.2. PASI Score

In the 69 studies comparing the effect of IM with that of the CM control, IM exhibited a significantly improved PASI score compared to the CM control (69 trials, *n* = 5801; MD: −3.3544; 95% CI: −3.7608 to −2.9481; *p* < 0.0001, heterogeneity chi-square = 3712.58, df = 68, I^2^ = 98.2%; *p* < 0.0001; Figure 4C,D).

### 3.5. Secondary Outcomes Group 1: Assessment of Patient Outcome Related to Inflammatory Skin Lesion

**Figure 5:**
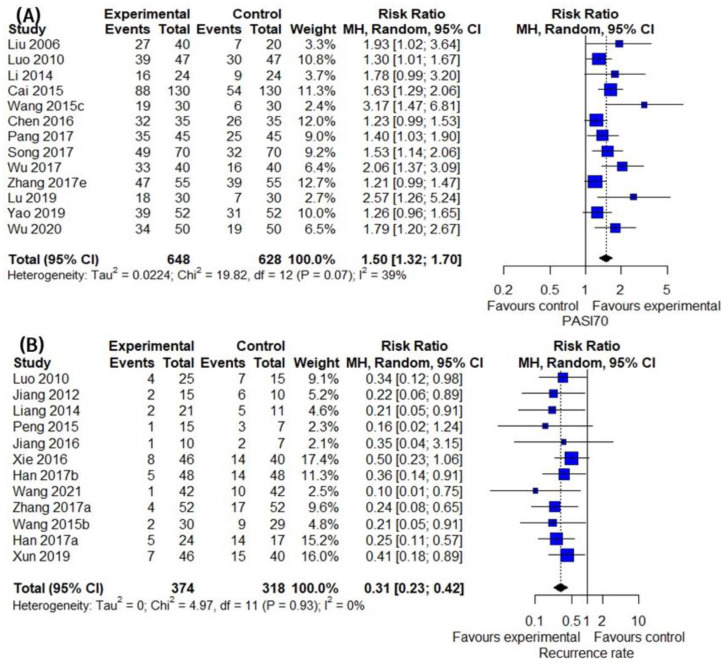

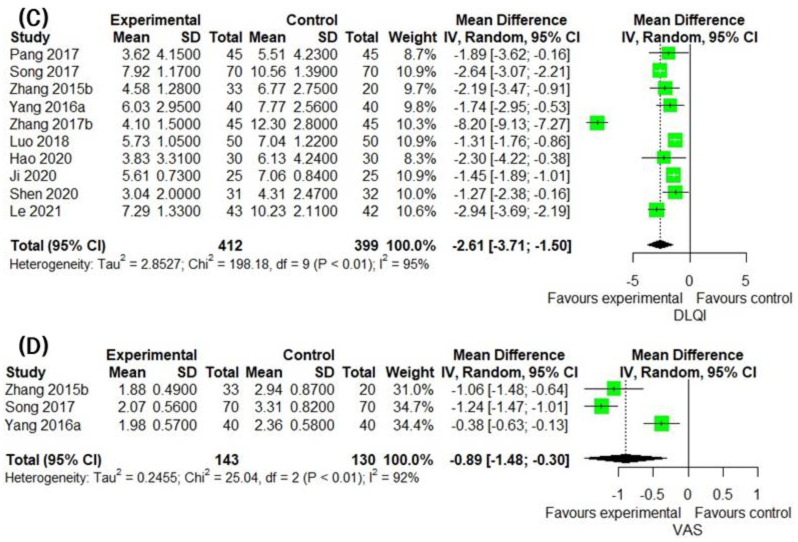
Forest plot of the trials that compared IM with CM for (**A**) PASI 70; (**B**) Recurrence rate; (**C**) DLQI; (**D**) VAS.

In the 13 studies comparing the effect of IM with that of the CM control, IM exhibited a significantly improved PASI 70 compared to the CM control (13 trials, *n* = 1276; RR: 1.4994; 95% CI: 1.3208–1.7021; *p* < 0.0001; heterogeneity chi-square = 19.82, df = 12, I^2^ = 39.5%; *p* = 0.0705; Figure 5A). Twelve studies compared IM with CM controls regarding the recurrence rate. The combined effect of IM on the recurrence rate was significantly lower than that of the CM control (12 trials, *n* = 692; RR: 0.3095; 95% CI: 0.2256–0.4245; *p* < 0.0001, heterogeneity chi-square = 4.97, df = 11, I^2^ = 0%; *p* = 0.9325; Figure 5B). In the ten studies comparing the effect of IM with the CM control, IM significantly improved the DLQI compared to the CM control (10 trials, *n* = 811; MD: −2.6072; 95% CI: −3.7100 to −1.5043; *p* < 0.0001, heterogeneity chi-square = 198.18, df = 9, I^2^ = 95.5%; *p* < 0.0001; Figure 5C). VAS scores were reported in three studies. The reduction in VAS score was significantly greater in the IM group than in the CM control (three trials, *n* = 273; MD: −0.8888; 95% CI: −1.4769 to −0.3008; *p* = 0.0031, heterogeneity chi-square = 25.04, df = 2, I^2^ = 92.0%; *p* < 0.0001; Figure 5D).

### 3.6. Secondary Outcomes Group 2: Assessment of Laboratory Biomarkers Related to Inflammatory Skin Lesion

**Figure 6:**
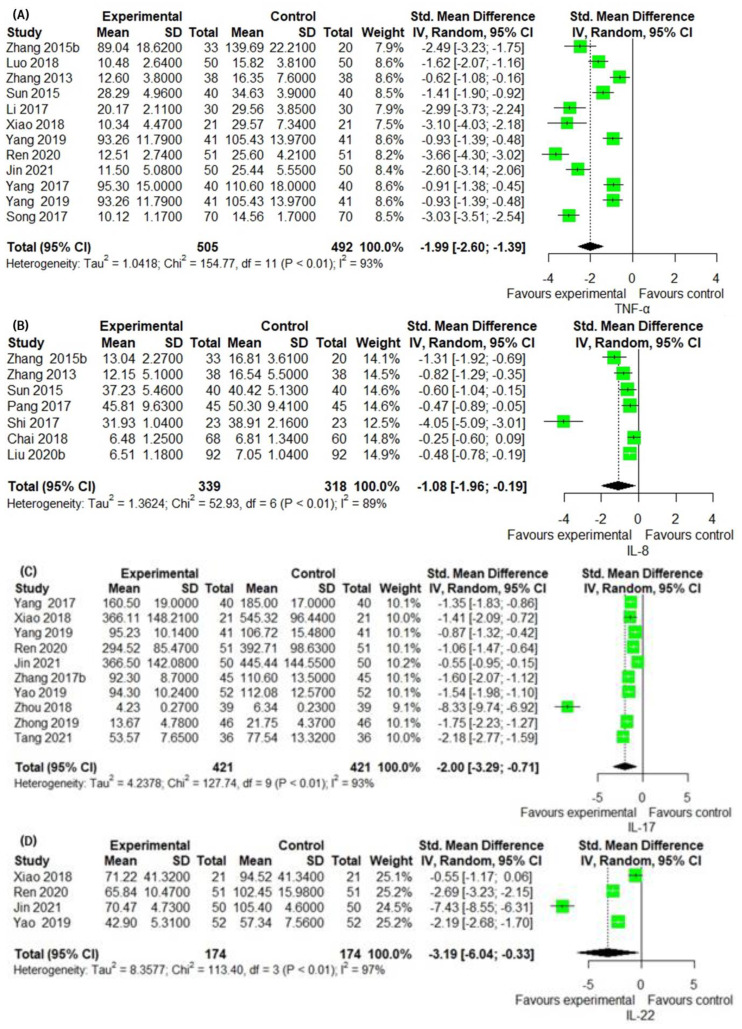

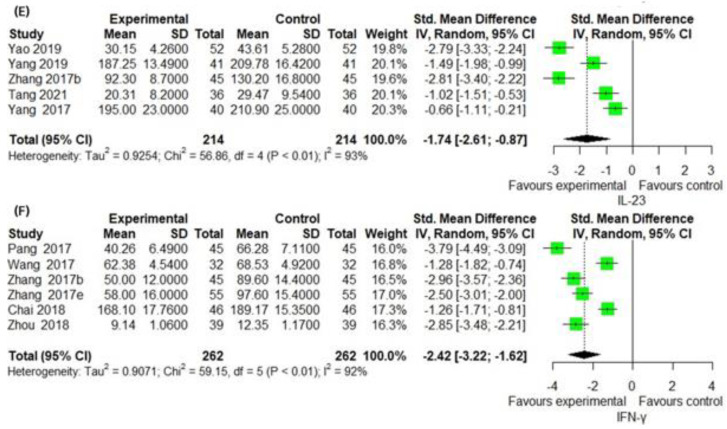
Forest plot of the trials that compared IM with CM for (**A**) TNF-α; (**B**) IL-8; (**C**) IL-17; (**D**) IL-22; (**E**) IL-23; (**F**) IFN-γ.

The TNF-α levels were measured in 12 studies. TNF-α levels in the IM group were significantly lower than in the CM control (12 trials, *n* = 997; SMD: −1.9948; 95% CI: −2.5964–−1.3932; *p* < 0.0001, heterogeneity chi-square = 154.77, df = 11, I^2^ = 92.9%; *p* = 0.3361; Figure 6A). IM remarkably decreased IL-8 levels compared to the CM control (seven trials, *n* = 657; SMD: −1.0752; 95% CI: −1.9647 to −0.1856; *p* = 0.0178, heterogeneity chi-square = 52.93, df = 6, I^2^ = 92.9%; *p* < 0.0001; Figure 6B). In the ten studies comparing the effect of IM with that of the CM control, IM significantly decreased IL-17 levels compared to the CM control (10 trials, *n* = 842; SMD: −2.0009; 95% CI: −3.2927 to −0.7091; *p* = 0.0024, heterogeneity chi-square = 127.74, df = 9, I^2^ = 93.0%; *p* < 0.0001; Figure 6C). The effect of IM on IL-22 was reported in four studies. A significant improvement in IL-22 by IM was identified by the CM control (four trials, *n* = 348; SMD: −3.1874; 95% CI: −6.0441 to −0.3307; *p* = 0.0288, heterogeneity chi-square = 113.40, df = 3, I^2^ = 97.4%; *p* < 0.0001; Figure 6D). IL-23 was reported in five studies. The reduction in IL-23 was significantly greater in the IM group than in the CM control (five trials, *n* = 428; SMD: −1.7398; 95% CI: −2.6139 to −0.8657; *p* < 0.0001, heterogeneity chi-square = 56.86, df = 4, I^2^ = 93.0%; *p* < 0.0001; Figure 6E). IFN-γ levels were reported in six studies. Compared with CM controls, IM significantly decreased IFN-γ levels (six trials, *n* = 524; SMD: −2.4211; 95% CI: −3.2187 to −1.6235; *p* < 0.0001, heterogeneity chi-square = 59.15, df = 5, I^2^ = 91.5%; *p* < 0.0001; Figure 6F).

### 3.7. Secondary Outcomes Group 3: Safety Assessment

A total of 79 trials (79/126, 62.7%) reported detailed information on AEs in the IM and CM control groups. In four trials, detailed information for each group of AEs was not reported [95,106,126,162]. Therefore, a safety assessment was performed by extracting AEs data from 75 trials. The individual symptoms of the reported AEs for each trial are presented in Table 1. AEs that occurred during the treatment of psoriasis could be classified into the following four categories: drug-induced liver injury, including findings of impaired hepatic function and elevated blood aspartate transaminase levels; cutaneous symptoms suggesting worsening of skin findings, such as xerostomia, xeroma, and xeroderma; alimentary symptoms, including abdominal discomfort and diarrhea after taking medication; and metabolic disorders reported primarily in the form of hyperlipidemia. Hence, the incidence rates were compared between the groups by dividing the reported AEs into the four categories mentioned above and one category that included other symptoms that could not be classified into these categories in this review.

**Figure 7:**
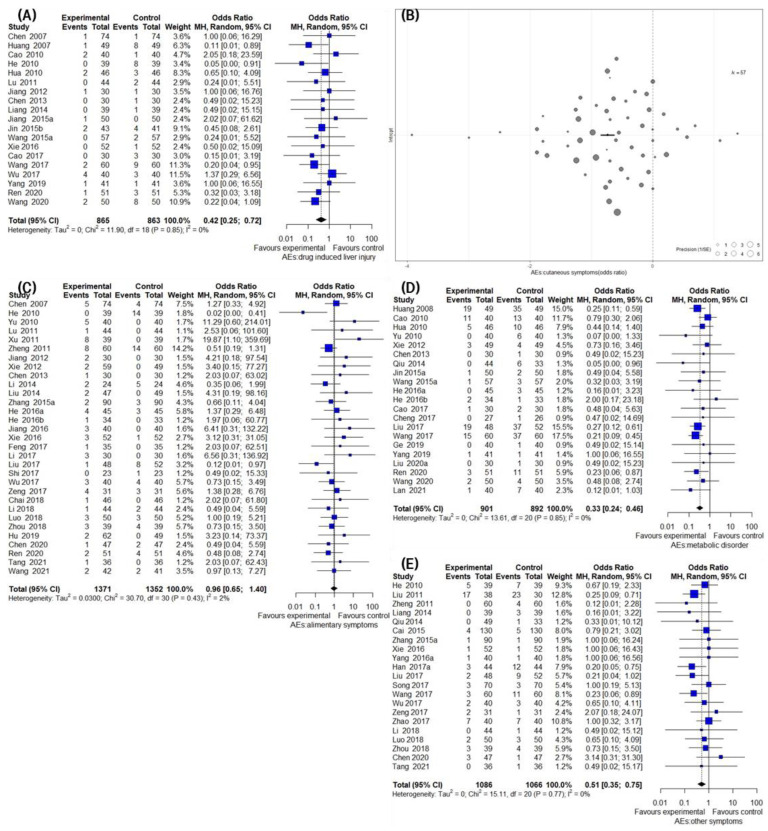
Forest plot of the incidence rates of reported adverse events: (**A**) drug induced liver injury; (**B**) cutaneous symptoms; (**C**) alimentary symptoms; (**D**) metabolic disorder; (**E**) other symptoms.

A meta-analysis of 19 studies showed that IM significantly reduced the incidence of drug-induced liver injury compared to the CM control (19 trials, n = 1728; OR: 0.4219; 95% CI: 0.2482–0.7170; *p* = 0.0014, heterogeneity chi-square =11.90, df = 18, I^2^ = 0%; *p* = 0.8523; Figure 7A). The pooled results of 31 studies comparing IM and CM controls suggested that the incidence of AEs was significantly reduced by IM administration. A total of 57 trials evaluated the effect of IM on reducing the incidence rate of cutaneous AEs compared to the CM control. The meta-analysis revealed a significant reducing effect of IM on AEs (57 trials, *n* = 2723; OR: 0.3578; 95% CI: 0.2687 to 0.4763; *p* < 0.0001, heterogeneity chi-square = 205.79, df = 56, I^2^ = 72.8%; *p* < 0.0001; Figure 7B). The incidence rate of alimentary AEs in IM was similar to that of the CM controls, and there was no statistically significant difference between the two groups, according to a meta-analysis (31 trials, *n* =2723; OR: 0.9552; 95% CI: 0.6516 to 1.4002; *p* = 0.8143, heterogeneity chi-square = 30.70, df = 30, I^2^ = 2.3%; *p* = 0.4305; Figure 7C). In contrast, the pooled results of 21 studies comparing IM and the CM control suggested that the incidence of metabolic AEs was significantly reduced by IM (21 trials, *n* = 1793; OR: 0.3288; 95% CI: 0.2369–0.4562; *p* < 0.0001, heterogeneity chi-square = 13.61, df = 20, I^2^ = 0%; *p* = 0.8497; Figure 7D). In the 21 studies comparing the effect of IM with the CM control, IM significantly decreased the incidence rate compared to the CM control (21 trials, *n* = 2152; OR: 0.5098; 95% CI: 0.3488–0.7450; *p* = 0.0005, heterogeneity chi-square = 15.11, df = 20, I^2^ = 0%; *p* = 0.7701; Figure 7E).

### 3.8. Assessing Heterogeneity

#### 3.8.1. Sensitivity Analysis

**Figure 8:**
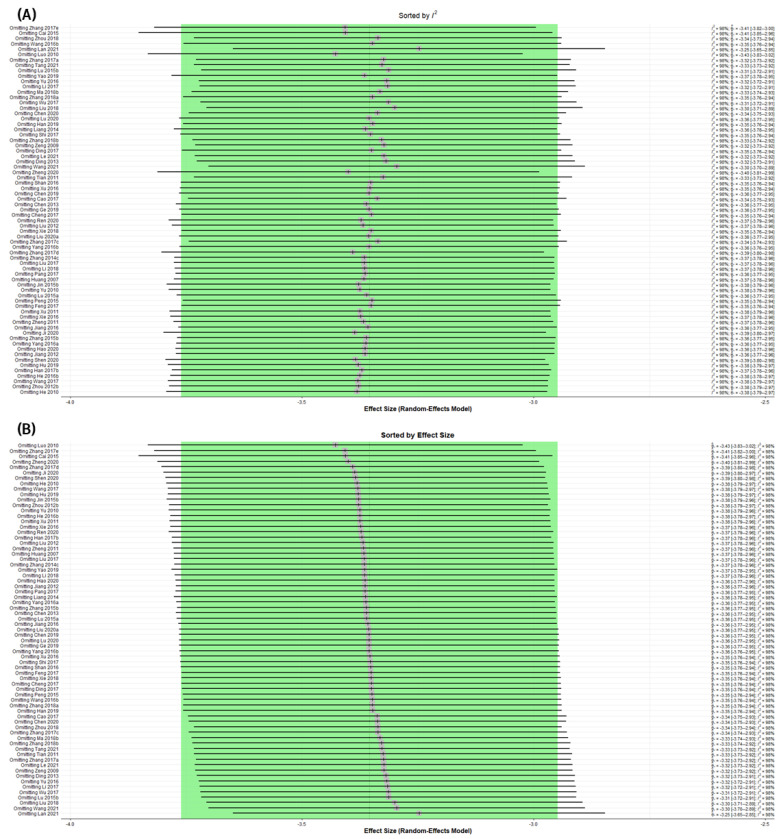
Forest plot of the sensitivity analysis ordered by heterogeneity for (**A**) PASI 60 and (**B**) PASI score.

Considerable heterogeneity was found in the synthesis of the trial data for PASI scores between the two primary outcomes of this study. In this respect, trials estimated to be potential outliers were also observed in the drapery plot. To ascertain whether particular research matching these outliers was the cause of heterogeneity, a sensitivity analysis using the leave-one-out method was performed. Sensitivity analysis revealed that no single study’s omission considerably affected the change in heterogeneity (Figure 8A,B).

#### 3.8.2. Meta-Regression and Subgroup Analysis

**Figure 9:**
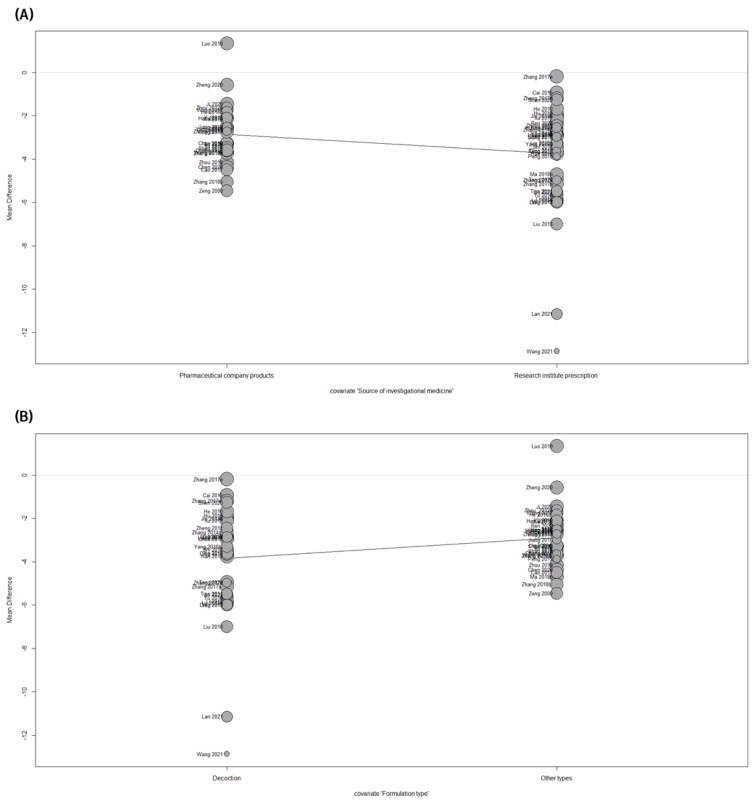
Bubble plot of the meta-regression analysis for (**A**) source of investigational medicine and (**B**) formulation type.

Sensitivity analysis demonstrated that specific individual studies corresponding to outliers did not affect heterogeneity. Consequently, a meta-regression analysis was carried out on moderators predicted to have an impact on the results to identify other possible sources of heterogeneity. The following moderators were examined in this study: (i) comparator drug, (ii) treatment duration, (iii) source of investigational medicine, (iv) formulation type, (v) sample size, (vi) overall risk of bias, and (vii) randomization method. Meta-regression analysis revealed that the variables “source of investigational medicine” (*p* = 0.0240) and “formulation type” (*p* = 0.0145) had a statistically noticeable influence on the pooled results (Figure 9A,B). However, the results of the meta-analysis were not significantly influenced by the comparator drug (*p* = 0.6215, Supplementary Figure S1A), treatment duration (*p* = 0.5563, Supplementary Figure S1B), sample size (*p* = 0.6098, Supplementary Figure S1C), overall risk of bias (*p* = 0.5615, Supplementary Figure S1D), or randomization method (*p* = 0.9857, Supplementary Figure S1E). Based on the meta-regression results, a subgroup analysis was performed on the source of investigational medicine and formulation type. However, subgroup analysis showed that neither moderator had a significant influence on the magnitude of the effect or degree of heterogeneity (Table 3).

### 3.9. Assessing Publication Bias

**Figure 10:**
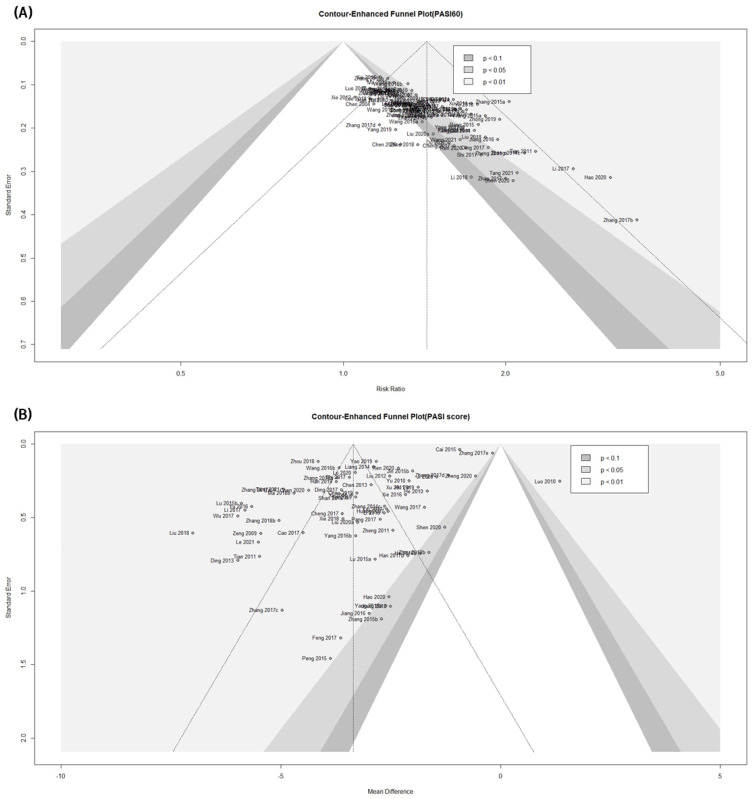
Contour-enhanced funnel plot of (**A**) PASI 60 and (**B**) PASI scores.

A contour-enhanced funnel plot and Egger’s and Begg’s tests were used to assess the potential publication bias of the primary outcomes in this meta-analysis. Asymmetric shapes were observed in the contour-enhanced funnel plot for PASI 60, suggesting potential bias (Figure 10A). The publication bias was statistically significant in the Egger’s and Begg’s tests for PASI 60 (Egger’s test: t = 10.67, df = 94, *p* < 0.0001; Begg’s test: z = 7.48, *p* < 0.0001). Unlike PASI 60, a distinct asymmetric shape suggestive of publication bias was not observed in the contour-enhanced funnel plot for the PASI score (Figure 10B). The publication bias of the continuous PASI score was significant in Egger’s test, but no significant bias was confirmed in Begg’s test (Egger’s test: t = −6.58, df = 67, *p* < 0.0001; Begg’s test: z = 1.19, *p* = 0.2335).

### 3.10. Summary of Evidence according to Outcome Measures

For all outcome measures in the IM compared to CM trials, the overall quality of the evidence ranged from very low to moderate. Table 4 shows the results of the GRADE assessments.

### 3.11. Core Herbs Discovery Based on Data Mining

#### 3.11.1. Detailed Information on Investigational Medicine Ingredients

A total of 137 EAHMs were used as components of IM in the 126 clinical trials included in this review. Supplementary Table S2 provides comprehensive details on the herbal components of IM. The following 26 individual herbs, listed in descending order of frequency, were among those used in 10% of IM prescriptions: *Rehmannia glutinosa* (Gaertn.) DC., *Glycyrrhiza uralensis* Fisch., *Dictamnus dasycarpus* Turcz., *Paeonia × suffruticosa* Andrews, *Paeonia anomala* subsp. *veitchii* (Lynch) D.Y.Hong and K.Y.Pan, *Smilax glabra* Roxb., *Angelica sinensis* (Oliv.) Diels, *Lonicera japonica Thunb.*, *Saposhnikovia divaricata* (Turcz.) Schischk., *Arnebia euchroma* (Royle) I.M.Johnst., *Sophora flavescens Aiton*, *Isatis tinctoria* subsp. *athoa* (Boiss.) Papan., *Scrophularia ningpoensis* Hemsl., *Salvia miltiorrhiza Bunge*, *Cryptotympana dubia* (Haupt), *Carthamus tinctorius* L., *Styphnolobium japonicum* (L.) *Schott*, *Spatholobus suberectus* Dunn, *Arctium lappa* L., *Scleromitrion diffusum (Willd.) R.J.Wang*, *Paeonia lactiflora* Pall., *Ligusticum striatum* DC., *Ophiopogon japonicus* (Thunb.) Ker Gawl., *Scutellaria baicalensis* Georgi, *Scutellaria Nepeta tenuifolia* Benth., and *Taraxacum mongolicum* Hand.-Mazz. The relative frequencies of the top 26 herbal materials ranged from 10.16% to 62.5%. Table 5 provides detailed information on the frequency distributions of EAHM materials.

#### 3.11.2. Social Network Analysis

**Figure 11:**
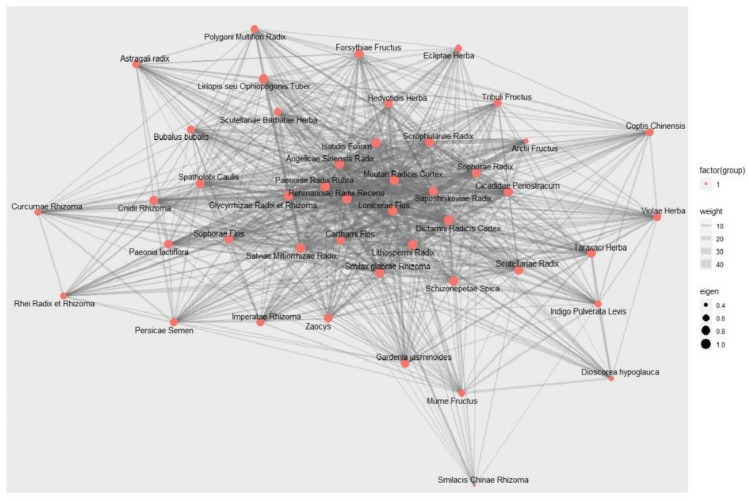
IM herbal materials network used in more than 5% of trials for inflammatory skin lesion of psoriasis.

Social network analysis was conducted on materials that were utilized regularly in more than 5% of trials to examine the correlation and centrality among herbal medicines prescribed to patients, as well as to identify the candidate sovereign herbs (Figure 11). In more than 5% of the trials, a total of 45 herbal medicines were included in the analysis. Centrality analysis showed that the range of the eigenvector centrality index of all materials was in the range of 0.338 to 1, and the range of the centrality index according to the PageRank method was found to be 0.0106 to 0.0284 (Table 5 and Table S3). The core materials that exceeded 0.9 in the eigenvector centrality were as follows: *Rehmannia glutinosa* (Gaertn.) DC., *Salvia miltiorrhiza* Bunge, *Smilax glabra* Roxb., *Glycyrrhiza uralensis* Fisch., *Paeonia anomala* subsp. *veitchii (Lynch)* D.Y.Hong and K.Y.Pan, *Angelica sinensis* (Oliv.) Diels, *Paeonia × suffruticosa* Andrews, *Dictamnus dasycarpus* Turcz., *Arnebia euchroma* (Royle) I.M.Johnst., *Lonicera japonica* Thunb., *Saposhnikovia divaricata* (Turcz.) Schischk., *Cryptotympana dubia* (Haupt), *Isatis tinctoria* subsp. *athoa* (Boiss.) Papan., *Spatholobus suberectus* Dunn, *Sophora flavescens* Aiton, and *Scrophularia ningpoensis* Hemsl. Only these materials exceeded 0.026 in the PageRank centrality, and there was no change in the ranking trend. These materials have the strongest reciprocity based on the properties of the prestige centrality. Therefore, they were considered as candidate sovereign herbs in this review.

#### 3.11.3. A Priori Algorithm-Based Association Rule Analysis

**Figure 12:**
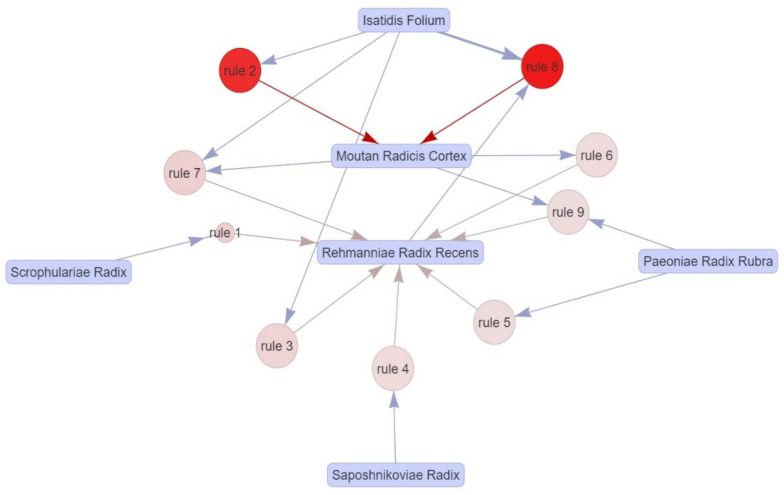
Network graph of the core association rule in the IM component herbs prescribed for inflammatory skin lesion in psoriasis.

Nine association rules were discovered via a priori algorithm analysis of the ingredient data for the 137 herbs included in this review (Table 6). Among the investigated association rules, the following three patterns showed the highest confidence value: # 1 {*Scrophularia ningpoensis* Hemsl.} => {*Rehmannia glutinosa* (Gaertn.) DC.} (confidence:0.9697); # 7 {*Isatis tinctoria* subsp. *athoa* (Boiss.) Papan., *Paeonia × suffruticosa* Andrews} => {*Rehmannia glutinosa* (Gaertn.) DC.}; # 8 {*Isatis tinctoria* subsp. *athoa* (Boiss.) Papan., *Rehmannia glutinosa* (Gaertn.) DC.} => {*Paeonia × suffruticosa* Andrews}. The following two association rules had lift values higher than 2: # 2 {*Isatis tinctoria* subsp. *athoa* (Boiss.) Papan.} => {*Paeonia × suffruticosa* Andrews} (Lift:2.277) and # 8 {*Isatis tinctoria* subsp. *athoa* (Boiss.) Papan., *Rehmannia glutinosa* (Gaertn.) DC.} => {*Paeonia × suffruticosa* Andrews} (Lift:2.342). Therefore, four association rules with excellent characteristics, as described above, were selected as the most important patterns that can be confirmed among IM prescriptions. The relationship diagram of all the association rules is presented as a separate network graph (Figure 12).

#### 3.11.4. Derivation of Core Herbs

Based on the above three-step data mining, four core herbs, *Rehmannia glutinosa* (Gaertn.) DC., *Isatis tinctoria* subsp. *athoa* (Boiss.) Papan., *Paeonia × suffruticosa* Andrews, and *Scrophularia ningpoensis Hemsl.*, were selected to simultaneously satisfy the three conditions defined in the research method section. Table 7 shows the results calculated using the inter-quartile range, with the extreme values removed for the range of dose and administration period of each material based on the data of the included trial.

### 3.12. Analysis of Four Core Herbs through Network Pharmacology

#### 3.12.1. Active Ingredients and Anti-Psoriasis Targets of Four Core Herbs

**Figure 13:**
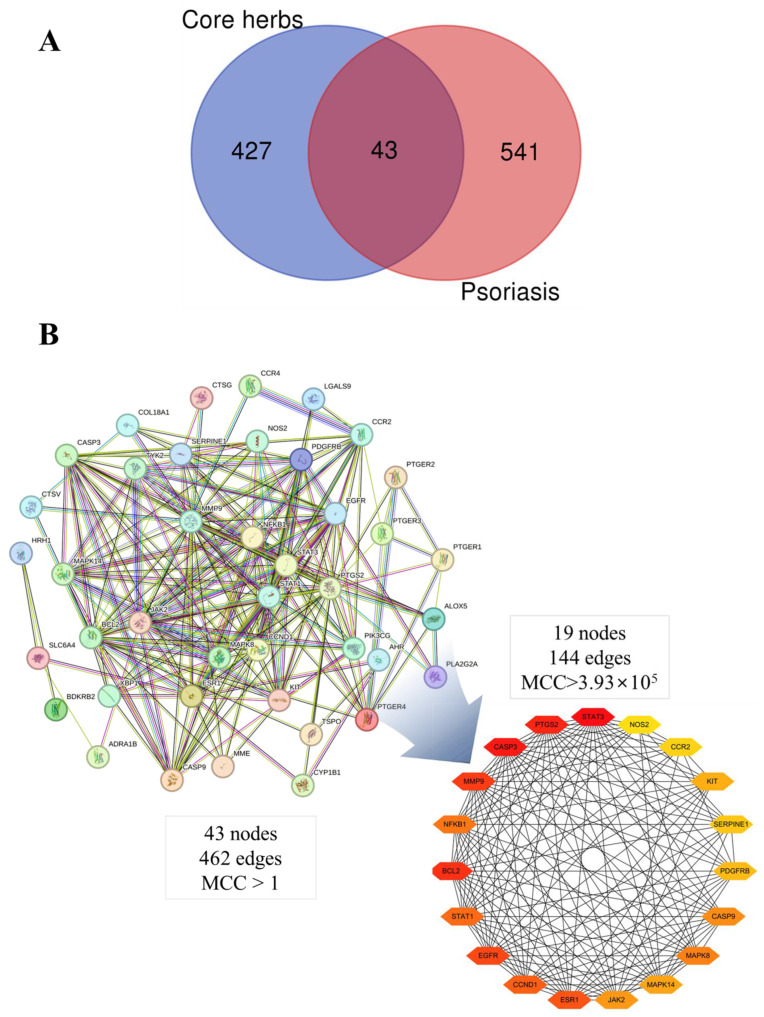
(**A**) Venn diagram of targets of the four core herbs against psoriasis; (**B**) PPI network construction sequence of four core herbs against psoriasis gene targets by MCC algorithm; MCC: Maximum Clique Centrality.

The chemical ingredients of four core herbs were retrieved from a traditional Chinese medicine system pharmacology database (TCMSP), an encyclopedia of traditional Chinese medicine (ETCM), a high-throughput experiment-and-reference-guided database of traditional Chinese medicine (HERB), and a traditional Chinese medicine integrative database (TCMID). For the above compounds collected from multiple databases, after completing the first screening by utilizing OB and DL information from TCMSP DB, we conducted a second screening by performing an ADME prediction for each compound and finally derived a list of 22 potential active compounds. (Table 8) We then performed target prediction by compound on the SwissTargetPrediction platform and obtained pieces of 473 compound–target relationship data after removing duplicates (Supplementary Table S4). Additionally, the GeneCards database contained information on 584 human target genes associated with *psoriasis vulgaris* (Supplementary Table S5). After intersection mapping, 43 consensus genes were identified as potential therapeutic targets for four core herbs against psoriasis (Figure 13A).

#### 3.12.2. PPI Network Construction

Using the STRING 11.5 platform, we imported common targets and constructed the PPI network model (minimum required interaction score: 0.4) by restricting the organism to “*homo sapiens*”. A total of 41 nodes with 462 edges were obtained with an average degree of 11.268. Two targets (CA3, CHRNA7) were excluded from the PPI network because they did not interact with other targets. The MCC algorithm was used by cytoHubba to screen the main hub genes. The outcome was the identification of 19 nodes as hub gene targets, each of which had an MCC score at least three times higher than the median value 3.93 × 10^5^). Table 9 lists the 19 hub gene targets according to their degree centrality. Figure 13B shows the PPI network of hub targets.

#### 3.12.3. GO and KEGG Pathway Enrichment Analyses

**Figure 14:**
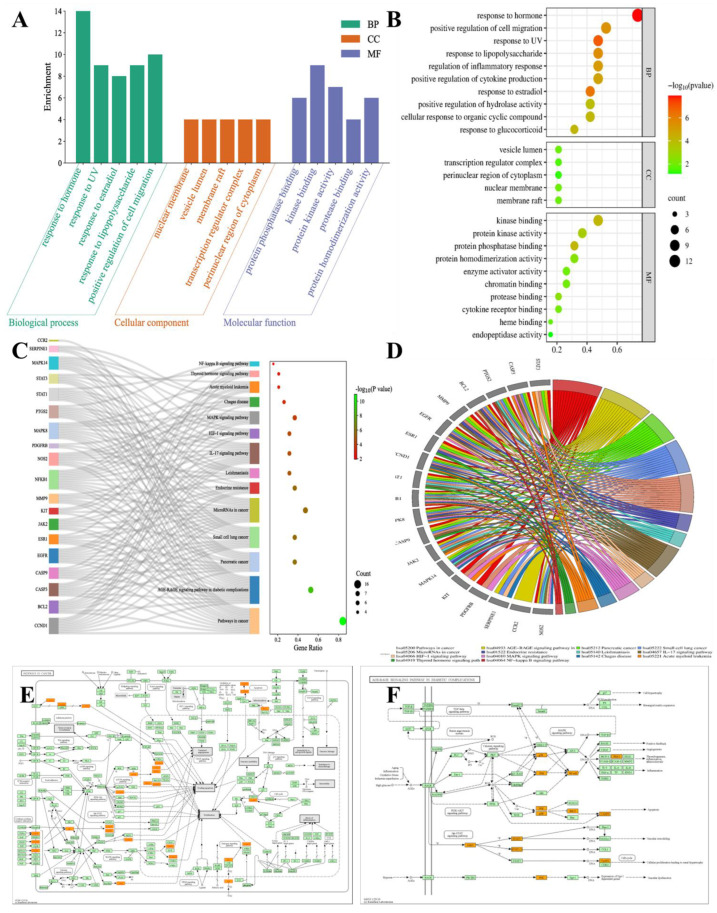
(**A**) Top 5 of GO enrichment analysis for biological process, cellular components, and molecular functions.; (**B**) Bubble plot of GO enrichment; (**C**) Sankey and dot plot of KEGG pathway enrichment analysis illustrating 8 enriched pathways; (**D**) Gene ontology chord diagram of KEGG pathway analysis; (**E**) Pathways in cancer were colored using the KEGG mapper. (**F**) AGE-RAGE signaling pathway in diabetic complications were colored using the KEGG mapper. Orange represents the therapeutic targets in this pathway where the four core herbs act to alleviate psoriasis.

The results of GO and KEGG analyses of the top 19 hub targets are shown in Figure 13A–E. A total of 312 biological processes were identified, including the response to hormones, response to UV, response to estradiol, response to lipopolysaccharide, positive regulation of cell migration, the regulation of inflammatory response, positive regulation of cytokine production, response to glucocorticoid, cellular response to an organic cyclic compound, and the positive regulation of hydrolase activity (Figure 14A,B). A total of 21 molecular functions were identified, including protein phosphatase binding, kinase binding, protein kinase activity, protease binding, protein homodimerization activity, cytokine receptor binding, chromatin binding, enzyme activator activity, heme binding, and endopeptidase activity (Figure 14A,B). Five cellular components were identified, including nuclear membrane, vesicle lumen, membrane raft, transcription regulator complex, perinuclear region of cytoplasm (Figure 14A,B). A total of 104 pathways were identified using KEGG pathway analysis. The results suggested that the mechanisms of four core herbs were mainly linked to pathways in cancer, the AGE-RAGE signaling pathway in diabetic complications, pancreatic cancer, small cell lung cancer, MicroRNAs in cancer, Endocrine resistance, Leishmaniasis, the IL-17 signaling pathway, HIF-1 signaling pathway, MAPK signaling pathway, Chagas disease, Acute myeloid leukemia, Thyroid hormone signaling pathway, and the NF-kappa B signaling pathway (Figure 14C–E).

#### 3.12.4. Construction of Compounds–Target-Pathway Network of Four Core Herbs against Psoriasis

**Figure 15:**
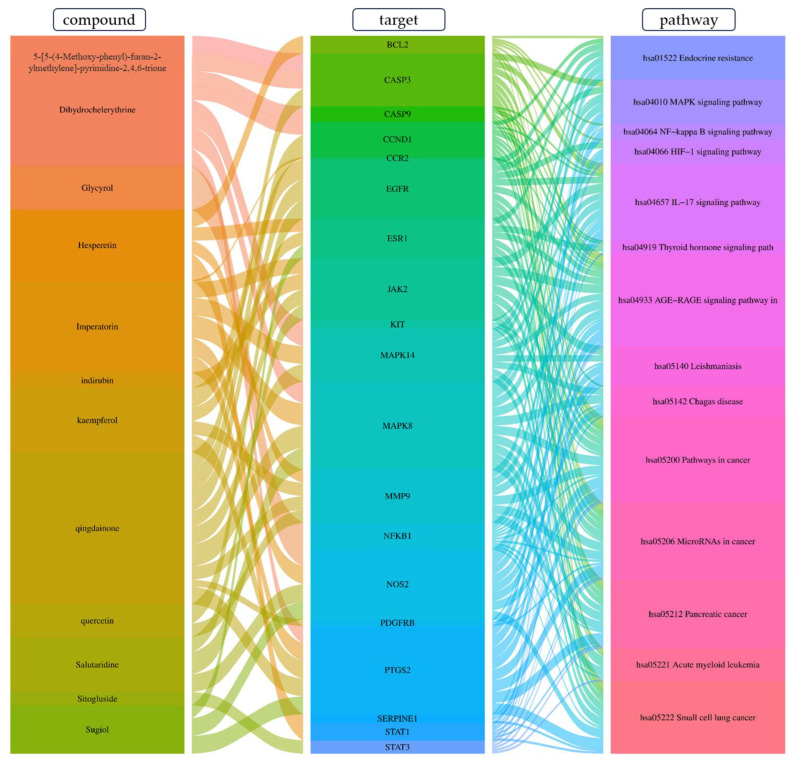
Alluvial plot showing the compound–target-pathway network for the therapeutic mechanism of psoriasis from four core herbs.

To intuitively illustrate the compound–target-pathway relationships between the four core herbs and psoriasis, we used a network visualization method, Alubian plots. As shown in Figure 15, the network contained 45 nodes (including 12 compound nodes, 19 target nodes, and 14 pathway nodes) and 652 edges. The compounds in the network, qingdainone, Dihydrochelerythrine, Imperatorin, Hesperetin, kaempferol, Salutaridine, Sugiol, Glycyrol, quercetin, 5-[5-(4-Methoxy-phenyl)-furan-2-ylmethylene]-pyrimidine-2,4,6-trione, indirubin, and Sitogluside, showed degree centrality values of 69, 51, 42, 32, 29, 25, 22, 20, 15, 8, 7, and 6, respectively. Among therapeutic targets, PTGS, MAPK8, and NOS2 were identified as important nodes with high-degree centrality values of 80, 80, and 64, respectively. In the case of pathways, pathways in cancer (hsa05200) and the AGE-RAGE pathway in diabetic complications (hsa04933) showed high values of 39 and 42, respectively.

## 4. Discussion

### 4.1. Summary of the Main Findings and Comparision with Previous Research

According to the clinical trial data analyzed in this study, IM can have a superior effect compared to CM in the improvement of skin damage as measured by PASI as well as inflammation-related biomarkers including TNF-α, IL-8, IL-17, IL-22, IL-23, and IFN-γ. Through integrated data mining to explore the core herbs that contribute most to this effect, *Rehmannia glutinosa* (Gaertn.) DC., *Isatis tinctoria* subsp. *athoa* (Boiss.) Papan., *Paeonia × suffruticosa* Andrews, and *Scrophularia ningpoensis* Hemsl. were identified as the four core herbs. To explore their therapeutic mechanisms for psoriasis from a holistic perspective, we further conducted network pharmacology analysis and found that the multiple bioactive compounds contributed to the clinical effects, mainly based on their actions on 19 gene targets and 14 signaling pathways.

The above results can be compared to previous studies we have conducted. Previously, we analyzed data from clinical trials comparing the efficacy of CM with the oral administration of EAHM alone and explored the core herbal materials through frequency analysis and network analysis [215]. In that study, EAHM showed superiority in PASI 70, PASI 60, and continuous PASI scores compared to the CM group. It also showed significant results in immune-mediated inflammatory markers such as IL-17, IL-23, and TNF-α, and in the incidence of adverse events. This supports the clinical benefit of IM identified in this study. On the other hand, the data mining used to extract the core drugs in the previous study is different from the methodology of this study, and no separate pharmacopredictive analysis was performed, making a direct comparison difficult. However, *Rehmannia glutinosa* (Gaertn.) DC. *and Paeonia × suffruticosa* Andrews were classified as core drugs in EAHM monotherapy. *Isatidis radix*, a plant of the same origin as *Isatis tinctoria subsp. athoa* (Boiss.) Papan. in this study, but administered at different sites and with almost similar traditional indications, was also a key drug in EAHM monotherapy [216]. Despite the completely different nature of EAHM monotherapy and its combination with CM, the findings of previous studies that similar drugs are important in psoriasis provide some support for the conclusion that the four core herbs in this study exert important pharmacological effects that represent the overall clinical efficacy of IM.

Compared to other relevant previous studies, the favorable effect of IM on psoriasis demonstrated in this study reveals a consistent conclusion [56,57,58,59,60]. However, the existing systematic reviews for simply evaluating the effectiveness of IM or EAHM monotherapy performed an analysis of the overall clinical endpoints related to psoriasis. Additionally, these previous studies did not limit the administration route and application form of the interventional drugs. Considering this, here we evaluated whether EAHM could be a useful candidate for a new IM drug, with a specific target, by setting strict criteria for the administration route and whether to use it as a combined treatment. As for the evaluation index, only the range that specifically supports the indication of “inflammatory skin lesion” was adopted, and not overall psoriasis. The results of this perspective analysis provided a favorable answer to the question of whether the whole EAHM used as an IM, including core herbs, may be a promising candidate material for inflammatory skin lesions caused by psoriasis.

On the other hand, owing to the nature of psoriasis, which has a complex underlying mechanism and a high incidence of comorbid diseases, safety concerns due to polypharmacy are important topics to be addressed [217,218]. Additionally, a detailed evaluation of the relationship between this dosage form and certain adverse effects, particularly drug-induced liver damage, is required owing to the nature of IM, which requires combination therapy with CM. In this study, taking into consideration the aforementioned issues, the incidence rates of AEs were grouped and compared by the lineage in which the symptoms developed. The findings of the analysis demonstrate that IM can be a safer option than CM in the treatment of psoriasis by reducing the incidence of several AEs, such as drug-induced liver damage, cutaneous symptoms, and metabolic disorders. In addition, this justifies the development of a new EAHM drug for patients with poor adherence to CM.

Overall, based on the information from this study, EAHM can be considered worthy to continue its research, as a more effective and safer IM drug candidate for inflammatory skin lesions in psoriasis.

### 4.2. What Are the Limitations and Future Tasks of This Study?

The long history of EAHM administration on humans in numerous countries is perhaps the most substantial advantage of using them to identify potential new drug candidates. If the source data for drug discovery are confined to the classical literature or the experience of individual clinicians, the reproducibility of this information should be questioned. Encouragingly, the number of clinical trials using EAHM has increased significantly over the past decade. Our study reflects the quantitative growth of IM-related clinical and pharmacological data on psoriasis. However, due to data quality issues and methodological limitations not fully addressed in this study, considerable caution should be taken in interpreting the results.

The recent literature reflects the advances that have been made in research methodologies and suggests several requirements for optimized herbal medicine research [219]. Among them, the reproducibility/consistency of the results of the investigation is presented as an important factor. With regard to this requirement, the present study has limitations that cannot be overlooked. Despite the increase in the number of clinical trials identified in this review, there is still a significant risk of bias due to issues such as lack of pre-protocol enrollment, uncertainty in randomization methods, and selective outcome reporting. In addition, the lack of chemical and quality control information in all studies, which is essential for natural medicine trials, is an important reason to question the reproducibility of inferred IM efficacy. However, this is exactly the problem that this study aims to address. There are currently hundreds of EAHMs with pharmaceutical status, and it is unclear which individual herb or herbal preparations are worthy candidates for further study in psoriasis. Therefore, selecting more promising candidates and focusing resources on them according to the principle of selection and concentration to obtain higher quality and reproducible clinical evidence is a key challenge for future research and the aim of this manuscript. In the same sense, it is appropriate to accept the information in this study only at the level of a useful research hypothesis proposal that is relatively consistent with subsequent studies.

Second, the literature recommends the use of network pharmacology in herbal medicine research, but the results should be validated by in vivo experiments or clinical trials. The present study partially satisfies the above conditions in that it derived herbs that are believed to play a key role in a number of clinical trials and performed network pharmacology analysis on them. Although the four core herbs derived in this study showed a pattern of superiority in the data science characteristics based on clinical utilization information compared to other drugs, their pharmacological activity against psoriasis needs to be further verified by separate follow-up animal experiments to show their superiority over other drugs. Meanwhile, the methodology of network pharmacology itself needs to be improved. For example, during the course of this study, we recognized the risk that compound screening methodologies that rely solely on information provided by specific network pharmacology databases may not be updated or may erroneously lead to assays based on compounds that are not actually druggable. This would render the overall predictive results of Network Pharmacology unreliable. Therefore, future network pharmacology studies should be conducted in a manner that recognizes the potential for such errors in the information provided by existing databases and has measures in place to compensate for them.

Third, the specification of synergistic or antagonistic interactions of multi-component preparations was also presented as a very important condition for herbal medicine research. In particular, in the case of EAHM, which is the subject of this study, maximizing the synergistic effects between drugs in a multi-herb combination is a theoretical principle of clinical practice, and most clinical trials have adopted such a combination as an intervention. However, there are limitations due to the scope of the design of this study that prevented us from exploring these interaction effects in more depth. Furthermore, this study examined combination therapy with CM as an intervention, not EAHM monotherapy. The most appropriate CM that can be used in combination with core herbs could not be identified, and various expected interactions could not be considered. The most important issue to be addressed in relation to combined pharmacotherapy is the safety issue due to drug–drug interactions. Overall, this study’s meticulous examination of all types of adverse events concluded that IM would benefit patients with psoriasis in terms of safety. Further studies of IM will require a more detailed analysis of the interactions between specific CMs and the candidate EAHM materials that will make up the actual IM.

Fourth, the four core herbs and their predicted pharmacodynamics in this study do not fully represent the full spectrum of EAHM efficacy for psoriasis. This approach was chosen to address some of the weaknesses of systematic reviews of EAHM efficacy in general, including a lack of information on the mechanisms of action and on key drug identification. This allowed us to filter out drugs that were likely to support the concept of a sovereign herb in EAHM, at least in terms of drug prescription data that reflected clinical implications, and that had prominent data science characteristics compared to many other individual herbs. We also used network pharmacology techniques to confirm that these potential sovereign herbs do indeed have pharmacological activities consistent with the efficacy of EAHM for psoriasis. However, this study cannot answer the question of whether herbs that exhibit prominent patterns and frequencies in data mining are likely to be actual theoretical EAHMs. Furthermore, due to the nature of psoriasis as an immune-mediated inflammatory disease, the targets and pathways involved in the pathology are far beyond the scope of modulation by the four core herbs in this study. Therefore, the methodology attempted in this study should be further developed through continuous follow-up and validation, and the results should not be interpreted as more than a high-quality research hypothesis.

In addition to the BLT4s discussed above, other factors important in the pathogenesis of psoriasis are being studied, including cytoplasmic phospholipase A2, glycerophosphodiester phosphodiesterase domain containing 3, arachidonate 12-lipoxygenase R type, phospholipase B-like 1, sphingomyelin phosphodiesterase 3, ganglioside GM2 activator, and serine palmitoyltransferase long chain subunit have been suggested as important gene targets to consider for future studies.

### 4.3. Implications of the Four Core Herbs That Emerged from This Study

The notable feature of this study is the discovery of four core herbs as a promising candidate material for further investigation using clinical trial data. It is anticipated that this idea can be employed as an effective research hypothesis in subsequent experiments for the following reasons.

EAHM is different from the experience of using other natural medicinal products in that the principle of use is to incorporate a polyherbal formulation that can maximize the synergistic effect. “Gun-Shin-Jwa-Sa” theory, which in WHO standard nomenclature is known as a sovereign–minister–assistant–courier, is the formulation theory for the ideal combination of EAHM [30]. Among them, the drug responsible for the main pharmacology is called a sovereign or monarch drug. Due to the impact of this sovereign drug, EAHM prescriptions may be taken as a single medication, and the remaining drugs both minimize the overall prescription’s toxicity and enhance the activity of sovereign drugs [24,61,75].

The sovereign drug concept can be explained in two ways. First, identifying a sovereign drug in the EAHM formula, in which several materials are combined, is to screen candidates that represent the overall pharmacology of the prescription, so it is possible to effectively narrow down the dimension of the hypothesis for actual drug discovery. In addition, based on the premise that it is a sovereign drug that represents the mechanism of action of EAHM for a specific disease, an in-depth exploration of the mechanism of action of this material can provide more refined information for subsequent research.

However, the criteria used to identify this sovereign herb are ambiguous and the general system depends on the clinician’s tacit knowledge. For example, the material with the highest capacity may not be a sovereign herb; conversely, the material used in a very small amount may be a sovereign herb. Consequently, the scientific research methodology to address this problem has not yet reached its full potential.

As a result, we focused on how to recognize such four core herbs as the most essential hypothesis for IM drug discovery in this work. In the authors’ previous study, four core herbs was selected only by social network analysis of the frequently prescribed drug group and the centrality index calculated from it [220]. In contrast, this study offered four core herbs of a more specifically compressed range compared to previous research by combining several data-mining approaches and choosing materials that exhibit essential features across all procedures as intersections.

### 4.4. Possible Pharmacological Mechanisms of Four Core Herbs aginst Psoriasis

#### 4.4.1. Pharmacology of Individual Crude Herbs to Support Efficacy

The greatest strength of EAHM research is that it is currently used as a medicine in many East Asian countries. In other words, because crude EAHM is used as a drug, drug data such as human dose, administration period, and prescription trend by the disease can be obtained in a more direct form, giving it a comparative advantage in terms of safety as a candidate material for new drug research. In addition, the effects of polysaccharides and other phytochemicals with low bioavailability, which are difficult to predict using only compound-based pharmacological studies because of the nature of natural products, cannot be overlooked. Therefore, the development of botanical drugs using crude materials in EAHM research remains one of the most important topics.

The materials derived from the core herbs in this study were *Rehmannia glutinosa* (Gaertn.) DC., *Isatis tinctoria* subsp. *athoa* (Boiss.) Papan., *Paeonia × suffruticosa* Andrews, and *Scrophularia ningpoensis* Hemsl., an EAHM that has been widely used for inflammatory and autoimmune diseases for a long time owing to its broad immunomodulatory effects. It can also reduce oxidative stress and inhibit apoptotic responses [221,222]. One study regarding the topical application of *Rehmannia glutinosa* (Gaertn.) DC. extracts to an atopic dermatitis animal model reported that skin inflammation was suppressed through the inhibition of dermal infiltration by inflammatory cells and the suppression of chemokine production by keratinocytes [223]. *Isatis tinctoria* subsp. *athoa* (Boiss.) Papan. has recently been attracting attention for its antiviral activity against various viral infections, including COVID-19, and its related effects, such as a wide range of anti-endotoxin, anti-inflammatory, and immune regulation activities, have also been confirmed [224,225]. Recently, it was reported that the anti-aging effect of this material is based on its antioxidant and anti-inflammatory effects [226].

*Paeonia × suffruticosa* Andrews is the most widely used material for various skin inflammatory reactions and is expressed in redness and dryness [227]. As for its direct effect on psoriasis, it has been reported that the improvement of psoriasis-like skin lesions in a mouse model occurs through the inhibition of the mRNA expression of IL-23 and the activation of dendritic cells via actions on the toll-like receptor 7/8 signaling pathway [228]. Furthermore, it is well recognized that the *Paeonia × suffruticosa* Andrews has a variety of benefits for atopic dermatitis-related inflammation, dryness, and UV-induced skin aging [229,230,231]. *Scrophularia ningpoensis* Hemsl. is a material known for its role in various inflammatory diseases based on its pharmacological effects across anti-inflammatory, immune-enhancing, anti-apoptotic, and anti-allergic effects [232,233]. *Scrophularia ningpoensis* Hemsl. extract effectively inhibited inflammatory cell infiltration and the expression of IL-5, IL-13, IL-17, and immunoglobulin E, as well as reduced NF-κB phosphorylation, thereby exhibiting an anti-inflammatory effect [234].

As the four core herbs have a wide range of anti-inflammatory and immunomodulatory properties, it may be a promising candidate material for successfully reducing a variety of skin inflammatory consequences. At the same time, the assumption of the four core herbs’ identification in this study will be a methodology to identify materials that play a key role in the manifestation of clinical effects found throughout IM clinical trial data which are also convincing.

#### 4.4.2. The Key Therapeutic Targets and Pathways Associated with the Four Core Herbs

It is widely known that the mechanism of action of natural products, including East Asian herbal medicine, is mainly based on the action of multi-compound multi-target/signaling pathways [235,236,237]. In addition, the synergistic effect generated in this action process further enhances the potential of natural product pharmacology [238]. However, it is not easy to interpret and predict such a wide range of action unless a separate research methodology is used for such multilayered and multifaceted pharmacology. Therefore, in this study, a network pharmacology technique was additionally used for an exploratory analysis of the range of mechanisms of action for psoriasis of the derived four core herbs. The active compounds of the four core herbs that treat psoriasis were predicted to act on 19 gene targets, including STAT3, CASP3, PTGS2, BCL2, MMP9, EGFR, ESR1, CCND1, and STAT1, by PPI analysis. Based on this, as a result of KEGG pathway enrichment analysis, eight pathways were identified to be related to the psoriasis treatment effect of four core herbs. Among them, the pathways expected to be most relevant to important therapeutic gene targets are the “pathways in cancer” and “AGE-RAGE signaling pathway in diabetic complications”. These include a number of very complex partial signaling pathways. Thus, the detailed relationship between the target and the pathway was additionally explored using the KEGG mapper to more specifically clarify where the psoriasis treatment effect occurs.

The major gene targets of four core herbs, MMP9, BCL2, STAT1, and STAT3, each as a subset of pathways in cancer, were confirmed to be involved in the MAPK pathway, the mTOR pathway, and cytokine–cytokine receptor interaction. These are all pathways involved in proliferation. Pharmacological action on these pathways may be related to the alleviation of psoriasis. For example, previous studies in which metformin was applied to the MAPK and mTOR pathways showed the significant inhibition of cell proliferation and inflammatory responses in human immortalized keratinocytes [239,240]. In view of this, it is not difficult to predict that various natural products that act on the MAPK pathway will show significant psoriasis treatment effects, and consistent progress and multiple previous studies have reported this [241,242]. Several recent studies on the mTOR signaling pathway have identified it as an important regulatory target for the treatment of the epidermal and functional structure in dermatologic diseases, including psoriasis, and relatedly, there are reports that natural products that can modulate this signaling pathway have shown good efficacy in ameliorating animal models of psoriasis with dyslipidemia [243,244]. Meanwhile, STAT1 and STAT3 have been shown to be involved in the cytokine–cytokine receptor interaction pathway, and indeed, these targets are involved in suppressing the pathology of immune-mediated inflammation in psoriasis. For example, blocking the STAT1 function is known to suppress the activity of pathogenic Th1 cells and induce the hyperactivation of Th17 cells, thereby suppressing psoriasis caused by them [245]. On the other hand, in the case of STAT3, previous research reports have been made stating it to be a mediating pathway of alleviating skin inflammatory damage of psoriasis through the anti-inflammatory and antioxidant activities of natural products [246]. CASP3 and CASP9 have been shown to be involved in the regulation of the apoptotic pathway, and given that impaired apoptosis is an important contributor to keratinocyte proliferation abnormalities in psoriasis pathology, it is conceivable that the inhibition of these pathways may be related to similar mechanisms of action as in proliferative pathologies [247]. Based on the above, it is possible to interpret that the activity of the four core herbs on pathways in cancer is mainly exerted through the mechanism of the inhibition of the abnormal proliferation of keratinocytes. This is also the pharmacological mechanism of many natural products that have been shown to alleviate psoriasis, a finding that is relatively consistent with the results of the clinical trial data [248].

The major gene targets of the four herbs in relation to AGE-RAGE pathways include JNK, BLC2, CASP3, NFKB1, JAK2, STAT1, and STAT3, which have been implicated in the regulation of PI3K-AKT, JAK/STAT, and MAPK pathways as partial signaling pathways. Among these, the PI3K-AKT pathway is important as it is involved in the development of general immune-mediated inflammatory diseases. Notably, mTOR kinase, a downstream effect of this pathway, is over-activated during the course of psoriasis pathology, promoting keratinocyte proliferation and suppressing differentiation [249]. Additionally, when the immune-mediated inflammatory state of psoriasis is dysregulated, mTOR signaling is activated in keratinocytes. Hence, the PI3K-AKT-mTOR pathway is an important component of psoriasis pathology in which immune and inflammatory pathologies are linked to keratin hyperplasia. In this context, among the natural products and phytochemicals effective in psoriasis, a number of cases showing a correlation with the PI3K-AKT-mTOR pathway have already been reported in previous studies [250]. The JAK/STAT pathway has also been implicated in the pathophysiology of psoriasis, involving the proliferation of Th17 cells, keratinocytes, and γ- δ T cells [251]. It is similar to the PI3K-AKT pathway above in that it is activated by cytokines such as IL-17 and IL-23 and serves as a link between immune-mediated inflammatory pathology and the overproduction of epidermal keratinocytes. Therefore, the JAK/STAT pathway can be considered as one of the other major targets for the treatment of psoriasis with natural products [252].

In summary, the multiple pathways of action of the four core herbal targets extracted from the herbal prescription data of 126 clinical trials are all closely related to the actual pathogenesis of psoriasis, and their effects appear to be centered on modulating pathologic keratinocyte proliferation and inhibiting immune-mediated inflammation. This is meaningful because it is consistent with the inhibitory effects of PASI and various inflammatory cytokines seen in clinical trial results. Therefore, it may be worthwhile to conduct intensive non-clinical and clinical follow-up studies on the multi-component, multi-pathway mechanisms of the four core herbs in suppressing psoriasis.

#### 4.4.3. Promising Therapeutic Targets in Psoriasis Unexplained by the Pharmacology of Four Core Herbs

As described above, the four core herbs in this study exert modulatory effects on key pathologies of psoriasis through broad multi-target, multi-pathway pharmacological modulation. However, recent studies have reported promising therapeutic targets for psoriasis beyond the scope of the four core herbs.

Notable among these are the leukotriene B4 (LTB4) receptors, BLT1 and BLT2. BLT1 blockade has been shown to treat immune-mediated inflammatory diseases such as rheumatoid arthritis and asthma, and should be strongly considered in the treatment of psoriasis [253]. Recent studies in this regard include a report on the most widely used EAHM with potent anti-inflammatory activity, *Coptis chinensis* Franch. [254]. This study showed that *Coptis chinensis* Franch. can inhibit the pathology of immunosuppressive cytokine secretion by tumor-associated macrophages that cause immune checkpoint blockade (ICB) treatment for cancer to fail. In particular, the blockade of LTB4 signaling and inhibition of ICB were observed as one of the important mechanisms, and this study confirmed that the combination of *Coptis chinensis* Franch. and anti-cancer immunotherapy may lead to better results. Although this herb is not used in the drug data included in this study, it can be considered as a promising candidate for psoriasis treatment considering the above mechanisms of action. On the other hand, BLT2 is a low affinity receptor and agonists against it are considered to be able to treat a number of skin diseases. On a related note, another EAHM, branches of *Morus alba* L. (Mori ramulus), has been reported to exert antioxidant and anti-inflammatory activity by blocking BLT2-dependent NADPH oxidase 1 and inhibiting the production of IL-6 [255]. It is worth noting that this is a mechanism that may also be useful for inhibiting inflammation-mediated skin damage in psoriasis, and indeed, the drug data in this study include a prescription for the leaves of *Morus alba* L. (Mori folium) of the same plant. Actually, a number of Korean references also report its use for skin conditions such as hyperpigmentation [256].

In addition to the BLT4s discussed above, other factors important in the pathogenesis of psoriasis are being studied, including cytoplasmic phospholipase A2, glycerophosphodiester phosphodiesterase domain containing 3, arachidonate 12-lipoxygenase R type, phospholipase B-like 1, sphingomyelin phosphodiesterase 3, ganglioside GM2 activator, and serine palmitoyltransferase long chain subunit, which have been suggested as important gene targets to consider for future studies [257]. Immunomodulatory and anti-inflammatory activities via these targets have been reported in EAHMs, but to date, there appears to be a paucity of data. However, based on the above discussion, two directions for further research can be suggested. First, as we have seen, the more complex the disease, the more important the action on key gene targets and pathways involved in the pathology, so it is necessary to conduct efficacy studies for specific indications of EAHM by targeting modulatory actions on newly identified important pathologies. In this case, the multi-target, multi-pathway effects of EAHM, a characteristic and advantage of EAHM, can be reduced in advance by actively using the network pharmacology methodology. In the present study, there were 26 drugs that showed significant patterns in terms of prescription frequency for psoriasis. Further research could be conducted to determine the extent to which these drugs are involved in the important targets of psoriasis discussed above and which pathological endotypes they modulate, which would be a worthwhile endeavor from a drug discovery perspective. Overall, the value of this study is that it provides insights that can be used to narrow down hypotheses in this large EAHM dataset for further research.

## 5. Conclusions

The present study showed that the combination therapy of EAHM and CM for the improvement of inflammatory skin lesions in psoriasis patients is a potentially more effective and safer option compared to CM monotherapy. Further integrated data mining and network pharmacology analysis of the drug data in this study revealed that the four core herbs, *Rehmannia glutinosa* (Gaertn.) DC., *Isatis tinctoria* subsp. *athoa* (Boiss.) Papan., *Paeonia × suffruticosa* Andrews, *and Scrophularia ningpoensis* Hemsl., exerted curative effects on psoriasis by inhibiting keratinocyte proliferation and immune-mediated inflammation based on their effects on multiple targets and pathways directly related to psoriasis. However, the quality of the clinical trial data underlying this study is considered suboptimal in terms of both study design and quality control of the intervention. Therefore, the results should be interpreted with caution, bearing in mind that reproducibility is an issue. Further preclinical efficacy studies of the four core herbs with promising pharmacological properties, as well as data from clinical trials with substantially improved designs that meet CONSORT criteria, are needed to validate the information explored in this study. In this respect, this study may be of value in providing summary hypotheses regarding the value and direction of conducting such follow-up studies.

## Figures and Tables

**Table 1:** Basic demographic data and intervention of studies included in the review.

Included Study (Reference)	Trial Design	Randomization Method	Number of Participants (Male/Female); Age (Mean ± SD)		Interventions		Morbidity Period (Mean ± SD or Range)		Outcome Index (Intergroup Differencies *p*-Value)	Course of Treatment	Adverse Events (Case/Symptom)
			Trial	Control	Trial	Control	Trial	Control			
Che 2004 [164]	Randomized; Single center; Parallel	NR	Both group 75 (41/34) 11–44 y Trial: 45	Both group 75 (41/34) 11–44 y Control: 30	1. Erdonghuoxue decoction (t.i.d.) 2. Immunosuppressant (compound amino peptide tablets 5t, b.i.d.)	1. Immunosuppressant (compound amino peptide tablets 5t, b.i.d.)	Both group 4 m–10 y	Both group 4 m–10 y	1. PASI 60 (*p* < 0.05)	4 w	Trial: 5 AEs Control: 22 AEs Including thirst, hyperhidrosis, pruritus, skin scale, dry lips, cheilitis, dry mouth, dry nose, nausea
Chen 2004 [211]	Randomized; Single center; Parallel	NR	30 (18/12) 32.4 y (16–65 y)	26 (16/10) 33.2 y (17–63 y)	1. Anti-psoriasis formula (q.d.) 2. Immunosuppressant (compound amino peptide tablets 5t, b.i.d.)	1. Immunosuppressant (compound amino peptide tablets 5t, b.i.d.)	6.2 y (3 m-28 y)	6.1 y (2 m-30 y)	1. PASI 60 (*p* < 0.05)	6 w	Trial: 29 AEs (6 xerostomia, 8 dry lips, 5 xeroderma, 4 pruritus, 6 skin scale) Control: 84 AEs (18 xerostomia, 20 dry lips, 17 xeroderma, 13 pruritus, 16 skin scale)
Xu 2005 [186]	Randomized; Single center; Parallel	NR	45 (27/18) Range 11–63 y	30 (18/12) Range 15–57 y	1. Liangxuejiedu decoction (b.i.d.) 2. Immunosuppressant (compound amino peptide tablets 5t, b.i.d.)	1. Immunosuppressant (compound amino peptide tablets 5t, b.i.d.)	3 w–22 y	3 w–22 y	1. PASI 60 (*p* < 0.05)	4 w	Trial: 5 AEs Control: 22 AEs Including skin scale, cheilitis, dry nasal cavity, thirst, hyperhidrosis, pruritus, nausea
Liu 2006 [137]	Randomized; Single center; Parallel	NR	Both group 60 (31/29) Trial: 40 (NR) 33.63 y (16–53 y)	Both group 60 (31/29) Control: 20 (NR) 32.7 y (21–64 y)	1. Jianpiyishen decoction (q.d.) 2. Immunosuppressant (compound amino peptide tablets 5t, b.i.d.)	1. Immunosuppressant (compound amino peptide tablets 5t, b.i.d.)	71.191 m (1–168 m)	70.95 m (3–120 m)	1. PASI 70 (*p* < 0.05)	8 w	Trial: 41 AEs (8 xerostomia, 14 xeroderma, 8 skin scale, 11 pruritus) Control: 71 AEs (17 xerostomia, 18 xeroderma, 18 skin scale, 18 pruritus)
Chen 2007 [92]	Randomized; Single center; Parallel Three arm trial	NR	Both group 222 (139/83) 33 y (19–58 y) Trial (IM): 74	Both group 222 (139/83) 33 y (19–58 y) Control: 74	1. Xiaoyin granule (3.5 g, t.i.d) 2. Acitretin capsule (10 mg, t.i.d.)	1. Acitretin capsule (10 mg, t.i.d.)	Both group 4.6 y (1–18 y)	Both group 4.6 y (1–18 y)	1. PASI 60 (*p* < 0.05)	16 w	Trial: 36 AEs (26 xeroderma, 3 pruritus, 1 skin poignant itch, 5 gastrointestinal discomfort, 1 hepatic dysfunction) Control: 29 AEs (21 xeroderma, 1 pruritus, 2 skin poignant itch, 4 gastrointestinal discomfort, 1 hepatic dysfunction)
Huang 2007 [129]	Randomized; Single center; Parallel; single blind	Simple randomization (envelope concealment method)	49 (30/19) 37 ± 9.12 y	49 (32/17) 38 ± 10.27 y	1. Yinxieling tablet (6t, t.i.d.) 2. Acitretin capsule (0.5 mg/kg/day, q.d.)	1. Acitretin capsule (0.5 mg/kg/day, q.d.)	3.36 ± 5.72 y	3.8 ± 5.44 y	1. PASI 60 (*p* < 0.05) 2. PASI score (*p* < 0.01)	8 w	Trial: 130 AEs (49 xerostomia, 13 xeroma, 21 xeroderma, 11 pruritus, 2 epistaxis, 14 folliculitis, 1 hepatic dysfunction, 19 hyperlipidemia) Control 238 AEs (49 xerostomia, 40 xeroma, 38 xeroderma, 20 pruritus, 8 epistaxis, 40 folliculitis, 8 hepatic dysfunctions, 35 hyperlipidemia)
Zeng 2009 [163]	Randomized; Single center; Parallel; single blind	Simple randomization	50 (30/20) 38.61 ± 13.12 y	50 (29/21) 39.41 ± 14.03 y	1. Xiaoyin granule (3.5 g, t.i.d) 2. Topical corticosteroid (clobetasol propionate ointment, b.i.d.)	1. Topical corticosteroid (clobetasol propionate ointment, b.i.d.)	7.68 ± 5.63 y	6.58 ± 5.92 y	1. PASI 60 (*p* < 0.01) 2. PASI score (*p* < 0.01)	4 w	Trial: 15 AEs Control: 18 AEs Including xeroderma, skin scale, pruritus, erythema, mild stabbing
Cao 2010 [157]	Randomized; Single center; Parallel Three arm trial	NR	40 (24/16) 35.3 y (18–64 y)	40 (25/15) 33.5 y (19–62 y)	1. Yangzhen decoction (200 mL, b.i.d.) 2. Acitretin capsule (0.5 mg/kg/day, q.d.)	1. Acitretin capsule (0.5 mg/kg/day, q.d.)	43.5 m (6 m–33 y)	40.5 m (6 m–30 y)	1. PASI 60 (*p* < 0.01)	12 w	Trial: 43 AEs (22 xeroderma and xerostomia, 9 pruritus, 11 hyperlipidemia, 2 hepatic dysfunction) Control: 77AEs (36 xeroderma and xerostomia, 27 pruritus, 13 hyperlipidemia, 1 hepatic dysfunction)
He 2010 [191]	Randomized; Single center; Parallel	NR	Both group 78 (36/42) 43.4 ± 6.2 y Trial: 39	Both group 78 (36/42) 43.4 ± 6.2 y Control: 39	1. Xiaoranqudan feng (200 mL, b.i.d) 2. Acitretin capsule (25 mg, q.d.)	1. Acitretin capsule (25 mg, q.d.)	Both group 3.6 ± 1.1 y	Both group 3.6 ± 1.1 y	1. PASI 60 (*p* < 0.05) 2. PASI score (*p* < 0.01)	8 w	Trial: 15 AEs (10 xerostomia, 5 headache) Control: 85 AEs (8 hepatic dysfunction, 30 xeroderma, 26 xerostomia, 7 headache, 14 gastrointestinal discomfort)
Hua 2010 [184]	Randomized; Single center; Parallel	NR	Both group 90 (50/40) 32.4 y (18–65 y) Trial: 46	Both group 90 (50/40) 32.4 y (18–65 y) Control: 44	1. Qingyinjiedu decoction (30 mg, q.d.) 2. Acitretin capsule (q.d.)	1. Acitretin capsule (q.d.)	Both group 4.2 y	Both group 4.2 y	1. PASI 60 (*p* < 0.01)	8 w	Trial: 15 AEs (8 skin scale and xerostomia, 5 hyperlipidemia, 2 hepatic dysfunction) Control: 30 AEs (17 skin scale and xerostomia, 10 hyperlipidemia, 3 hepatic dysfunction)
Luo 2010 [207]	Randomized; Single center; Parallel	NR	47 (25/22) 41.5 ± 9.8 y	47 (28/19) 39.7 ± 7.8 y	1. Piminxiao capsule (4c, t.i.d.) 2. Topical corticosteroid (Calcipotriol ointment, b.i.d.)	1. Topical corticosteroid (Calcipotriol ointment, b.i.d.)	9.3 ± 6.8 y	7.9 ± 5.7 y	1. PASI 70 (*p* < 0.05) 2. PASI score (*p* < 0.01) 3. Recurrence rate (*p* < 0.05)	12 w	Trial: No AEs Control: No AEs
Yu 2010 [135]	Randomized; Single center; Parallel	NR	40 (NR) 34.18 y (18–60 y)	40 (NR) 32.30 y (19–58 y)	1. Runzaozhiyang capsule (2 g, t.i.d.) 2. Acitretin capsule (30 mg, q.d.)	1. Acitretin capsule (30 mg, q.d.)	4.2 y (20 d–30 y)	3.8 y (1 m–29 y)	1. PASI 60 (*p* < 0.01) 2. PASI score (*p* < 0.01)	4 w	Trial: 5 AEs (5 nausea) Control: 6 AEs (6 hyperlipidemia)
Liu 2011 [165]	Randomized; Single center; Parallel	NR	Both group 68 (42/26) 35 y (18–65 y) Trial: 38	Both group 68 (42/26) 35 y (18–65 y) Control: 30	1. Fangfengtongsheng powder (6 g, b.i.d.) 2. Acitretin capsule (30 mg, q.d.)	1. Acitretin capsule (30 mg, q.d.)	Both group 5.7 y (2 m–12 y)	Both group 5.7 y (2 m–12 y)	1. PASI 60 (*p* < 0.05)	12 w	Trial: 17 AEs (detailed information NR) Control: 23 AEs (detailed information NR)
Lu 2011 [138]	Randomized; Single center; Parallel	NR	44 (28/16) 34.6 ± 3.82 y	44 (26/18) 33.96 ± 4.26 y	1. Qingrejiedu decoction (400 mL, b.i.d.) 2. Etretinate (0.5 mg, q.d.)	1. Etretinate (0.5 mg, q.d.)	5.63 ± 1.32 y	6.02 ± 1.50 y	1. PASI 60 (*p* < 0.05)	8 w	Trial: 1 AE (1 diarrrhea) Control: 2 AEs (2 hepatic dysfunction)
Tian 2011 [197]	Randomized; Single center; Parallel	Simple randomization (random number table)	30 (12/18) 36.2 ± 9.8 y	30 (15/15) 34.5 ± 10.2 y	1. Qingfeiliangxue decoction (100 mL, t.i.d.) 2. Vitamin C, Vitamin B6 (NR)	1. Vitamin C, Vitamin B6 (NR)	10.4 ± 7.6 y	10.4 ± 7.6 y	1. PASI 60 (*p* < 0.05) 2. PASI score (*p* < 0.05)	60 d	NR
Xu 2011 [208]	Randomized; Single center; Parallel	NR	39 (23/16) 34.5 y	39 (20/19) 36.5 y	1. Qingxuanyin (b.i.d.) 2. Acitretin capsule (20 mg, q.d.)	1. Acitretin capsule (20 mg, q.d.)	15 d–29 y	25 d–31 y	1. PASI 60 (*p* < 0.01) 2. PASI score (*p* < 0.01)	8 w	Trial: 8 AEs (8 diarrhea) Control: 10 AEs (10 xerostomia)
Yao 2011 [141]	Randomized; Single center; Parallel	Simple randomization (random number table)	70 (24/46) 36.5 y (15–62 y)	62 (34/28) 35.2 y (14–65 y)	1. Xiaoyin granule (3.5 g, t.i.d.) 2. Immunosuppressant (compound amino peptide tablets 5t, b.i.d.)	1. Immunosuppressant (compound amino peptide tablets 5t, b.i.d.)	7 d–45 y	10 d–47 y	1. PASI 60 (*p* < 0.05)	8 w	Detailed information NR
Zheng 2011 [91]	Randomized; Single center; Parallel	NR	60 (28/32) 42.1 ± 14.6 y	60 (26/34) 42.3 ± 15.4 y	1. Xiaoyinkeji decoction (100 mL, b.i.d.) 2. Acitretin capsule (20 mg, q.d.)	1. Acitretin capsule (20 mg, q.d.)	7.3 ± 0.9 y	7.5 ± 1.2 y	1. PASI 60 (*p* < 0.05) 2. PASI score (*p* < 0.05)	8 w	Trial: 8 AEs (6 gastrointestinal discomfort, 2 diarrhea) Control: 14 AEs (10 gastrointestinal discomfort with nausea or vomiting, 4 leukopenia)
Jiang 2012 [202]	Randomized; Single center; Parallel; single blind	Simple randomization (random number table)	30 (16/14) 33.37 ± 4.32 y	30 (17/13) 34.69 ± 5.01 y	1. Sendi particles (b.i.d.) 2. Acitretin capsule (10 mg, q.d.)	1. Acitretin capsule (10 mg, q.d.)	4.19 ± 2.77 y	3.98 ± 1.97 y	1. PASI 60 (*p* < 0.05) 2. PASI score (*p* < 0.05) 3. Recurrence rate (*p* < 0.05)	12 w	Trial: 3 AEs (2 loose stool, 1 hepatic dysfunction) Control: 1 AE (1 hepatic dysfunction)
Liu 2012 [143]	Randomized; Single center; Parallel	NR	42 (24/18) 40.00 ± 10.26 y	42 (23/19) 39.00 ± 10.50 y	1. Runzaozhiyang capsule (4c, t.i.d.) 2. Acitretin capsule (30 mg, q.d.)	1. Acitretin capsule (30 mg, q.d.)	3.0 ± 4.5 y	3.0 ± 4.6 y	1. PASI 60 (*p* < 0.05) 2. PASI score (*p* < 0.05)	8 w	Trial: 30 AEs (18 xeroderma, 5 xerostomia, 7 pruritus) Control: 98 AEs (32 xeroderma, 34 xerostomia, 32 pruritus)
Xie 2012 [183]	Randomized; Single center; Parallel	NR	Both group 94 (49/45) 35.5 ± 3.2 y Trial: 49	Both group 94 (49/45) 35.5 ± 3.2 y Control: 45	1. Runzaozhiyang capsule (4c, t.i.d.) 2. Acitretin capsule (20 mg, q.d.) 3. Topical corticosteroid (clobetasol propionate ointment, b.i.d.)	1. Acitretin capsule (20 mg, q.d.) 2. Topical corticosteroid (clobetasol propionate ointment, b.i.d.)	Both group 8.5 y (1 w–18 y)	Both group 8.5 y (1 w–18 y)	1. PASI 60 (*p* < 0.05)	4 w	Trial: 5 AEs (3 hyperlipidemia, 2 gastrointestinal discomfort) Control: 4 AEs (4 hyperlipidemia)
Zhang 2012 [127]	Randomized; Single center; Parallel	NR	40 (24/16) 36.65 ± 9.34 y	40 (27/13) 35.76 ± 10.26 y	1. Yinxie capsule (3c, t.i.d.) 2. Acitretin capsule (20 mg, q.d.)	1. Acitretin capsule (20 mg, q.d.)	16.43 ± 15.36 m	15.88 ± 16.48 m	1. PASI 60 (*p* < 0.05)	8 w	Trial: 19 AEs (detailed information NR) Control: 20 AEs (detailed information NR) Including xerostomia, dry lip, xeroma, pruritus, epistaxis
Zhou 2012a [124]	Randomized; Single center; Parallel	NR	70 (46/24) 38.3 y (19–65 y)	70 (44/26) 33.7 y (16–65 y)	1. Xiaoyin granule (3.5 g, t.i.d.) 2. Acitretin capsule (30 mg, q.d.) 3. 10% zing oxide ointment (b.i.d.)	1. Acitretin capsule (30 mg, q.d.) 2. 10% zing oxide ointment (b.i.d.)	5.6 y (3 m–12 y)	7.6 y (2 m–11 y)	1. PASI 60 (*p* < 0.05)	60 d	NR
Zhou 2012b [182]	Randomized; Single center; Parallel	NR	Both group 120 (49/71) 36.85 ± 6.32 y Trial: 49	Both group 120 (49/71) 36.85 ± 6.32 y Control: 45	1. Xiaoyin granule (3.5 g, t.i.d.) 2. Acitretin capsule (10 mg, q.d.)	1. Acitretin capsule (10 mg, q.d.)	Both group 6.70 ± 0.52 y	Both group 6.70 ± 0.52 y	1. PASI 60 (*p* < 0.05) 2. PASI score (*p* < 0.01) 3. Recurrence rate (*p* > 0.05)	12 w	Detailed information NR
Chen 2013 [153]	Randomized; Single center; Parallel	NR	30 (24/7) 31.2 ± 5.3 y	30 (24/7) 30.8 ± 6.1 y	1. Xiaoyin decoction (b.i.d.) 2. Acitretin capsule (10 mg, b.i.d.)	1. Acitretin capsule (10 mg, b.i.d.)	5.1 ± 2.0 y	5.5 ± 2.3 y	1. PASI 60 (*p* < 0.05) 2. PASI score (*p* < 0.05)	8 w	Trial: 2 AEs (1 xeroderma, 1 gastrointestinal discomfort) Control: 6 AEs (2 xeroderma, 1 xeroma, 1 xerostomia, 1 hyperlipidemia, 1 hepatic dysfunction)
Ding 2013 [146]	Randomized; Single center; Parallel	NR	30 (16/14) 52.32 ± 3.41 y	30 (12/18) 54.32 ± 2.15 y	1. NiupixuanⅡ decoction (NR) 2. Acitretin capsule (0.3–1.0 mg/kg/day, NR)	1. Acitretin capsule (0.3–1.0 mg/kg/day, NR)	10.3 ± 2.2 y	11.1 ± 2.5 y	1. PASI score (*p* < 0.05)	4 w	NR
Mo 2013 [136]	Randomized; Single center; Parallel	NR	56 (33/23) 36.2 y (18–58 y)	54 (33/21) 35.9 y (20–60 y)	1. Total Glycosides of Paeoniae Alba capsule (2c, b.i.d.) 2. Immunosuppressant (compound amino peptide tablets 5t, b.i.d.)	1. Immunosuppressant (compound amino peptide tablets 5t, b.i.d.)	5.4 y (0.5 m–32 y)	5.6 y (1 m–40 y)	1. PASI 60 (*p* < 0.05)	8 w	Trial: No AEs Control No AEs
Song 2013 [90]	Randomized; Single center; Parallel	NR	43 (25/18) 34.5 ± 6.2 y	43 (26/17) 33.9 ± 6.0 y	1. Xiaoyin granule (3.5 g, t.i.d.) 2. Acitretin capsule (10 mg, t.i.d.)	1. Acitretin capsule (10 mg, t.i.d.)	8.1 ± 2.3 y	7.8 ± 2.4 y	1. PASI 60 (*p* < 0.05)	12 w	Trial: No AEs Control No AEs
Zhang 2013 [210]	Randomized; Single center; Parallel	NR	38 (20/18) 37.9 ± 5.9 y	38 (23/15) 33.9 ± 6.3 y	1. Xiaoyin capsule (5c, t.i.d.) 2. Acitretin capsule (10 mg, t.i.d.)	1. Acitretin capsule (10 mg, t.i.d.)	8.1 ± 2.7 y	7.8 ± 2.4 y	1. TNF-alpha (*p* < 0.01) 2. IL-8 (*p* < 0.01)	12 w	Trial: No AEs Control No AEs
Cheng 2014 [126]	Randomized; Single center; Parallel	NR	30 (17/13) 18–60 y	30 (16/14) 15–58 y	1. Yanghe decoction (b.i.d.) 2. Acitretin capsule (10 mg, b.i.d.)	1. Acitretin capsule (10 mg, b.i.d.)	1 w–5 y	2 w–6 y	1. PASI 60 (*p* < 0.05)	8 w	Detailed information NR
Du 2014 [125]	Randomized; Single center; Parallel	NR	70 (45/25) 39.6 ± 0.4 y	70 (45/25) 38.9 ± 0.5 y	1. Xiaoyin granule (3.5 g, t.i.d.) 2. Acitretin capsule (30 mg, q.d.)	1. Acitretin capsule (30 mg, q.d.)	6.3 ± 0.4 y	6.2 ± 0.4 y	1. PASI 60 (*p* < 0.05)	12 w	NR
Li 2014 [179]	Randomized; Single center; Parallel	NR	24 (14/10) 43.6 ± 10.78 y	24 (11/13) 45.3 ± 11.32 y	1. Huanglianjiedu decoction (200 mL, t.i.d.) 2. Methotrexate (2.5–5.0 mg, b.i.d.)	1. Methotrexate (2.5–5.0 mg, b.i.d.)	7.96 ± 4.41 y	7.49 ± 4.03 y	1. PASI 70 (*p* < 0.05)	4 w	Trial: 2 AEs Control: 5 AEs Including nausea, anorexia, hepatic dysfunction
Liang 2014 [172]	Randomized; Single center; Parallel	NR	39 (20/19) 38.43 ± 4.12 y	39 (19/20) 38.48 ± 4.15 y	1. Qinzhuliangxue feng (200 mL, b.i.d.) 2. Acitretin capsule (10 mg, b.i.d.)	1. Acitretin capsule (10 mg, b.i.d.)	5.33 ± 1.05 y	5.38 ± 1.03 y	1. PASI 60 (*p* < 0.05) 2. PASI score (*p* < 0.05) 3. Recurrence rate (*p* < 0.05)	60 d	Trial: No AEs Control: 6 AEs (1 hepatic and renal dysfunction, 2 pruritus, 3 tinnitus)
Liu 2014 [115]	Randomized; Single center; Parallel	NR	47 (29/18) 28.9 ± 10.4 y	49 (26/21) 31.2 ± 12.6 y	1. Runzaozhiyang capsule (2 g, t.i.d) 2. Topical corticosteroid (compound flumetasone ointment, b.i.d.)	1. Topical corticosteroid (compound flumetasone ointment, b.i.d.)	2.3 ± 0.84 y	2.7 ± 0.91 y	1. PASI 60 (*p* < 0.05)	4 w	Trial: 3 AEs (2 mild abdominal discomfort, 1 mild burning sensation) Control: 1 AE (1 mild stabbing)
Qiu 2014 [99]	Randomized; Single center; Parallel	NR	49 (29/20) 43.7 ± 6.9 y	33 (22/11) 46.7 ± 7.1 y	1. EAHM prescription (q.d.) 2. Acitretin capsule (20–50 mg, q.d.)	1. Acitretin capsule (20–50 mg, q.d.)	9.5 ± 3.1 y	9.1 ± 3.9 y	1. PASI 60 (*p* < 0.05)	12 w	Trial: No AEs Control: 26 AEs (1 hypokalemia, 6 hyperlipidemia, 19 xeroderma and xerostomia)
Zhang 2014a [144]	Randomized; Single center; Parallel	NR	38 (26/12) 38.62 ± 6.11 y	38 (24/14) 36.74 ± 5.23 y	1. Yinxie capsule (3c, t.i.d.) 2. Acitretin capsule (20 mg, q.d.)	1. Acitretin capsule (20 mg, q.d.)	2–78 m	1–81 m	1. PASI 60 (*p* < 0.05)	8 w	Trial 15 AEs Control 16 AEs Including pruritus, xeroma, epistaxis, xerostomia, dry lib
Zhang 2014b [209]	Randomized; Single center; Parallel	NR	40 (26/14) 35.40 ± 2.83 y	30 (19/11) 34.69 ± 3.46 y	1. Ziyinyangxuequfeng decoction (200 mL, b.i.d.) 2. Acitretin capsule (0.75 mg/kg/day, b.i.d.)	1. Acitretin capsule (0.75 mg/kg/day, b.i.d.)	6.35 ± 0.74 y	6.27 ± 0.68 y	1. PASI 60 (*p* < 0.05)	8 w	Trial: No AEs Control: No AEs
Zhang 2014c [196]	Randomized; Single center; Parallel	NR	36 (21/15) 33.5 y	36 (19/17) 35.3 y	1. Qingfeiliangxue decoction (200 mL, b.i.d.) 2. Acitretin capsule (0.5 mg/kg/day, b.i.d.)	1. Acitretin capsule (0.5 mg/kg/day, b.i.d.)	1–28 y	1–26 y	1. PASI 60 (*p* < 0.05) 2. PASI score (*p* < 0.05)	2 w	NR
Cai 2015 [189]	Randomized; Single center; Parallel	NR	130 (72/58) 38.5 ± 12.3 y	130 (67/63) 41.8 ± 11.9 y	1. Xiaoyin feng (200 mL, b.i.d.) 2. Topical corticosteroid (Calcipotriol ointment, b.i.d.)	1. Topical corticosteroid (Calcipotriol ointment, b.i.d.)	4.83 ± 1.39 y	4.95 ± 1.12 y	1. PASI 70 (*p* < 0.05) 2. PASI score (*p* < 0.05)	12 w	Trial: 14 AEs (4 hematuria, 10 drug eruption) Control: 16 AEs (5 hematuria, 11 drug eruption)
Jin 2015a [132]	Randomized; Single center; Parallel	NR	50 (28/22) 44.38 ± 2.9 y	50 (30/20) 43.3 ± 2.5 y	1. Matrine capsule (2t, t.i.d.) 2. Acitretin capsule (b.i.d.)	1. Acitretin capsule (b.i.d.)	22–68 y	20–65 y	1. PASI 60 (*p* < 0.05)	8 w	Trial: 4 AEs (2 pruritus, 1 hyperlipidemia, 1 hepatic dysfunction) Control: 4 AEs (2 pruritus, 2 hyperlipidemia)
Jin 2015b [194]	Randomized; Single center; Parallel	Simple randomization (random number table)	43 (24/19) 41.33 ± 14.19 y	41 (26/15) 37.17 ± 11.30 y	1. Qinzhuliangxue feng (200 mL, b.i.d.) 2. Acitretin capsule (10 mg, b.i.d.)	1. Acitretin capsule (10 mg, b.i.d.)	5.83 ± 1.60 y	7.16 ± 0.75 y	1. PASI 60 (*p* < 0.01) 2. PASI score (*p* < 0.01)	8 w	Trial: 21 AEs (18 xeroderma, 1 pruritus, 2 hepatic dysfunction) Control 42 AEs (27 xeroderma, 11 pruritus, 4 hepatic dysfuction)
Lu 2015a [102]	Randomized; Single center; Parallel	NR	Both group 62 (36/26) 29.5 ± 3.5 y Trial: 31	Both group 62 (36/26) 29.5 ± 3.5 y Control: 31	1. Qingying decoction (b.i.d.) 2. Acitretin capsule (30 mg, q.d.)	1. Acitretin capsule (30 mg, q.d.)	Both group 4.5 ± 1.2 y	Both group 4.5 ± 1.2 y	1. PASI 60 (*p* < 0.05) 2. PASI score (*p* < 0.05)	2 w	NR
Lu 2015b [118]	Randomized; Single center; Parallel	NR	54 (37/17) 21.2 ± 3.9 y	54 (33/21) 23.8 ± 2.1 y	1. Yangxierunfuyin (q.d.) 2. Immunosuppressant (compound amino peptide tablets 5t, b.i.d.)	1. Immunosuppressant (compound amino peptide tablets 5t, b.i.d.)	Both group 3 m–12 y	Both group 3 m–12 y	1. PASI score (*p* < 0.05)	16 w	Trial: 2 AEs (2 burning sensation) Control: 1 AEs (1 burning sensation)
Ma 2015 [188]	Randomized; Single center; Parallel	NR	55 (28/27) 43.7 ± 7.6 y	55 (29/26) 43.2 ± 7.4 y	1. Liangxuerunfu decoction (q.d.) 2. Topical boric acid (q.d.)	1. Topical boric acid (q.d.)	NR	NR	1. PASI 60 (*p* < 0.05)	12 w	NR
Peng 2015 [168]	Randomized; Single center; Parallel	NR	40 (27/13) 36.5 y (18–65 y)	40 (25/15) 34.3 y (19–65 y)	1. Yinxiping pill (q.d.) 2. Acitretin capsule (10 mg, t.i.d.)	1. Acitretin capsule (10 mg, t.i.d.)	3 m–26 y	1 m–30 y	1. PASI 60 (*p* < 0.05) 2. PASI score (*p* < 0.05) 3. Recurrence rate	12 w	Detailed information NR
Sun 2015 [156]	Randomized; Single center; Parallel	NR	40 (21/19) 31.4 ± 2.8 y	40 (20/20) 30.9 ± 2.7 y	1. Qingrexiaoyin decoction (250 mL, b.i.d.) 2. Topical corticosteroid (Calcipotriol ointment, b.i.d.)	1. Topical corticosteroid (Calcipotriol ointment, b.i.d.)	4.6 ± 2.7 y	4.8 ± 2.4 y	1. PASI 60 (*p* < 0.05) 2. TNF alpha (*p* < 0.05) 3. IL-8 (*p* < 0.05)	12 w	NR
Wang 2015a [185]	Randomized; Single center; Parallel	NR	57 (30/27) 7.1 ± 2.5 y	57 (27/30) 7.2 ± 2.4 y	1. Compound glycyrrhizin (25 mg, t.i.d.) 2. Acitretin capsule (0.5 mg/kg/day, b.i.d.)	1. Acitretin capsule (0.5 mg/kg/day, b.i.d.)	4.3 ± 1.4 m	4.4 ± 1.5 m	1. PASI 60 (*p* < 0.05)	12 w	Trial: 3 AEs (1 hyperlipidemia, 2 hair loss) Control: 10 AEs (3 hyperlipidemia, 5 hair loss, 2 hepatic dysfunction)
Wang 2015b [140]	Randomized; Single center; Parallel	Simple randomization (random number table)	Both group 59 (38/21) 37.29 ± 10.24 y Trial: 30	Both group 59 (38/21) 37.29 ± 10.24 y Control: 29	1. Researcher prescription (q.d.) 2. Acitretin capsule (20 mg, q.d.)	2. Acitretin capsule (20 mg, q.d.)	Both group 7.21 ± 2.13 y	Both group 7.21 ± 2.13 y	1. PASI 60 (*p* < 0.05) 2. Recurrence rate (*p* < 0.05)	4 w	Trial: No AEs Control: No AEs
Wang 2015c [150]	Randomized; Single center; Parallel	NR	30 (18/12) 22.92 ± 3.08 y	30 (16/14) 23.08 ± 2.92 y	1. Danggui-yinzi granule (t.i.d.) 2. Topical urea ointment (t.i.d.)	1. Topical urea ointment (t.i.d.)	2.02 ± 0.79 y	1.98 ± 0.66 y	1. PASI 70 (*p* < 0.05)	4 w	NR
Yuan 2015 [169]	Randomized; Single center; Parallel	NR	Both group 80 (35/45) 32.5 ± 4.1 y Trial: 40	Both group 80 (35/45) 32.5 ± 4.1 y Control: 40	1. Qingrexiaoyin decoction (250 mL, b.i.d.) 2. Topical corticosteroid (Calcipotriol ointment, b.i.d.)	1. Topical corticosteroid (Calcipotriol ointment, b.i.d.)	Both group 2.4 ± 0.7 y	Both group 2.4 ± 0.7 y	1. PASI 60 (*p* < 0.05)	12 w	NR
Zhang 2015a [199]	Randomized; Single center; Parallel	Simple randomization (random number table)	90 (58/32) 44.6 ± 3.8 y	90 (64/26) 43.8 ± 3.4 y	1. Liangxuerunfu decoction (200 mL, b.i.d.) 2. Acitretin capsule (10 mg, b.i.d.)	1. Acitretin capsule (10 mg, b.i.d.)	7.8 ± 0.5 y	8.7 ± 0.5 y	1. PASI 60 (*p* < 0.05)	8 w	Trial: 12 AEs (6 pruritus, 3 xerostomia, 2 nausea, 1 headache) Control 13 AEs (5 pruritus, 4 xerostomia, 3 nausea, 1 headache)
Zhang 2015b [214]	Randomized; Single center; Parallel	Simple randomization (random number table)	65 (36/29) 35.5 ± 9.7 y	65 (38/27) 26.76 ± 7.34 y	1. Yinxie capsule (4c, t.i.d.) 2. Topical Pyrithione Zinc aerosol (t.i.d.)	1. Topical Pyrithione Zinc aerosol (t.i.d.)	60.7 ± 21.3 m	64.6 ± 22.5 m	1. PASI 60 (*p* < 0.05) 2. VAS (*p* < 0.05) 3. DLQI (*p* < 0.05) 4. TNF alpha (*p* < 0.05) 5. IL-8 (*p* < 0.05)	8 w	NR
Zhang 2015c [206]	Randomized; Single center; Parallel	Simple randomization (random number table)	63 (38/25) 31.29 ± 0.04 y	65 (38/27) 29.22 y (19–43 y)	1. Zinyinqingrexiaofengsan (b.i.d.) 2. Acitretin capsule (20 mg, b.i.d.)	1. Acitretin capsule (20 mg, b.i.d.)	3 m–10 y	1–12 y	1. PASI 60 (*p* < 0.05)	8 w	Trial: 8 AEs (5 burning sensation, 2 erythema, 1 pruritus) Control: NR
Chen 2016 [96]	Randomized; Single center; Parallel	NR	35 (18/17) 37.5 ± 6.1 y	35 (19/16) 36.8 ± 6.0 y	1. Researcher prescription (b.i.d.) 2. Topical corticosteroid (Calcipotriol ointment, b.i.d.)	1. Topical corticosteroid (Calcipotriol ointment, b.i.d.)	9.2 ± 5.7 y	9.1 ± 5.4 y	1. PASI 70 (*p* < 0.05)	12 w	Trial: 0 AEs Control 0 AEs
He 2016a [110]	Randomized; Single center; Parallel	NR	Both group 90 (42/48) 40.1 ± 5.3 y Trial: 45	Both group 90 (42/48) 40.1 ± 5.3 y Control: 45	1. Liangxuerunfu decoction (b.i.d.) 2. Acitretin capsule (10 mg, b.i.d.)	1. Acitretin capsule (10 mg, b.i.d.)	Both group 11.8 ± 3.3 y	Both group 11.8 ± 3.3 y	1. PASI 60 (*p* < 0.05)	12 w	Trial: 4 AEs (4 gastrointestinal discomfort) Control: 12 AEs (3 gastrointestinal discomfort, 3 hyperlipdemia, 6 xerostomia)
He 2016b [93]	Randomized; Single center; Parallel	NR	34 (18/16) 39.21 ± 18.09 y	33 (17/16) 38.21 ± 17.68 y	1. Compound Qingdai pill (25 mg, t.i.d.) 2. Acitretin capsule (0.4 mg/kg/day, b.i.d.)	1. Acitretin capsule (0.4 mg/kg/day, b.i.d.)	4.85 ± 3.46 y	5.02 ± 3.96 y	1. PASI 60 (*p* < 0.05) 2. PASI score (*p* < 0.05)	8 w	Trial: 5 AEs (2 xerostomia, 2 hyperlipidemia, 1 gastrointestinal discomfort) Control: 4 AEs (3 xerostomia, 1 hyperlipidemia)
Jiang 2016 [131]	Randomized; Single center; Parallel	NR	40 (21/19) 34.62 ± 6.56 y	40 (23/17) 36.12 ± 5.44 y	1. Yinxiping pill (15 g, t.i.d.) 2. Topical corticosteroid (Calcipotriol ointment, b.i.d.)	1. Topical corticosteroid (Calcipotriol ointment, b.i.d.)	60.08 ± 41.03 m	59.45 ± 43.14 m	1. PASI 60 (*p* < 0.05) 2. PASI score (*p* < 0.05) 3. Recurrence rate (*p* < 0.05)	8 w	Trial: 5 AEs (2 burning sensation with skin rash, 1 gastrointestinal discomfort, 2 loose stool) Control: 3 AEs (burning sensation with skin rash)
Shan 2016 [154]	Randomized; Single center; Parallel	Simple randomization (random number table)	Both group 80 (45/35) 54.4 ± 10.4 y Trial: 40	Both group 80 (45/35) 54.4 ± 10.4 y Control: 40	1. Compound glycyrrhizin (50 mg, t.i.d.) 2. Acitretin capsule (50 mg, t.i.d.)	1. Acitretin capsule (50 mg, t.i.d.)	Both group 5.9 ± 3.2 y	Both group 5.9 ± 3.2 y	1. PASI 60 (*p* < 0.05) 2. PASI score (*p* < 0.05)	8 w	Trial: No AEs Control: 3 AEs (1 xerostomia, 1 xeroma, 1 xeroderma)
Wang 2016a [107]	Randomized; Single center; Parallel	NR	25 (16/9) 35.8 ± 7.6 y	25 (15/10) 37.1 ± 8.7 y	1. Xiaoyin granule (3.5 g, t.i.d.) 2. Acitretin capsule (10 mg, t.i.d.)	1. Acitretin capsule (10 mg, t.i.d.)	NR	NR	1. PASI 60 (*p* < 0.05)	16 w	NR
Wang 2016b [100]	Randomized; Single center; Parallel	NR	60 (35/25) 42.3 ± 6.9 y	60 (28/32) 39.5 ± 6.2 y	1. Piminxiao capsule (4c, t.i.d.) 2. Acitretin capsule (25 mg, q.d.)	1. Acitretin capsule (25 mg, q.d.)	NR	NR	1. PASI 60 (*p* < 0.05) 2. PASI score (*p* < 0.05)	8 w	NR
Wu 2016 [103]	Randomized; Single center; Parallel	NR	70 (43/27) 38.5 ± 2.6 y	70 (40/30) 39.1 ± 2.9 y	1. Xiaoyin granule (3.5 g, t.i.d.) 2. Acitretin capsule (25 mg, q.d.)	1. Acitretin capsule (25 mg, q.d.)	5.5 ± 1.4 y	5.9 ± 1.7 y	1. PASI 60 (*p* < 0.05)	8 w	Trial: 4 AEs Control: 13 AEs Including xerostomia, xeroderma, conjunctivitis, cheilitis
Xie 2016 [142]	Randomized; Single center; Parallel	Simple randomization (random number table)	52 (22/30) 39.1 ± 2.9 y	52 (24/28) 40.7 ± 9.5 y	1. Xiaoyin granule (3.5 g, t.i.d.) 2. Acitretin capsule (25–30 mg, q.d.)	1. Acitretin capsule (25–30 mg, q.d.)	5.32 ± 1.45 y	5.27 ± 1.42 y	1. PASI 60 (*p* < 0.05) 2. PASI score (*p* < 0.05) 3. Recurrence rate (*p* < 0.05) 4. IL-17 (*p* < 0.05)	4 w	Trial: 6 AEs (2 abdominal pain, 1 anorexia, 1 xerostomia, 1 dizziness, 1 conjunctivitis) Control: 5 AEs (1 pruritus, 1 tinnitus, 1 abdominal pain, 1 xeroma, 1 hepatic dysfunction)
Xu 2016 [195]	Randomized; Single center; Parallel	NR	Both group 114 (62/52) 40.5 ± 20.1 y Trial: 57	Both group 114 (62/52) 40.5 ± 20.1 y Control: 57	1. Compound glycyrrhizin (50 mg, t.i.d.) 2. Acitretin capsule (0.4 mg/kg/day, b.i.d.)	2. Acitretin capsule (0.4 mg/kg/day, b.i.d.)	NR	NR	1. PASI 60 (*p* < 0.05) 2. PASI score (*p* < 0.05)	6 w	Trial: 14 AEs Control: 30 AEs Including xeroderma, xeroma, xerostomia
Yang 2016a [178]	Randomized; Single center; Parallel	NR	40 (25/16) 39.33 ± 8.78 y	40 (30/10) 39.50 ± 9.37 y	1. Qinmei granule (b.i.d.) 2. Topical urea ointment (t.i.d.)	1. Topical urea ointment (t.i.d.)	NR	NR	1. PASI score (*p* < 0.05) 2. DLQI (*p* < 0.05)	12 w	Trial: 1 AE (1 Abnormal findings on urine test) Control: 1 AE (1 Abnormal findings on urine test)
Yang 2016b [104]	Randomized; Single center; Parallel	Simple randomization (random number generation)	23 (15/8) 30.5 y (25–54 y)	19 (12/7) 34.5 y (29–51 y)	1. Qingreliangxue decoction (b.i.d.) 2. Topical corticosteroid (Calcipotriol ointment, b.i.d.)	1. Topical corticosteroid (Calcipotriol ointment, b.i.d.)	7.62 m (3–26 m)	6.29 m (2–28 m)	1. PASI score (*p* < 0.05)	4 w	NR
Yu 2016 [201]	Randomized; Single center; Parallel	NR	Both group 40 (22/18) 40.3 y (18–74 y) Trial: 20	Both group 40 (22/18) 40.3 y (18–74 y) Control: 20	1. Qingreliangxue decoction (b.i.d.) 2. Topical corticosteroid (Calcipotriol ointment, b.i.d.) 3. Topical corticosteroid (Halometasone cream, b.i.d.)	1. Topical corticosteroid (Calcipotriol ointment, b.i.d.) 2. Topical corticosteroid (Halometasone cream, b.i.d.)	Both group 2.5 y (1 m–15 y)	Both group 2.5 y (1 m–15 y)	1. PASI score (*p* < 0.05)	8 w	NR
Cao 2017 [161]	Randomized; Single center; Parallel; single blind	Simple randomization (envelope concealment method)	30 (17/13) 36.02 ± 4.41 y	30 (18/12) 35.54 ± 4.36 y	1. Compound glycyrrhizin tablet (2t, t.i.d.) 2. Acitretin capsule (0.4 mg/kg/day, t.i.d.)	1. Acitretin capsule (0.4 mg/kg/day, t.i.d.)	4.66 ± 1.21 y	4.25 ± 1.02 y	1. PASI 60 (*p* < 0.05) 2. PASI score (*p* < 0.05)	8 w	Trial: 6 AEs (2 xeroderma, 3 xeroma, 1 hyperlipidemia) Control: 14 AEs (4 xeroderma, 5 xeroma, 3 hepatic dysfunction, 2 hyperlipidemia)
Cheng 2017 [94]	Randomized; Single center; Parallel	NR	27 (15/112) 38.4 ± 5.8 y	26 (15/11) 38.2 ± 5.3 y	1. Compound glycyrrhizin tablet (2–3t, t.i.d.) 2. Acitretin capsule (0.4 mg/kg/day, t.i.d.)	1. Acitretin capsule (0.4 mg/kg/day, t.i.d.)	5.6 ± 2.4 y	5.7 ± 2.5 y	1. PASI 60 (*p* < 0.05) 2. PASI score (*p* < 0.05)	8 w	Trial: 4AEs (4 xeroma, xerostomia, xeroderma) Control: 8 AEs (6 xeroma, xerostomia, xeroderma, 1 hair loss, 1 hyperlipidemia)
Ding 2017 [187]	Randomized; Single center; Parallel	Simple randomization (random number table)	40 (19/11) 36.15 ± 2.11 y	40 (22/18) 36.20 ± 2.07 y	1. Ziyinhuoxuerunzao decoction (200 mL, b.i.d.) 2. Acitretin capsule (20–50 mg, q.d.)	1. Acitretin capsule (20–50 mg, q.d.)	11.36 ± 1.00 y	11.41 ± 0.97	1. PASI 60 (*p* < 0.05) 2. PASI score (*p* < 0.05)	8 w	Trial: 1 AEs (1 focal pruritus with rash) Control: 2 AEs (2 focal pruritus with rash)
Du 2017 [114]	Randomized; Single center; Parallel	NR	32 (18/14) 32.2 ± 5.4 y	32 (21/11) 37.3 ± 5.2 y	1. Total Glycosides of Paeoniae Alba capsule (0.6 g, b.i.d.) 2. Topical corticosteroid (calcipotriol betamethasone ointment, q.d.)	1. Topical corticosteroid (calcipotriol betamethasone ointment, q.d.)	NR	NR	1. PASI 60 (*p* < 0.05)	4 w	Trial: 4 AEs (2 skin rash, 1 burning sensation, 1 folliculitis Control: No AE
Feng 2017 [130]	Randomized; Single center; Parallel	Simple randomization (random number table)	35 (21/14) 38.3 ± 4.1 y	35 (19/16) 35.7 ± 6.4 y	1. Yinxiping pill (15 g, t.i.d.) 2. Topical corticosteroid (Compound Flumetasone Ointment, b.i.d.)	1. Topical corticosteroid (Compound Flumetasone Ointment, b.i.d.)	5.9 ± 3.7 y	6.2 ± 3.3 y	1. PASI 60 (*p* < 0.05) 2. PASI score (*p* < 0.05)	8 w	Trial: 2 AEs (1 mild diarrhea, 1 mild skin rash) Control: 1 AEs (1 mild skin rash with pruritus)
Han 2017a [123]	Randomized; Single center; Parallel	NR	44 (25/19) 36.04 ± 7.15 y	44 (26/18) 35.69 ± 6.49 y	1. Xiaoyin granule (3.5 g, t.i.d.) 2. Acitretin capsule (30 mg, q.d.)	1. Acitretin capsule (30 mg, q.d.)	5.39 ± 2.48	5.21 ± 2.36	1. PASI 60 (*p* < 0.05) 2. Recurrence rate (*p* < 0.05) 3. IL-17 (*p* < 0.05)	12 w	Trial: 3 AEs (detailed information NR) Control 12 AEs (detailed information NR)
Han 2017b [162]	Randomized; Single center; Parallel	NR	48 (26/22) 45.72 ± 5.78 y	44 (27/21) 43.56 ± 4.43 y	1. Compound Qingdai capsule (4c, t.i.d.) 2. Acitretin capsule (20 mg, b.i.d.)	1. Acitretin capsule (20 mg, b.i.d.)	3.5 y (2 m–40 y)	4.2 y (3 m–36 y)	1. PASI 60 (*p* < 0.05) 2. PASI score (*p* < 0.05) 3. Recurrence rate (*p* < 0.05)	8 w	Detailed information NR
Li 2017 [203]	Randomized; Single center; Parallel	NR	30 (18/12) 36.4 ± 10.0 y	30 (15/15) 34.2 ± 12.7 y	1. Qingrejiedu decoction (6 g, b.i.d.) 2. Topical retinoid cream (b.i.d.)	1. Topical retinoid cream (b.i.d.)	5.73 ± 3.78 y	NR	1. PASI 60 (*p* < 0.05) 2. PASI score (*p* < 0.05) 3. TNF alpha (*p* < 0.05)	4 w	Trial: 3 AEs (3 diarrhea) Control: No AE
Liu 2017 [116]	Randomized; Single center; Parallel	NR	48 (27/21) 33.5 ± 6.5 y	52 (29/23) 33.8 ± 6.2 y	1. Yinxie capsule (4c, t.i.d.) 2. Acitretin capsule (20 mg, b.i.d.)	1. Acitretin capsule (20 mg, b.i.d.)	5.6 ± 7.2 y	5.2 ± 6.9 y	1. PASI 60 (*p* < 0.05) 2. PASI score (*p* < 0.05)	8 w	Trial: 65 AEs (13 xeroma, 16 xeroderma, 2 epistaxis, 14 folliculitis, 1 hepatic dysfunction, 19 hyperlipidemia) Control: 178 AEs (42 xeroma, 40 xeroderma, 9 epistaxis, 42 folliculitis, 8 hepatic dysfunction, 37 hyperlipidemia)
Luo 2017 [160]	Randomized; Single center; Parallel	NR	39 (26/13) 31.4 ± 2.3 y	36 (25/11) 32.7 ± 2.8 y	1. Compound glycyrrhizin (2–3t, t.i.d.) 2. Acitretin capsule (25–30 mg, t.i.d.)	1. Acitretin capsule (25–30 mg, t.i.d.)	NR	NR	1. PASI 60 (*p* < 0.05)	8 w	Trial: 5 AEs (2 pruritus, 1 xeroderma, 1 xerostomia, 1 xeroma) Control: 8 AEs (3 pruritus, 2 xeroderma, 2 xerostomia, 1 xeroma)
Pang 2017 [170]	Randomized; Single center; Parallel	NR	45 (22/23) 36.48 ± 14.21 y	45 (24/21) 37.02 ± 44.47 y	1. Compound Qingdai capsule (4c, t.i.d.) 2. Immunosuppressant (compound amino peptide tablets 5t, t.i.d.)	1. Immunosuppressant (compound amino peptide tablets 5t, t.i.d.)	47.68 ± 18.22 m	49.13 ± 18.80 m	1. PASI 70 (*p* < 0.05) 2. PASI score (*p* < 0.01) 3. DLQI (*p* < 0.05) 4. IL-8 (*p* < 0.05) 5. IFN gamma (*p* < 0.01)	8 w	NR
Shi 2017 [89]	Randomized; Single center; Parallel	NR	23 (12/11) 45.74 ± 8.43 y	23 (13/10) 45.72 ± 8.45 y	1. Liangxuexiaofeng decoction (200 mL, b.i.d.) 2. Acitretin capsule (30 mg, q.d.)	1. Acitretin capsule (30 mg, q.d.)	7.29 ± 1.25 y	7.28 ± 1.28 y	1. PASI 60 (*p* < 0.05) 2. PASI score (*p* < 0.01) 3. IL-8 (*p* < 0.05)	8 w	Trial: 3 AEs (1 xerostomia, 1 xeroma, 1 xeroderma) Control: 2 AEs (1 xerostomia, 1 nausea)
Song 2017 [108]	Randomized; Single center; Parallel	NR	70 (32/28) 41.02 ± 5.39 y	70 (39/31) 40.76 ± 5.32 y	1. Yangxuetongluo decoction (b.i.d.) 2. Acitretin capsule (10 mg, t.i.d.)	1. Acitretin capsule (10 mg, t.i.d.)	6.41 ± 1.00 y	6.49 ± 1.03 y	1. PASI 70 (*p* < 0.05) 2. VAS (*p* < 0.05) 3. DLQI (*p* < 0.05) 4. TNF alpha (*p* < 0.05)	8 w	Trial: 9 AEs (2 conjunctivitis, 4 xerostomia, 3 headache) Control: 8 AEs (3 conjunctivitis, 2 xerostomia, 2 headache, 1 muscular pain)
Wang 2017 [159]	Randomized; Single center; Parallel	NR	60 (32/28) 32.46 ± 6.25 y	60 (32/28) 33.08 ± 6.32 y	1. Yinxie capsule (4c, t.i.d.) 2. Acitretin capsule (20 mg, t.i.d.)	1. Acitretin capsule (20 mg, t.i.d.)	5.61 ± 7.32 y	5.29 ± 6.96 y	1. PASI 60 (*p* < 0.05) 2. PASI score (*p* < 0.05) 3. IL-8 (*p* < 0.05)	8 w	Trial: 54 AEs (15 xeroma, 18 xeroderma, 3 epistaxis, 16 folliculitis, 2 hepatic dysfunction, 15 hyperlipidemia) Control: 180 AEs (48 xeroma, 43 xeroderma, 11 epistaxis, 32 folliculitis, 9 hepatic dysfunction, 37 hyperlipidemia)
Wu 2017 [173]	Randomized; Single center; Parallel	NR	40 (26/14) 40.85 ± 15.48 y	40 (25/15) 41.64 ± 15.86 y	1. Shentongzhuyu decoction (200 mL, b.i.d.) 2. Methotrexate (10 mg, q.w.) 3. Sulfasalazine tablets (1.0 g, t.i.d.) 4. Diclofenac sodium extended-release tablet (0.1 g, q.d.) 5. Folic acid tablet (10 mg, q.d.)	1. Methotrexate (10 mg, q.w.) 2. Sulfasalazine tablets (1.0 g, t.i.d.) 3. Diclofenac sodium extended-release tablet (0.1 g, q.d.) 4. Folic acid tablet (10 mg, q.d.)	NR	NR	1. PASI 70 (*p* < 0.01) 2. PASI score (*p* < 0.05)	12 w	Trial: 9 AEs (2 leukopenia, 3 hepatic dysfunction, 1 hyperbilirubinemia, 3 nausea and vomiting) Control 10 AEs (3 leukopenia, 2 hepatic dysfunction, 1 hyperbilirubinemia, 4 nausea and vomiting)
Yang 2017 [213]	Randomized; Single center; Parallel	Simple randomization	40 (25/15) 30.6 ± 8.21 y	40 (24/16) 32.5 ± 7.10 y	1. Xiaoyin granule (3.5 g, t.i.d.) 2. Acitretin capsule (20 mg, q.d.)	1. Acitretin capsule (20 mg, q.d.)	4.51 ± 3.13 y	4.60 ± 3.02 y	1. PASI 60 (*p* < 0.01) 2. PASI score (*p* < 0.05) 3. TNF alpha (*p* < 0.05) 4. IL-17 (*p* < 0.05) 5. IL-23 (*p* < 0.05)	12 w	Trial: 9AEs (3 pruritus, 5 xerostomia, 1 xeroma) Control: 17 AEs (7 pruritus, 8 xerostomia, 1 dizzines, 1 xeroma)
Zeng 2017 [190]	Randomized; Single center; Parallel	NR	31 (21/11) 35.14 ± 0.15 y	31 (21/10) 35.29 ± 0.18 y	1. Compound glycyrrhizin capsule (2-3c, t.i.d.) 2. Acitretin capsule (10 mg, t.i.d.)	1. Acitretin capsule (10 mg, t.i.d.)	32.14 ± 1.25 m	33.45 ± 1.34 m	1. PASI 60 (*p* < 0.05)	8 w	Trial: 13 AEs (2 xerostomia, 3 xeroma, 2 pruritus, 4 nausea, 2 dizziness) Control: 8 AEs (1 xerostomia, 2 xeroma, 1 pruritus, 3 nausea, 1 dizziness)
Zhang 2017a [113]	Randomized; Single center; Parallel	NR	52 (27/25) 28.5 ± 5.2 y	52 (26/26) 30.1 ± 4.1 y	1. Taohongershao decoction (150 mL, t.i.d.) 2. Acitretin capsule (10 mg, t.i.d.)	1. Acitretin capsule (10 mg, t.i.d.)	7.3 ± 4.5 y	8.5 ± 4.9 y	1. PASI score (*p* < 0.05) 2. Recurrence rate (*p* < 0.05)	4 w	NR
Zhang 2017b [112]	Randomized; Single center; Parallel	NR	45 (21/24) 27.5 ± 7.4 y	45 (22/23) 28.0 ± 9.5 y	1. Taohongershao decoction (150 mL, t.i.d.) 2. Acitretin capsule (10 mg, t.i.d.)	1. Acitretin capsule (10 mg, t.i.d.)	10.5 ± 8.3 y	10.9 ± 8.0 y	1. PASI 60 (*p* < 0.05) 2. DLQI (*p* < 0.05) 3. IFN gamma (*p* < 0.05) 4. IL-17 (*p* < 0.05) 5. IL-23 (*p* < 0.05)	4 w	NR
Zhang 2017c [105]	Randomized; Single center; Parallel	NR	50 (23/27) 32.5 ± 5.9 y	50 (25/25) 30.9 ± 6.1 y	1. Taohongershao decoction (150 mL, t.i.d.) 2. Acitretin capsule (10 mg, t.i.d.)	1. Acitretin capsule (10 mg, t.i.d.)	12.6 ± 7.5 y	11.5 ± 6.9 y	1. PASI 60 (*p* < 0.01) 2. PASI score (*p* < 0.05)	8 w	NR
Zhang 2017d [121]	Randomized; Single center; Parallel	NR	17 (10/7) 46.47 ± 14.06	17 (9/8) 46.41 ± 18.45	1. Liangxuexiao feng (6 g, b.i.d.) 2. Acitretin capsule (10 mg, b.i.d.)	1. Acitretin capsule (10 mg, b.i.d.)	16.29 ± 10.49 y	16.08 ± 12.80 y	1. PASI 60 (*p* < 0.05) 2. PASI score (*p* < 0.05)	8 w	NR
Zhang 2017e [200]	Randomized; Single center; Parallel	NR	55 (24/31) 30.1 ± 4.4 y	55 (22/33) 29.8 ± 7.3 y	1. Taohongershao decoction (150 mL, t.i.d.) 2. Acitretin capsule (10 mg, t.i.d.)	1. Acitretin capsule (10 mg, t.i.d.)	7.5 ± 6.3 y	8.1 ± 6.4 y	1. PASI 60 (*p* < 0.05) 2. PASI score (*p* < 0.05) 3. IFN gamma (*p* < 0.05)	4 w	NR
Zhao 2017 [128]	Randomized; Single center; Parallel	NR	40 (23/17) 36.35 ± 2.09 y	40 (22/18) 36.25 ± 2.13 y	1. Yinxie capsule (3c, t.i.d.) 2. Acitretin capsule (20 mg, q.d.).	1. Acitretin capsule (20 mg, q.d.).	NR	NR	1. PASI 60 (*p* < 0.05)	8 w	Trial: 18 AEs (6 pruritus, 7 epistaxis, 5 xerostomia) Control: 19 AEs (7 pruritus, 7 epistaxis, 6 xerostomia)
Chai 2018 [158]	Randomized; Single center; Parallel	NR	46 (26/20) 45.3 ± 3.8 y	46 (25/21) 45.5 ± 3.6 y	1. Xiaoyin decoction (b.i.d.) 2. Topical corticosteroid (Calcipotriol ointment, b.i.d.)	1. Topical corticosteroid (Calcipotriol ointment, b.i.d.)	2.3 ± 0.5 y	2.1 ± 0.4 y	1. PASI 60 (*p* < 0.05) 2. IFN gamma (*p* < 0.05) 3. IL-8 (*p* < 0.05)	8 w	Trial: 2 AEs (1 skin rash, 1 mild gastrointestinal discomfort) Control: 3 AEs (2 skin rash, 1 pruritus)
Li 2018 [109]	Randomized; Single center; Parallel	Simple randomization (random number table)	44 (23/21) 35.01 ± 7.09 y	44 (24/20) 35.31 ± 7.29 y	1. Yinxie capsule (4c, t.i.d.) 2. Acitretin capsule (20 mg, b.i.d.)	1. Acitretin capsule (20 mg, b.i.d.).	7.69 ± 3.69 y	7.71 ± 3.46 y	1. PASI 60 (*p* < 0.05) 2. PASI score (*p* < 0.01)	8 w	Trial: 1 AEs (1 nausea) Control: 3 AEs (1 headache, 2 nausea)
Liu 2018 [147]	Randomized; Single center; Parallel Three arm trial	NR	25 (11/14) 37.21 ± 9.87 y	25 (13/12) 39.42 ± 9.23 y	1. Banzhilian decoction (200 mL, b.i.d.) 2. Topical corticosteroid (Calcipotriol ointment, b.i.d.).	1. Topical corticosteroid (Calcipotriol ointment, b.i.d.)	6.8 ± 5.1 y	6.2 ± 3.9 y	1. PASI 60 (*p* < 0.05) 2. PASI score (*p* < 0.05)	8 w	NR
Luo 2018 [212]	Randomized; Single center; Parallel	NR	50 (31/19) 32.46 ± 10.24 y	50 (29/21) 33.57 ± 10.82 y	1. Zicaohuoxue decoction (200 mL, bi.d.) 2. Acitretin capsule (30 mg, q.d.)	1. Acitretin capsule (30 mg, q.d.)	2.46 ± 1.24 y	2.52 ± 1.28 y	1. PASI 60 (*p* < 0.05) 2. TNF alpha (*p* < 0.05) 3. DLQI (*p* < 0.05)	8 w	Trial: 8 AEs (1 pruritus, 2 xeroma, 2 headache, 3 nausea) Control: 10 AEs (1 pruritus, 3 xeroma, 3 headache, 3 nausea)
Ma 2018a [192]	Randomized; Single center; Parallel	Simple randomization (random number table)	37 (21/16) 38.1 ± 4.2 y	42 (23/19) 37.4 ± 4.1 y	1. Xiaoyin granule (3.5 g, t.i.d.) 2. Topical corticosteroid (Calcipotriol ointment, b.i.d.) 3. Acitretin capsule (30 mg, q.d.)	1. Topical corticosteroid (Calcipotriol ointment, b.i.d.) 2. Acitretin capsule (30 mg, q.d.)	3.66 ± 1.01 y	3.7 ± 1.3 y	1. PASI 60 (*p* < 0.05)	4 w	NR
Ma 2018b [166]	Randomized; Single center; Parallel	NR	34 (19/15) 40.2 ± 6.9 y	34 (21/13) 41.6 ± 7.5 y	1. Qingrequshi decoction (b.i.d.) 2. Methotrexate (15 mg, q.w.)	1. Methotrexate (15 mg, q.w.)	8.7 ± 2.6 y	8.2 ± 2.4 y	1. PASI score (*p* < 0.05)	12 w	NR
Xiao 2018 [176]	Randomized; Single center; Parallel	Simple randomization (random number table)	21 (11/10) 27.5 ± 2.2 y	21 (15/6) 27.3 ± 1.2 y	1. Xiaoyin decoction (200 mL, b.i.d.) 2. Topical corticosteroid (Calcipotriol ointment, b.i.d.)	1. Topical corticosteroid (Calcipotriol ointment, b.i.d.)	NR	NR	1. TNF alpha (*p* < 0.05) 2. IL-17 (*p* < 0.05) 3. IL-22 (*p* < 0.05)	12 w	NR
Xie 2018 [106]	Randomized; Single center; Parallel	NR	60 (34/26) 36.5 y (19–65 y)	60 (29/31) 34.3 y (19–64 y)	1. Qingying decoction (b.i.d.) 2. Acitretin capsule (30 mg, q.d.)	1. Acitretin capsule (30 mg, q.d.)	6.4 y (3 m–26 y)	6.1 y (1 m–30 y)	1. PASI 60 (*p* < 0.05) 2. PASI score (*p* < 0.05)	8 w	Detailed information NR
Zhang 2018a [111]	Randomized; Single center; Parallel	NR	48 (25/23) 35.43 ± 0.16	48 (28/20) 35.28 ± 0.23	1. Compound glycyrrhizin (2–3t, t.i.d.) 2. Acitretin capsule (10 mg, t.i.d.)	1. Acitretin capsule (10 mg, t.i.d.)	33.12 ± 1.64 m	32.65 ± 1.14 m	1. PASI score (*p* < 0.05)	8 w	NR
Zhang 2018b [174]	Randomized; Single center; Parallel	Simple randomization	36 (20/16) 29.15 ± 6.24 y	36 (19/17) 29.36 ± 6.02	1. Xiaoyin granule (3.5 g, t.i.d.) 2. Topical corticosteroid (Calcipotriol ointment, b.i.d.)	1. Topical corticosteroid (Calcipotriol ointment, b.i.d.)	5.2 ± 1.3 y	5.4 ± 1.2 y	1. PASI 60 (*p* < 0.05) 2. PASI score (*p* < 0.05)	8 w	NR
Zhou 2018 [139]	Randomized; Single center; Parallel	NR	39 (17/22) 38.82 ± 1.29 y	39 (19/20) 38.71 ± 1.22 y	1. Keyin pills (10 mg, b.i.d.) 2. Acitretin capsule (10 mg, t.i.d.)	1. Acitretin capsule (10 mg, t.i.d.)	7.59 ± 0.78 y	7.46 ± 0.65 y	1. PASI 60 (*p* < 0.05) 2. PASI score (*p* < 0.05) 3. IFN gamma (*p* < 0.05) 4. IL-17 (*p* < 0.05)	8 w	Trial: 8 AEs (1 conjunctivitis, 1 skin rash, 2 arthralgia, 1 headache, 3 nausea and vomiting) Control: 11 AEs (1 conjunctivitis, 2 skin rash, 3 arthralgia, 1 headache, 4 nausea and vomiting)
Chen 2019 [198]	Randomized; Single center; Parallel	NR	62 (26/36) 37.62 ± 6.34 y	62 (28/34) 35.74 ± 5.54 y	1. Compound glycyrrhizin tablet (2–3t, t.i.d.) 2. Topical corticosteroid (Calcipotriol ointment, b.i.d.) 3. Acitretin capsule (25–30 mg, q.d.)	1. Topical corticosteroid (Calcipotriol ointment, b.i.d.) 2. Acitretin capsule (25–30 mg, q.d.)	6.92 ± 3.05 y	6.25 ± 2.47 y	1. PASI score (*p* < 0.05)	8 w	NR
Ge 2019 [145]	Randomized; Single center; Parallel	NR	40 (21/19) 27 ± 2.4 y	40 (22/18) 26 ± 2.3 y	1. Compound glycyrrhizin (50 mg, t.i.d.) 2. Acitretin capsule (20 mg, t.i.d.)	1. Acitretin capsule (20 mg, t.i.d.)	3 m–31 y	3 m–31.5 y	1. PASI 60 (*p* < 0.05) 2. PASI score (*p* < 0.05)	8 w	Trial: 5 AEs (including xerostomia, xeroma, xeroderma) Control: 8 AEs (including xerostomia, xeroma, xeroderma, 2 hair loss, 1 hyperlipidemia)
Han 2019 [97]	Randomized; Single center; Parallel	NR	Both group 80 (59/21) 37.37 ± 9.48 y	Both group 80 (59/21) 37.37 ± 9.48 y	1. Qinzhuliangxue feng (b.i.d.) 2. Acitretin capsule (10 mg, b.i.d.)	1. Acitretin capsule (10 mg, b.i.d.)	Both group 5.29 ± 1.44 y	Both group 5.29 ± 1.44 y	1. PASI 60 (*p* < 0.05) 2. PASI score (*p* < 0.05)	4 w	NR
Hu 2019 [119]	Randomized; Single center; Parallel	Simple randomization	62 (34/28) 36.8 ± 8.1 y	59 (33/26) 37.8 ± 9.4 y	1. Liangxuexiaobi decoction (150 mL, b.i.d.) 2. Topical corticosteroid (Calcipotriol betamethasone ointment, q.d.)	1. Topical corticosteroid (Calcipotriol betamethasone ointment, q.d.)	45.4 ± 12.5 m	46.1 ± 11.1 m	1. PASI score (*p* < 0.05)	8 w	Trial: 2 AEs (2 loose stool) Control: 1AE (1 skin rash)
Lu 2019 [152]	Randomized; Single center; Parallel; single blind	Simple randomization (envelope concealment method)	30 (15/15) 21–66 y	30 (16/14) 22–65 y	1. Yangxuequfeng granule (b.i.d.) 2. Topical urea ointment (t.i.d.)	1. Topical urea ointment (t.i.d.)	NR	NR	1. PASI 70 (*p* < 0.05) 2. DLQI (*p* < 0.05)	4 w	NR
Xun 2019 [134]	Randomized; Single center; Parallel	NR	52 (24/28) 40–50 y	52 (22/30) 40–51 y	1. Yinxie capsule (15 g, t.i.d.) 2. Acitretin capsule (25–50 mg, t.i.d.)	1. Acitretin capsule (25–50 mg, t.i.d.)	NR	NR	1. Recurrence rate (*p*-value NR)	4 w	NR
Yang 2019 [149]	Randomized; Single center; Parallel	NR	41 (24/17) 38.24 ± 4.19 y	41 (25/16) 38.57 ± 4.03 y	1. Compound Qingdai capsule (4c, t.i.d.) 2. Topical corticosteroid (Calcipotriol betamethasone ointment, q.d.)	1. Topical corticosteroid (Calcipotriol betamethasone ointment, q.d.)	14.39 ± 2.78 y	14.79 ± 1.93 y	1. PASI 60 (*p* < 0.05) 2. TNF alpha (*p* < 0.05) 3. IL-17 (*p* < 0.05) 4. IL-23 (*p* < 0.05)	8 w	Trial: 4 AEs (1 xeroderma, 1 pruritus, 1 hyperlipidemia, 1 hepatic dysfunction) Control: 6 AEs (1 skin scale with edema, 1 xeroderma, 2 pruritus, 1 hyperlipidemia, 1 hepatic dysfunction)
Yao 2019 [180]	Randomized; Single center; Parallel	Simple randomization (random number table)	52 (29/23) 45.37 ± 6.12 y	52 (27/25) 45.13 ± 6.08 y	1. Liangxuerunzao decoction (250 mL, b.i.d.) 2. Topical corticosteroid (Calcipotriol ointment, b.i.d.)	1. Topical corticosteroid (Calcipotriol ointment, b.i.d.)	9.77 ± 1.30 y	9.86 ± 1.35 y	1. PASI 70 (*p* < 0.05) 2. PASI score (*p* < 0.01) 3.IL-17 (*p* < 0.01) 4.IL-22 (*p* < 0.01) 5.IL-23 (*p* < 0.01)	8 w	NR
Zhong 2019 [122]	Randomized; Single center; Parallel	NR	46 (22/24) 36.72 ± 6.21 y	46 (23/23) 37.23 ± 5.78 y	1. Piminxiao capsule (4c, t.i.d.) 2. Acitretin capsule (30 mg, q.d.)	1. Acitretin capsule (30 mg, q.d.)	5.10 ± 1.76 y	5.25 ± 1.28 y	1. PASI 60 (*p* < 0.05) 3. IL-17 (*p* < 0.05)	12 w	NR
Chen 2020 [155]	Randomized; Single center; Parallel	NR	47 (25/22) 36.9 ± 5.3 y	47 (24/23) 38.2 ± 5.1 y	1. Compound glycyrrhizin capsule (3c, t.i.d.) 2. Acitretin capsule (25–30 mg, q.d.)	2. Acitretin capsule (25–30 mg, q.d.)	NR	NR	1. PASI 60 (*p* < 0.05) 2. PASI score (*p* < 0.05)	8 w	Trial: 7 AEs (1 nausea, 3 hyperesthesia, 2 xeroderma, 1 xerostomia) Control: 5 AEs (2 nausea, 1 hyperesthesia 1 xeroderma, 1 xerostomia)
Hao 2020 [120]	Randomized; Single center; Parallel	Simple randomization (random number table)	30 (22/8) 42 ± 13 y	30 (21/9) 41 ± 12 y	1. Liangxuexiaobi pill (6c, t.i.d.) 2. Topical corticosteroid (Calcipotriol ointment, b.i.d.)	1. Topical corticosteroid (Calcipotriol ointment, b.i.d.)	3.71 ± 3.26 y	4.28 ± 2.96 y	1. PASI 60 (*p* < 0.05) 2. PASI score (*p* < 0.05) 3. DLQI (*p* < 0.05)	4 w	Trial: 1 AE (1 skin rash) Control: 4 AEs (2 erythema, 1 skin rash with burning sensation, 1 pruritus)
Ji 2020 [204]	Randomized; Single center; Parallel	NR	25 (15/10) 37.8 y (25.3–49.2 y)	25 (17/8) 37.5 y (27.1–48.2 y)	1. Compound Qingdai capsule (6 g, t.i.d.) 2. Methotrexate (detailed dosage NR)	1. Methotrexate (detailed dosage NR)	NR	NR	1. PASI score (*p* < 0.05) 2. DLQI (*p* < 0.05)	8 w	NR
Liu 2020a [181]	Randomized; Single center; Parallel	NR	30 (18/12) 41.3 ± 5.1 y	30 (17/13) 42.3 ± 6.2 y	1. Compound glycyrrhizin tablet (2t, t.i.d.) 2. Acitretin capsule (10 mg, b.i.d.)	1. Acitretin capsule (10 mg, b.i.d.)	5.23 ± 1.13 y	5.31 ± 1.23 y	1. PASI score (*p* < 0.05) 2. DLQI (*p* < 0.05)	8 w	Trial: 5 AEs (2 xerostomia, 2 xeroma, 1 xeroderma) Control: 11 AEs (3 xerostomia, 3 xeroma, 2 xeroderma, 2 hair loss, 1 hyperlipidemia)
Liu 2020b [167]	Randomized; Single center; Parallel	NR	92 (46/46) 54.18 ± 4.19 y	92 (45/47) 54.81 ± 4.33 y	1. Xiaoyin decoction (6c, t.i.d.) 2. Topical corticosteroid (Calcipotriol ointment, b.i.d.)	1. Topical corticosteroid (Calcipotriol ointment, b.i.d.)	8.43 ± 2.28 y	8.79 ± 2.30 y	1. IL-8 (*p* < 0.05)	8 w	NR
Lu 2020 [95]	Randomized; Single center; Parallel	NR	56 (31/25) 44.58 ± 7.12 y	56 (29/27) 45.01 ± 6.93 y	1. Total Glycosides of Paeoniae Alba capsule (0.6 g, t.i.d.) 2. Acitretin capsule (25–30 mg, q.d.)	2. Acitretin capsule (25–30 mg, q.d.)	7.63 ± 1.83 y	7.79 ± 1.72 y	1. PASI 60 (*p* < 0.05) 2. PASI score (*p* < 0.05)	8 w	Detailed information NR
Qu 2020 [177]	Randomized; Single center; Parallel	Simple randomization (computer assisted random assignment)	46 (25/21) 40.44 ± 6.74 y	46 (26/20) 40.13 ± 6.48 y	1. Yinxie capsule (4c, t.i.d.) 2. Acitretin capsule (25–30 mg, q.d.)	2. Acitretin capsule (25–30 mg, q.d.)	7.75 ± 3.75 y	7.45 ± 3.46 y	1. PASI 60 (*p* < 0.05)	8 w	NR
Ren 2020 [151]	Randomized; Single center; Parallel	NR	51 (27/24) 38.19 ± 2.14 y	51 (26/25) 38.62 ± 2.37 y	1. Xiaoyin granule (10 g, t.i.d.) 2. Acitretin capsule (10 mg, t.i.d.)	2. Acitretin capsule (10 mg, t.i.d.)	2.25 ± 0.95 y	2.34 ± 0.83 y	1. PASI 60 (*p* < 0.05) 2. PASI score (*p* < 0.05)	12 w	Trial: 6 AEs (2 xerostomia, 1 pruritus, 2 gastrointestinal discomfort, 1 hepatic dysfunction) Control: 18 AEs (5 xerostomia, 6 pruritus, 4 gastrointestinal discomfort, 3 hepatic dysfunction)
Shen 2020 [133]	Randomized; Single center; Parallel	NR	31 (15/16) 36 ± 14 y	32 (16/16) 35 ± 11 y	1. Liangxuejiedu decoction (200 mL, b.i.d.) 2. Topical corticosteroid (Calcipotriol ointment, q.d.) 3. Topical corticosteroid (Mometasone furoate cream, q.d.)	1. Topical corticosteroid (Calcipotriol ointment, q.d.) 2. Topical corticosteroid (Mometasone furoate cream, q.d.)	6.67 ± 6.30 y	6.42 ± 6.13 y	1. PASI 60 (*p* < 0.05) 2. PASI score (*p* < 0.05) 3. DLQI (*p* < 0.05)	8 w	NR
Wu 2020 [193]	Randomized; Single center; Parallel	NR	50 (28/22) 35.4 ± 7.9 y	50 (27/23) 34.4 ± 7.6 y	1. Compound glycyrrhizin tablet (10 g, t.i.d.) 2. Acitretin capsule (10 mg, t.i.d.)	2. Acitretin capsule (10 mg, t.i.d.)	4.6 ± 3.1 y	4.5 ± 2.9 y	1. PASI 70 (*p* < 0.05)	8 w	Trial: 25 AEs (21 xerostomia, xeroma, xeroderma, 2 hepatic dysfunction, 2 hyperlipidemia) Control: 47 AEs (35 xerostomia, xeroma, xeroderma, 8 hepatic dysfunction, 4 hyperlipidemia)
Yang 2020 [101]	Randomized; Single center; Parallel	NR	78 (44/34) 39.24 ± 6.51 y	78 (45/33) 39.17 ± 6.48 y	1. Xiaoyin granule (3.5 g, t.i.d.) 2. Acitretin capsule (30 mg, q.d.)	1. Acitretin capsule (30 mg, q.d.)	7.71 ± 2.51 y	7.69 ± 2.53 y	1. PASI 60 (*p* < 0.05)	8 w	NR
Zheng 2020 [205]	Randomized; Single center; Parallel	NR	32 (16/16) 29.8 ± 6.2 y	32 (14/18) 30.5 ± 5.8 y	1. Jueyin granule (b.i.d.) 2. Acitretin capsule (20 mg, q.d.) 3. Topical corticosteroid (Compound flumetasone cream, b.i.d.)	1. Acitretin capsule (20 mg, q.d.) 2. Topical corticosteroid (Compound flumetasone cream, b.i.d.)	NR	NR	1. PASI 60 (*p* < 0.05) 2. PASI score (*p* < 0.05)	8 w	NR
Jin 2021 [148]	Randomized; Single center; Parallel	NR	50 (24/26) 42.06 ± 4.37 y	32 (14/18) 41.32 ± 4.93 y	1. Xiaoyin decoction (200 mL, b.i.d.) 2. Topical corticosteroid (Calcipotriol ointment, b.i.d.)	1. Topical corticosteroid (Calcipotriol ointment, b.i.d.)	3.47 ± 1.40 y	3.32 ± 1.03 y	1. TNF alpha (*p* < 0.05) 2. IL-17 (*p* < 0.05) 3. IL-22 (*p* < 0.05)	4 w	NR
Lan 2021 [117]	Randomized; Single center; Parallel	NR	40 (25/15) 46.21 ± 8.07 y	40 (23/17) 46.05 ± 8.69 y	1. Dangguiyinzi (150 mL, b.i.d.) 2. Acitretin capsule (10 mg, b.i.d.)	2. Acitretin capsule (10 mg, b.i.d.)	6.25 ± 3.00 y	6.18 ± 1.95 y	1. PASI 60 (*p* < 0.05) 2. PASI score (*p* < 0.05)	12 w	Trial: 5 AEs (1 hyperlipidemia, 2 xeroma, 2 xerostomia) Control: 21 AEs (7 hyperlipidemia, 4 xeroma, 8 xerostomia, 2 cheilitis)
Le 2021 [175]	Randomized; Single center; Parallel	NR	43 (24/19) 42.17 ± 2.75 y	42 (21/21) 42.34 ± 2.66 y	1. Tianxian decoction (t.i.d.) 2. Acitretin capsule (30 mg, q.d.)	2. Acitretin capsule (30 mg, q.d.)	4.68 ± 0.91 y	4.71 ± 0.88 y	1. PASI score (*p* < 0.05) 2. DLQI (*p* < 0.05)	12 w	NR
Tang 2021 [171]	Randomized; Single center; Parallel	Simple randomization (random number table)	36 (19/17) 34.17 ± 1.75 y	36 (20/16) 33.25 ± 1.67 y	1. Qingreyangxuejiedu decoction (200 mL, b.i.d.) 2. Topical corticosteroid (Calcipotriol betamethasone ointment, b.i.d.)	1. Topical corticosteroid (Calcipotriol betamethasone ointment, b.i.d.)	1.96 ± 0.76 y	1.75 ± 0.49 y	1.P ASI 60 (*p* < 0.05) 2. PASI score (*p* < 0.05) 3. IL-17 (*p* < 0.05) 4. IL-23 (*p* < 0.05)	8 w	Trial: 1 AE (1 gastrointestinal discomfort) Control: 2 AEs (1 telangiectasia, 1 folliculitis)
Wang 2021 [98]	Randomized; Single center; Parallel	NR	42 (22/20) 46.75 ± 21.23 y	41 (21/20) 45.91 ± 20.89 y	1. Qingreliangxue decoction (100 mL, b.i.d.) 2. Acitretin capsule (20 mg, q.d.) 3. Topical corticosteroid (Mometasone furoate cream, q.d.)	1. Acitretin capsule (20 mg, q.d.) 2. Topical corticosteroid (Mometasone furoate cream, q.d.)	12.74 ± 5.23 y	11.76 ± 5.48 y	1. PASI 60 (*p* < 0.05) 3. Recurrence rate (*p* < 0.05)	8 w	Trial: 9AEs (2 xerostomia, 5 xeroderma with pruritus, 2 diarrhea) Control: 10 AEs (2 xerostomia, 6 xeroderma with pruritus, 2 diarrhea)

AEs: adverse events; b.i.d: bis in die; c: capsules; d: days; DLQI: dermatology life quality index; g: grams; IFN: interferon; IL: interleukin; m: months; mg: milligrams; NR: not reported; p: packs; PASI: psoriasis area severity index; q.d: quaque die; SD: standard deviation; t: tablets; t.i.d: ter in die; TNF: tumor necrosis factor; w: weeks; y: years; μg: micrograms.

**Table 2:** Methodological quality of the included studies according to the risk of bias 2.0.

Author	Year	D1	D2	D3	D4	D5	Overall
Che	2004	Sc	Sc	L	H	Sc	H
Chen	2004	Sc	Sc	L	Sc	Sc	Sc
Xu	2005	Sc	Sc	L	Sc	Sc	Sc
Liu	2006	Sc	Sc	L	H	Sc	H
Chen	2007	Sc	Sc	L	H	Sc	H
Huang	2007	L	L	L	L	Sc	Sc
Zeng	2009	L	Sc	L	Sc	Sc	Sc
Cao	2010	Sc	Sc	L	Sc	Sc	Sc
He	2010	Sc	Sc	L	H	Sc	H
Hua	2010	Sc	Sc	L	H	Sc	H
Luo	2010	Sc	Sc	L	Sc	Sc	Sc
Yu	2010	Sc	Sc	L	H	Sc	H
Liu	2011	Sc	Sc	L	H	Sc	H
Lu	2011	Sc	Sc	L	Sc	Sc	Sc
Tian	2011	L	Sc	L	Sc	Sc	Sc
Xu	2011	Sc	Sc	L	Sc	Sc	Sc
Yao	2011	L	Sc	L	Sc	Sc	Sc
Zheng	2011	Sc	Sc	L	Sc	Sc	Sc
Jiang	2012	L	L	L	L	Sc	Sc
Liu	2012	Sc	Sc	L	Sc	Sc	Sc
Xie	2012	Sc	Sc	L	H	Sc	H
Zhang	2012	Sc	Sc	L	Sc	Sc	Sc
Zhou	2012a	Sc	Sc	L	Sc	Sc	Sc
Zhou	2012b	Sc	Sc	L	H	Sc	H
Chen	2013	Sc	Sc	L	Sc	Sc	Sc
Ding	2013	Sc	Sc	L	Sc	Sc	Sc
Mo	2013	Sc	Sc	L	Sc	Sc	Sc
Song	2013	Sc	Sc	L	Sc	Sc	Sc
Zhang	2013	Sc	Sc	L	Sc	Sc	Sc
Cheng	2014	Sc	Sc	L	Sc	Sc	Sc
Du	2014	Sc	Sc	L	Sc	Sc	Sc
Li	2014	Sc	Sc	L	Sc	Sc	Sc
Liang	2014	Sc	Sc	L	Sc	Sc	Sc
Liu	2014	Sc	Sc	L	Sc	Sc	Sc
Qiu	2014	Sc	Sc	L	Sc	Sc	Sc
Zhang	2014a	Sc	Sc	L	Sc	Sc	Sc
Zhang	2014b	Sc	Sc	L	Sc	Sc	Sc
Zhang	2014c	Sc	Sc	L	Sc	Sc	Sc
Cai	2015	Sc	Sc	L	Sc	Sc	Sc
Jin	2015a	Sc	Sc	L	Sc	Sc	Sc
Jin	2015b	Sc	Sc	L	Sc	Sc	Sc
Lu	2015a	Sc	Sc	L	H	Sc	H
Lu	2015b	Sc	Sc	L	Sc	Sc	Sc
Ma	2015	Sc	Sc	L	Sc	Sc	Sc
Peng	2015	Sc	Sc	L	Sc	Sc	Sc
Sun	2015	Sc	Sc	L	Sc	Sc	Sc
Wang	2015a	Sc	Sc	L	Sc	Sc	Sc
Wang	2015b	L	Sc	L	H	Sc	H
Wang	2015c	Sc	Sc	L	Sc	Sc	Sc
Yuan	2015	Sc	Sc	L	H	Sc	H
Zhang	2015a	L	Sc	L	Sc	Sc	Sc
Zhang	2015b	L	Sc	L	Sc	Sc	Sc
Zhang	2015c	L	Sc	L	Sc	Sc	Sc
Chen	2016	Sc	Sc	L	Sc	Sc	Sc
He	2016a	Sc	Sc	L	H	Sc	H
He	2016b	Sc	Sc	L	Sc	Sc	Sc
Jiang	2016	Sc	Sc	L	Sc	Sc	Sc
Shan	2016	L	Sc	L	H	Sc	H
Wang	2016a	Sc	Sc	L	H	Sc	H
Wang	2016b	Sc	Sc	L	H	Sc	H
Wu	2016	Sc	Sc	L	Sc	Sc	Sc
Xie	2016	L	Sc	L	Sc	Sc	Sc
Xu	2016	Sc	Sc	L	H	Sc	H
Yang	2016a	Sc	Sc	Sc	H	Sc	H
Yang	2016b	L	Sc	L	Sc	Sc	Sc
Yu	2016	Sc	Sc	L	H	Sc	H
Cao	2017	L	Sc	L	L	Sc	Sc
Cheng	2017	Sc	Sc	L	Sc	Sc	Sc
Ding	2017	L	Sc	L	Sc	Sc	Sc
Du	2017	Sc	Sc	L	Sc	Sc	Sc
Feng	2017	L	Sc	L	Sc	Sc	Sc
Han	2017a	Sc	Sc	L	Sc	Sc	Sc
Han	2017b	Sc	Sc	L	Sc	Sc	Sc
Li	2017	Sc	Sc	L	H	Sc	H
Liu	2017	Sc	Sc	L	Sc	Sc	Sc
Luo	2017	Sc	Sc	L	H	Sc	H
Pang	2017	Sc	Sc	L	Sc	Sc	Sc
Shi	2017	Sc	Sc	L	Sc	Sc	Sc
Song	2017	Sc	Sc	L	Sc	Sc	Sc
Wang	2017	Sc	Sc	L	Sc	Sc	Sc
Wu	2017	Sc	Sc	L	H	Sc	H
Yang	2017	L	Sc	L	Sc	Sc	Sc
Zeng	2017	Sc	Sc	L	Sc	Sc	Sc
Zhang	2017a	Sc	Sc	L	Sc	Sc	Sc
Zhang	2017b	Sc	Sc	L	Sc	Sc	Sc
Zhang	2017c	Sc	Sc	L	Sc	Sc	Sc
Zhang	2017d	Sc	Sc	L	Sc	Sc	Sc
Zhang	2017e	Sc	Sc	L	Sc	Sc	Sc
Zhao	2017	Sc	Sc	L	H	Sc	H
Chai	2018	Sc	Sc	L	Sc	Sc	Sc
Li	2018	L	Sc	L	Sc	Sc	Sc
Liu	2018	Sc	Sc	L	Sc	Sc	Sc
Luo	2018	Sc	Sc	L	Sc	Sc	Sc
Ma	2018a	L	Sc	L	Sc	Sc	Sc
Ma	2018b	Sc	Sc	L	Sc	Sc	Sc
Xiao	2018	L	Sc	L	Sc	Sc	Sc
Xie	2018	Sc	Sc	L	Sc	Sc	Sc
Zhang	2018a	Sc	Sc	L	Sc	Sc	Sc
Zhang	2018b	L	Sc	L	Sc	Sc	Sc
Zhou	2018	Sc	Sc	L	Sc	Sc	Sc
Chen	2019	Sc	Sc	L	Sc	Sc	Sc
Ge	2019	Sc	Sc	L	Sc	Sc	Sc
Han	2019	Sc	Sc	L	H	Sc	H
Hu	2019	L	Sc	L	Sc	Sc	Sc
Lu	2019	L	Sc	L	L	Sc	Sc
Xun	2019	Sc	Sc	L	Sc	Sc	Sc
Yang	2019	Sc	Sc	L	Sc	Sc	Sc
Yao	2019	L	Sc	L	Sc	Sc	Sc
Zhong	2019	Sc	Sc	L	Sc	Sc	Sc
Chen	2020	Sc	Sc	L	H	Sc	H
Hao	2020	L	Sc	L	Sc	Sc	Sc
Ji	2020	Sc	Sc	L	Sc	Sc	Sc
Liu	2020a	Sc	Sc	L	Sc	Sc	Sc
Liu	2020b	Sc	Sc	L	Sc	Sc	Sc
Lu	2020	Sc	Sc	L	Sc	Sc	Sc
Qu	2020	L	Sc	L	Sc	Sc	Sc
Ren	2020	Sc	Sc	L	Sc	Sc	Sc
Shen	2020	Sc	Sc	L	Sc	Sc	Sc
Wu	2020	Sc	Sc	L	Sc	Sc	Sc
Yang	2020	Sc	Sc	L	Sc	Sc	Sc
Zheng	2020	Sc	Sc	L	Sc	Sc	Sc
Jin	2021	Sc	Sc	L	Sc	Sc	Sc
Lan	2021	Sc	Sc	L	Sc	Sc	Sc
Le	2021	Sc	Sc	L	Sc	Sc	Sc
Tang	2021	L	Sc	L	Sc	Sc	Sc
Wang	2021	Sc	Sc	L	Sc	Sc	Sc

D1–D5: 5 domain criteria; D1, bias arising from the randomization process; D2, bias due to deviations from intended interventions; D3, bias due to missing outcome data; D4, bias in the measurement of the outcome; D5, bias in the selection of the reported results; H: high risk of bias; L: low risk of bias; Sc: some concerns.

**Table 3:** Subgroup analysis of the trials that compared IM with CM for PASI score.

	k	MD	95% CI	Heterogeneity (I^2^)	P_subgroup_
**Source of investigational medicine**					**0.0240**
**Research institute prescription**	39	−3.7420	−4.2580 to −3.2261	98.4%	
**Other conventional medicine**	30	−2.8494	−3.4276 to −2.2712	95.9%	
**Formulation type**					**0.0116**
**Decoction**	32	−2.8951	−3.3769 to −2.4132	95.2%	
**Other types**	37	−3.8594	−4.4331 to −2.2856	98.6%	

IM: Integrative medicine; CM: conventional medicine; PASI: psoriasis area severity index.

**Table 4:** Summary of findings for studies in this meta-analysis.

Intervention and Comparator Intervention	Outcomes	Number of Participants (Studies)	Anticipated Absolute Effects (95% CI)	Quality of the Evidence (GRADE)
IM compared to CM for inflammatory pain of rheumatoid arthritis	PASI60	8367 (96)	224 more per 1000 (from 139 more to 318 more)	⨁⨁⨁◯ MODERATE ^a^
	PASI score	5801 (69)	MD 3.3544 PASI score lower (3.7608 lower to 2.9481 lower)	⨁⨁⨁◯ MODERATE ^b^
	PASI70	1276 (13)	239 more per 1000 (From 154 more to 337 more)	⨁⨁⨁◯ MODERATE ^a^
	Recurrence rate	692 (12)	252 fewer per 1000 (From 282 fewer to 210 fewer)	⨁⨁⨁◯ MODERATE ^a^
	DLQI	811 (10)	MD 2.6072 DLQI lower (3.71 lower to 1.5043 lower)	⨁⨁⨁◯ MODERATE ^a^
	VAS	273 (3)	MD 0.89 VAS lower (1.4769 lower to 0.3008 lower)	⨁⨁◯◯ LOW ^a,b^
	TNF-α	997 (12)	SMD 1.9948 SD lower (2.5964 lower to 1.3932 lower)	⨁⨁◯◯ LOW ^a,b^
	IL-8	657 (7)	SMD 1.0752 SD lower (1.9647 lower to 0.1856 lower)	⨁⨁◯◯ LOW ^a,b^
	IL-17	842 (10)	SMD 2.0009 SD lower (3.2927 lower to 0.7091 lower)	⨁⨁◯◯ LOW ^a,b^
	IL-22	348 (4)	SMD 3.1874 SD lower (6.0441 lower to 0.3307 lower)	⨁⨁◯◯ LOW ^a,b^
	IL-23	428 (5)	SMD 1.7398 SD lower (2.6139 lower to 0.8657 lower)	⨁⨁◯◯ LOW ^a,b^
	INF-γ	524 (6)	SMD 2.4211 SD lower (3.2187 lower to 1.6235 lower)	⨁⨁◯◯ LOW ^a,b^

CM, conventional medicine; DLQI, dermatology life quality index; IM, integrative medicine; MD, mean difference; RR, risk ratio; RCT, randomized clinical trial; SD, standardized difference; SMD, standardized mean difference; TNF-α, tumor necrosis factor-α; ^a^: substantial concerns of publication bias; ^b^: the confidence intervals are less overlapping.

**Table 5:** Characters of top 26 commonly prescribed herbs utilized with relatively frequencies exceeding 10% inclusion trials and its prestige centrality.

No	Herbal Material (Latin Name)	Frequency of Prescription	Relative Frequency (%)	PageRank Centrality	Eigenvector Centrality
1	*Rehmannia glutinosa* (Gaertn.) DC.	80	62.5%	0.0284	1
2	*Glycyrrhiza uralensis* Fisch.	57	44.53%	0.0278	0.982
3	*Dictamnus dasycarpus* Turcz.	54	42.19%	0.0272	0.968
4	*Paeonia × suffruticosa* Andrews	53	41.41%	0.0271	0.974
5	*Paeonia anomala subsp. veitchii* (Lynch) D.Y.Hong and K.Y.Pan	51	39.84%	0.0278	0.982
6	*Smilax glabra* Roxb.	47	8.59%	0.0277	0.988
7	*Angelica sinensis* (Oliv.) Diels	44	34.38%	0.0271	0.974
8	*Lonicera japonica* Thunb.	39	30.47%	0.0266	0.953
9	*Saposhnikovia divaricata* (Turcz.) Schischk.	39	30.47%	0.0266	0.945
10	*Arnebia euchroma* (Royle) I.M.Johnst.	36	28.13%	0.0265	0.959
11	*Sophora flavescens* Aiton	36	28.13%	0.0254	0.914
12	*Isatis tinctoria subsp. athoa* (Boiss.) Papan.	35	27.34%	0.0253	0.92
13	*Scrophularia ningpoensis* Hemsl.	33	25.78%	0.0247	0.907
14	*Salvia miltiorrhiza* Bunge	30	23.44%	0.0277	0.988
15	*Cryptotympana dubia* (Haupt)	28	21.88%	0.0253	0.923
16	*Carthamus tinctorius* L.	27	21.09%	0.0224	0.823
17	*Styphnolobium japonicum* (L.) Schott	21	16.41%	0.0224	0.824
18	*Spatholobus suberectus* Dunn	20	15.63%	0.0253	0.92
19	*Arctium lappa* L.	18	14.06%	0.0128	0.442
20	*Scleromitrion diffusum* (Willd.) R.J.Wang	17	13.28%	0.022	0.787
21	*Paeonia lactiflora* Pall.	17	13.28%	0.023	0.843
22	*Ligusticum striatum* DC.	14	10.94%	0.0219	0.793
23	*Ophiopogon japonicus (Thunb.)* Ker Gawl.	14	10.94%	0.0235	0.871
24	*Scutellaria baicalensis* Georgi	14	10.94%	0.0248	0.895
25	*Scutellaria Nepeta tenuifolia* Benth.	13	10.16%	0.0242	0.868
26	*Taraxacum mongolicum* Hand.-Mazz.	13	10.16%	0.0218	0.804

**Table 6:** A priori algorithm-based association rules in the IM component herbs for inflammatory skin lesion in psoriasis.

No	Associations Rules	Support	Confidence	Lift
1	{*Scrophularia ningpoensis* Hemsl.} => {*Rehmannia glutinosa* (Gaertn.) DC.}	0.2500000	0.9696970	1.551515
2	{*Isatis tinctoria* subsp. *athoa* (Boiss.) Papan.} => {*Paeonia × suffruticosa* Andrews}	0.2578125	0.9428571	2.277089
3	{*Isatis tinctoria* subsp. *athoa* (Boiss.) Papan.} => {*Rehmannia glutinosa* (Gaertn.) DC.}	0.2578125	0.9428571	1.508571
4	{*Saposhnikovia divaricata* (Turcz.) Schischk.} => {*Rehmannia glutinosa* (Gaertn.) DC.}	0.2812500	0.9230769	1.476923
5	Paeonia *anomala* subsp. *veitchii* (Lynch) D.Y.Hong and K.Y.Pan} => {*Rehmannia glutinosa* (Gaertn.) DC.}	0.3671875	0.9215686	1.474510
6	{*Paeonia × suffruticosa* Andrews} => {*Rehmannia glutinosa* (Gaertn.) DC.}	0.3828125	0.9245283	1.479245
7	{*Isatis tinctoria* subsp. *athoa* (Boiss.) Papan., *Paeonia × suffruticosa* Andrews} => {*Rehmannia glutinosa* (Gaertn.) DC.}	0.2500000	0.9696970	1.551515
8	{*Isatis tinctoria* subsp. *athoa* (Boiss.) Papan., *Rehmannia glutinosa* (Gaertn.) DC.} => {*Paeonia × suffruticosa* Andrews}	0.2500000	0.9696970	2.341910
9	{*Paeonia × suffruticosa* Andrews, *Paeonia anomala* subsp. *veitchii* (Lynch) D.Y.Hong and K.Y.Pan} => {*Rehmannia glutinosa* (Gaertn.) DC.}	0.2734375	0.9210526	1.473684

**Table 7:** Main information of four core herbs identified through this review.

Four Core Herbs	Interquartile Range of Dosage (g/Day)	Interquartile Range of Treatment Duration (Week)
*Rehmannia glutinosa* (Gaertn.) DC.	15–20 g	4–12 w
*Isatis tinctoria* subsp. *athoa* (Boiss.) Papan.	10–30 g	8–12 w
*Paeonia × suffruticosa* Andrews	10–15 g	8–12 w
*Scrophularia ningpoensis* Hemsl.	10–15 g	7–12 w

g: gram; w: weeks.

**Table 8:** Major active compounds of four core herbs.

Molecule Number	Molecule Name	Chemical Structure	ADME Evaluation				
			Lipsinski	Ghose	Veber	Egan	Muegge
1	Dihydrochelerythrine	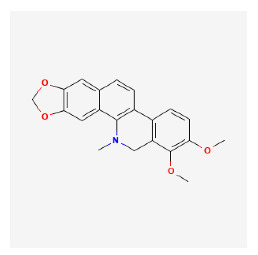	Yes	Yes	Yes	Yes	Yes
2	Sitogluside	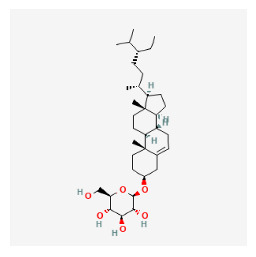	Yes	No	Yes	Yes	No
3	Glycyrol	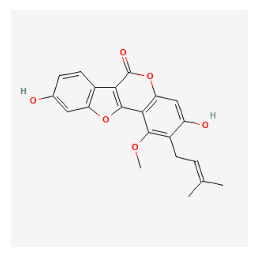	Yes	Yes	Yes	Yes	Yes
4	Hesperetin	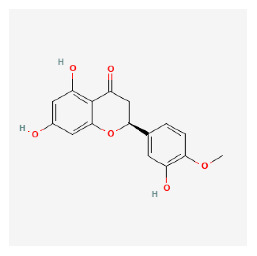	Yes	Yes	Yes	Yes	Yes
5	Salutaridine	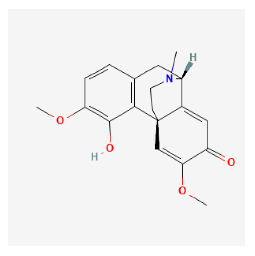	Yes	Yes	Yes	Yes	Yes
6	indirubin	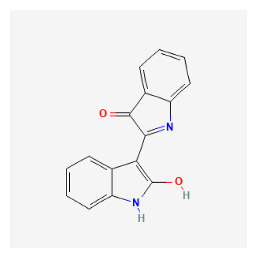	Yes	Yes	Yes	Yes	Yes
7	qingdainone	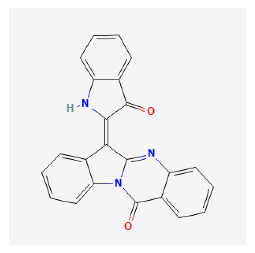	Yes	Yes	Yes	Yes	Yes
8	indigo	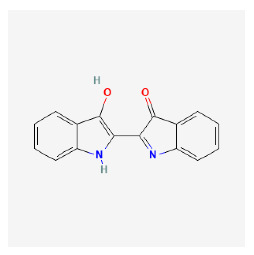	Yes	Yes	Yes	Yes	Yes
9	Quindoline	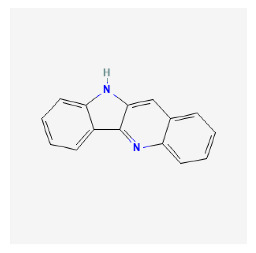	Yes	Yes	Yes	Yes	Yes
10	Indicaxanthin	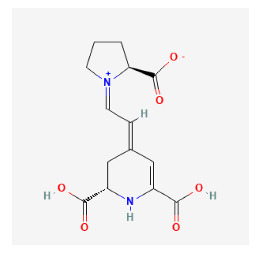	Yes	No	Yes	Yes	Yes
11	Paeoniflorgenin	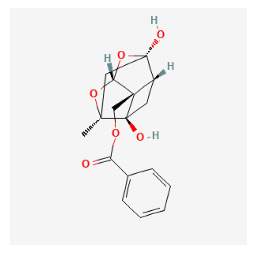	Yes	Yes	Yes	Yes	Yes
12	6-Deglucosyl-3-O-methylpaeoniflorin	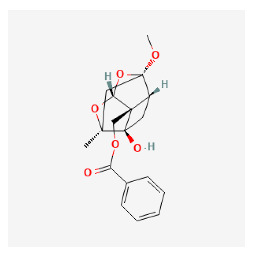	Yes	Yes	Yes	Yes	Yes
13	(2R,3R)-2-(3,5-dihydroxyphenyl)-3,5,7-trihydroxy-2,3-dihydrochromen-4-one	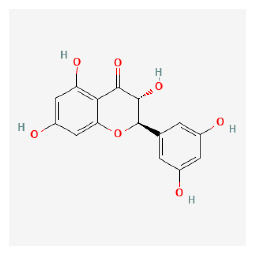	Yes	Yes	Yes	Yes	Yes
14	Columbianetin acetate	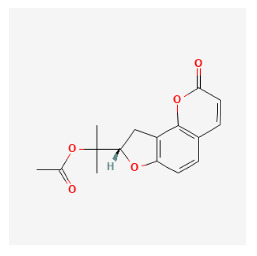	Yes	Yes	Yes	Yes	Yes
15	Cianidanol	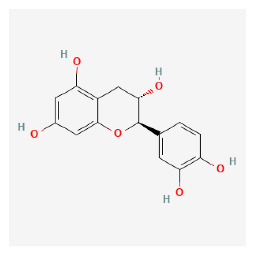	Yes	Yes	Yes	Yes	Yes
16	Triptolide	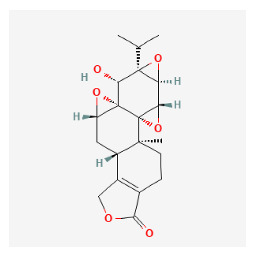	Yes	Yes	Yes	Yes	Yes
17	quercetin	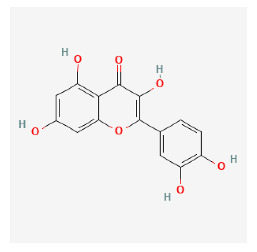	Yes	Yes	Yes	Yes	Yes
18	5-[5-(4-Methoxy-phenyl)-furan-2-ylmethylene]-pyrimidine-2,4,6-trione	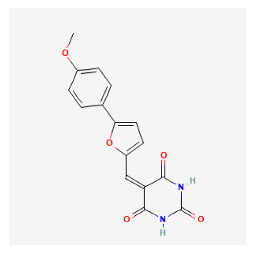	Yes	Yes	Yes	Yes	Yes
19	kaempferol	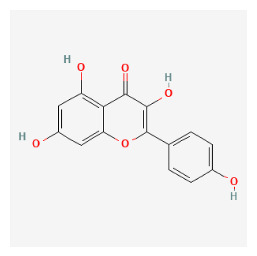	Yes	Yes	Yes	Yes	Yes
20	Sugiol	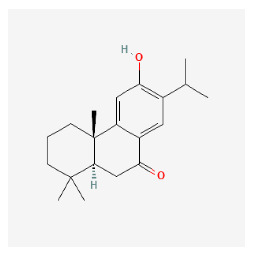	Yes	Yes	Yes	Yes	No
21	Imperatorin	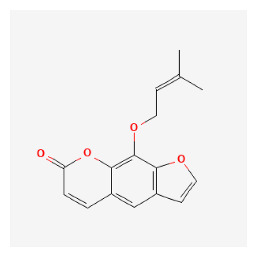	Yes	Yes	Yes	Yes	Yes
22	Palmitoleic acid	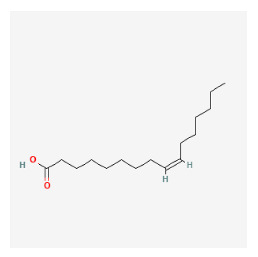	Yes	Yes	No	Yes	No

ADME: absorption, distribution, metabolism, and excretion.

**Table 9:** The 19 hub gene targets.

Node Rank	Gene Target	MCC Centrality	Degree Centrality	Betweenness Centrality	Closeness Centrality
1	STAT3	1.01 × 10^9^	24	75.10296	31.58333
2	CASP3	1.01 × 10^9^	19	12.27081	29.08333
3	PTGS2	1.01 × 10^9^	27	284.44838	33.08333
4	BCL2	1.01 × 10^9^	24	102.82056	31.58333
5	MMP9	1.01 × 10^9^	24	144.3609	31.58333
6	EGFR	1.01 × 10^9^	22	69.20533	30.58333
7	ESR1	1.01 × 10^9^	21	305.04296	30.33333
8	CCND1	1.01 × 10^9^	18	12.97538	28.58333
9	STAT1	1.01 × 10^9^	21	42.05261	30.08333
10	NFKB1	1.01 × 10^9^	22	39.82745	30.58333
11	MAPK8	9.59 × 10^8^	16	8.18678	27.58333
12	CASP9	9.58 × 10^8^	15	5.20558	27.08333
13	JAK2	5.23 × 10^8^	19	123.91169	29.08333
14	MAPK14	4.90 × 10^8^	16	5.94696	27.25
15	KIT	4.04 × 10^7^	14	8.86641	26.58333
16	PDGFRB	1.09 × 10^7^	17	30.99454	28.08333
17	SERPINE1	7,297,946	14	34.3894	26.25
18	CCR2	453,610	15	58.75556	26.61667
19	NOS2	413,280	12	3.37798	24.95

## Data Availability

Data sharing not applicable.

## Supplementary Materials

The following supporting information can be downloaded at: https://www.mdpi.com/article/10.3390/ph16081160/s1, Figure S1: (A) Bubble plot of the meta-regression analysis for type of comparator. (B) Bubble plot of the meta-regression analysis for treatment duration. (C) Bubble plot of the meta-regression analysis for sample size. (D) Bubble plot of the meta-regression analysis for overall risk of bias. (E) Bubble plot of the meta-regression analysis for randomization method; Table S1: Search terms used in each databases; Table S2: Detailed information on East Asian herbal medicine utilized as a component of integrative medicine interventions; Table S3: Prestige centrality of each of the East Asian herbs used in the clinical trials of 5 percent or more; Table S4: Potential gene targets for bioactive compounds from four core herbs; Table S5: Detailed information on potential psoriasis targets from the GeneCard database.
