# Supplementary material for: Efficacy and Key Materials of East Asian Herbal Medicine Combined with Conventional Medicine on Inflammatory Skin Lesion in Patients with Psoriasis Vulgaris: A Meta-Analysis, Integrated Data Mining, and Network Pharmacology
Source: Pharmaceuticals (Basel). 2023 Aug 15;16(8):1160. doi: 10.3390/ph16081160 (PMC10459676; doi:10.3390/ph16081160)
Supplement: Supplementary file 1 [file pharmaceuticals-16-01160-s001.zip › Supplementary figure S1A-E.pdf]

Efficacy and Key Materials of East Asian Herbal Medicine Combined with Conventional Medicine on Inflammatory Skin Lesion in Patients with *Psoriasis Vulgaris*: A Meta-Analysis, Integrated Data Mining, and Network Pharmacology

Hee-Geun Jo [1,2,†], Hyehwa Kim [3,†], Eunhye Baek [4], Donghun Lee [1,*] and Ji Hye Hwang [5,*]

Supplmentary Figures S1A – E

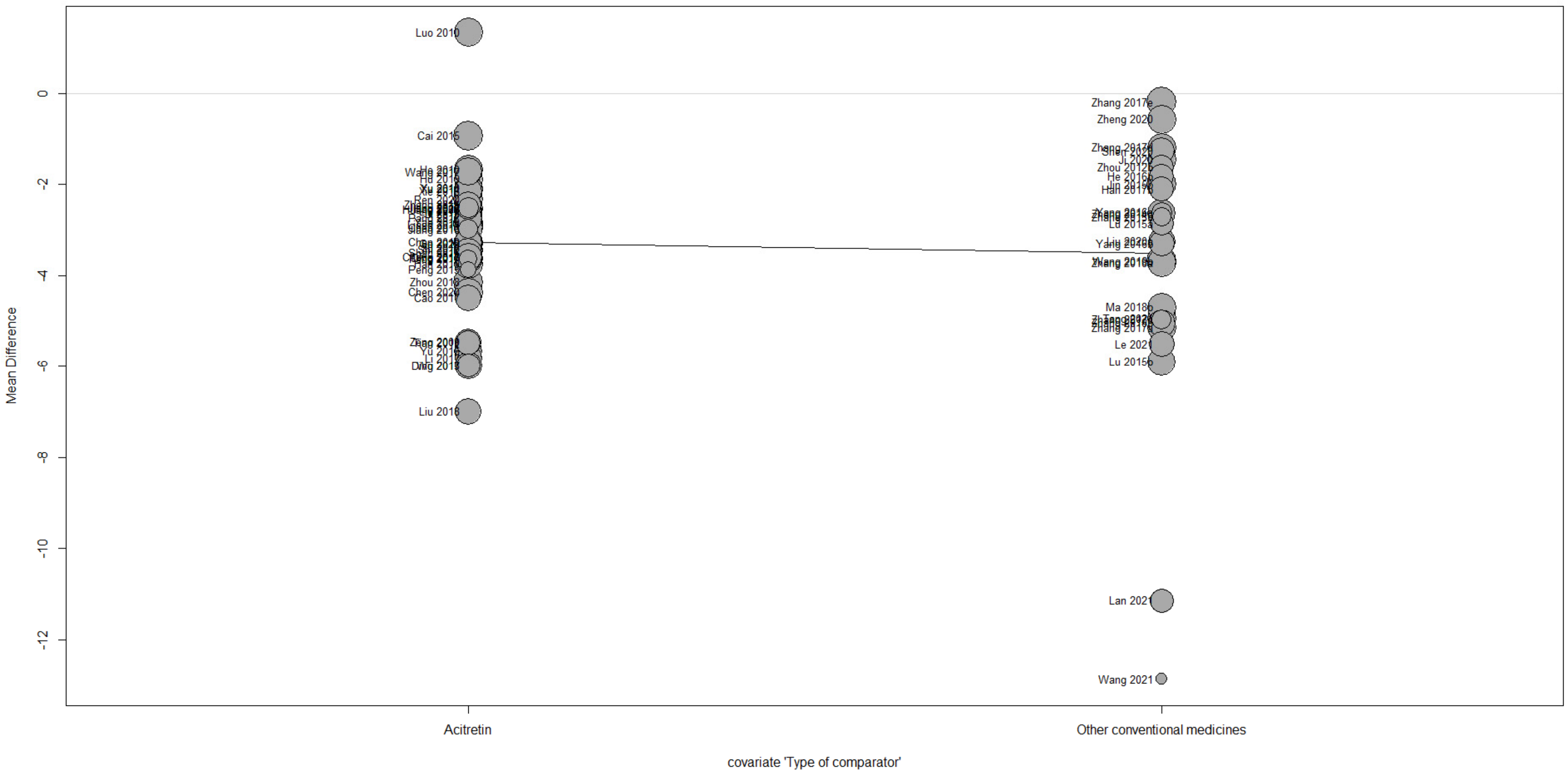

Figure S1 (A) Bubble plot of the meta-regression analysis for type of comparator.

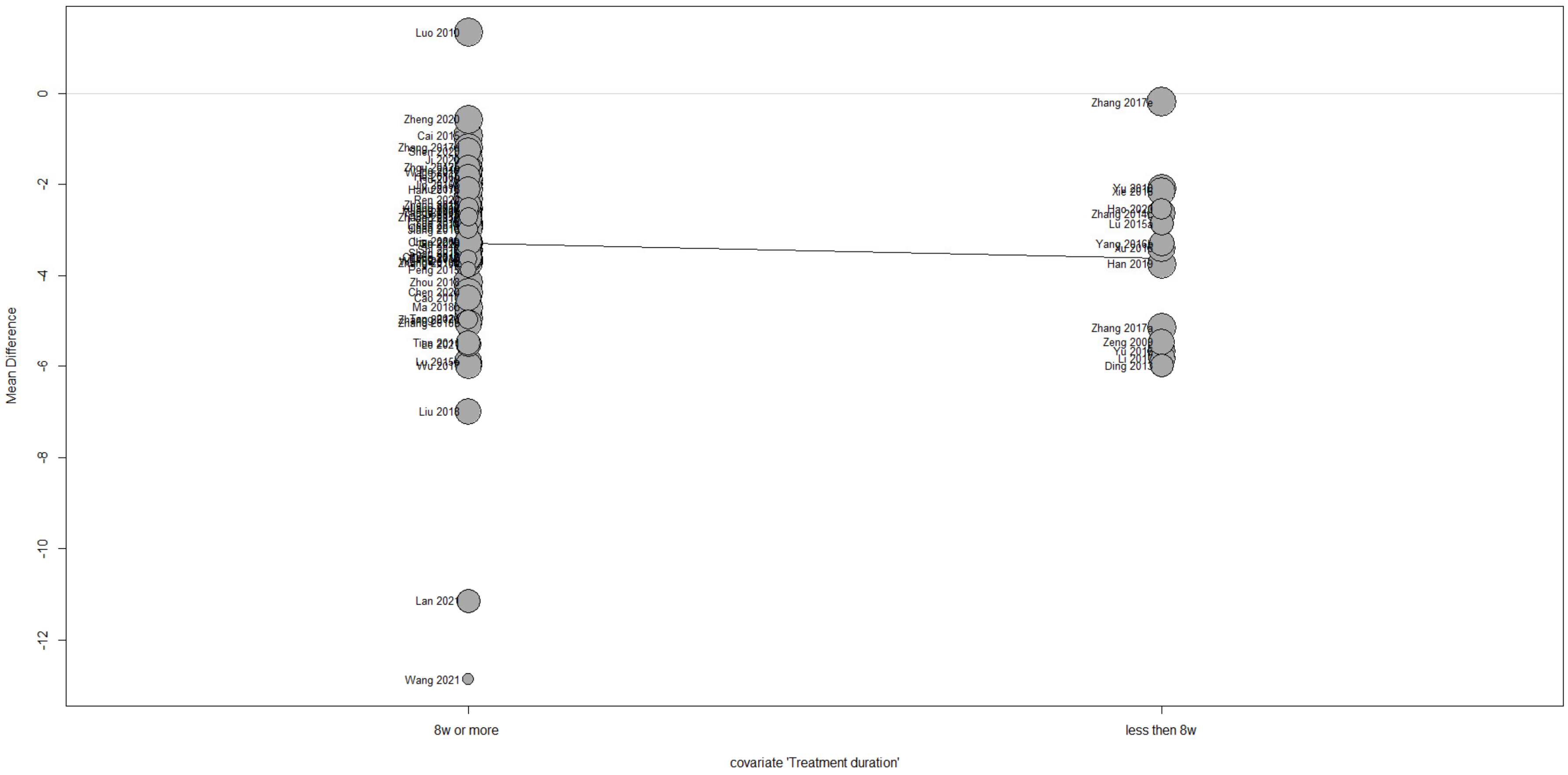

Figure S1 (B) Bubble plot of the meta-regression analysis for treatment duration.

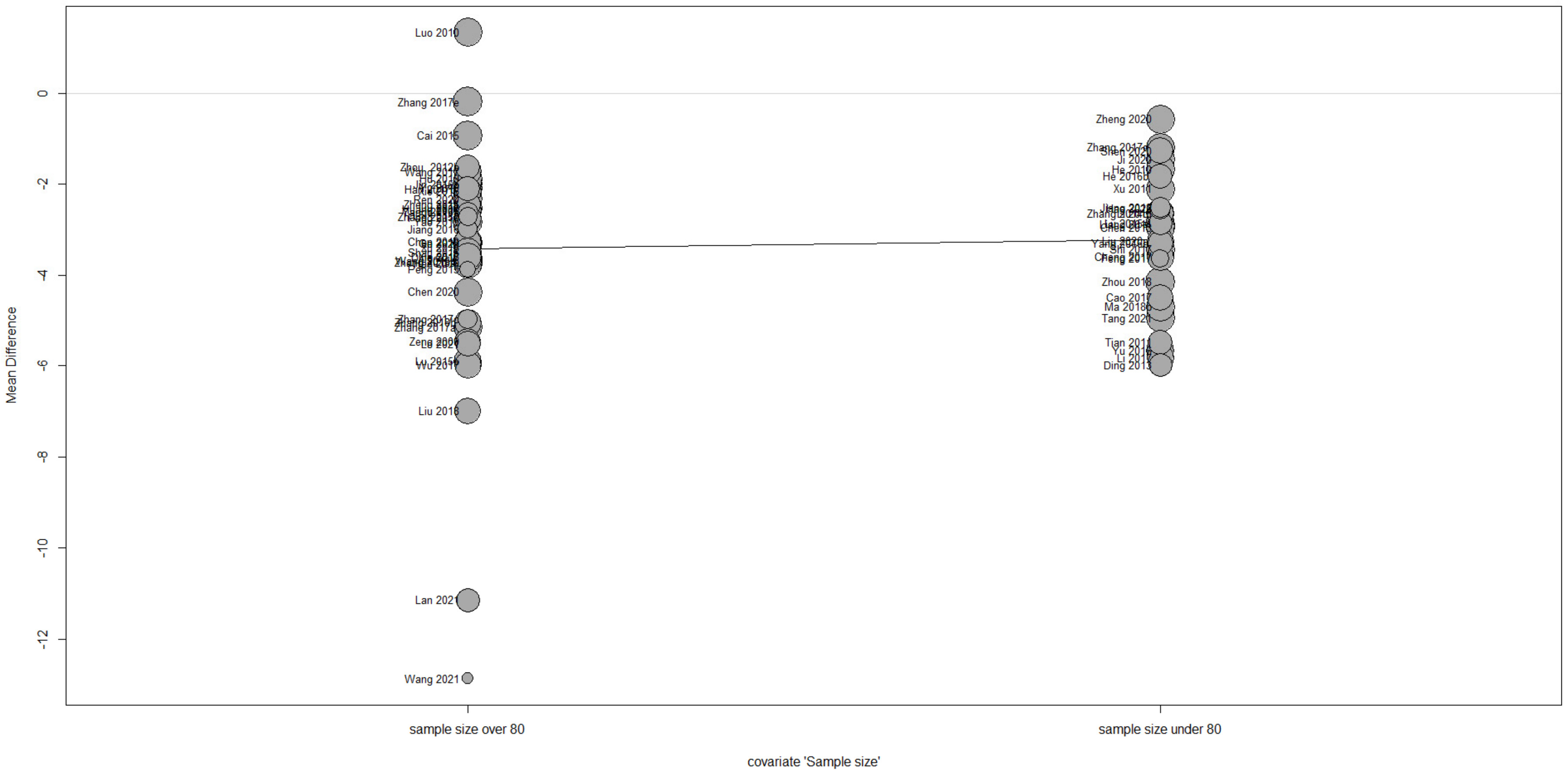

Figure S1 (C) Bubble plot of the meta-regression analysis for sample size.

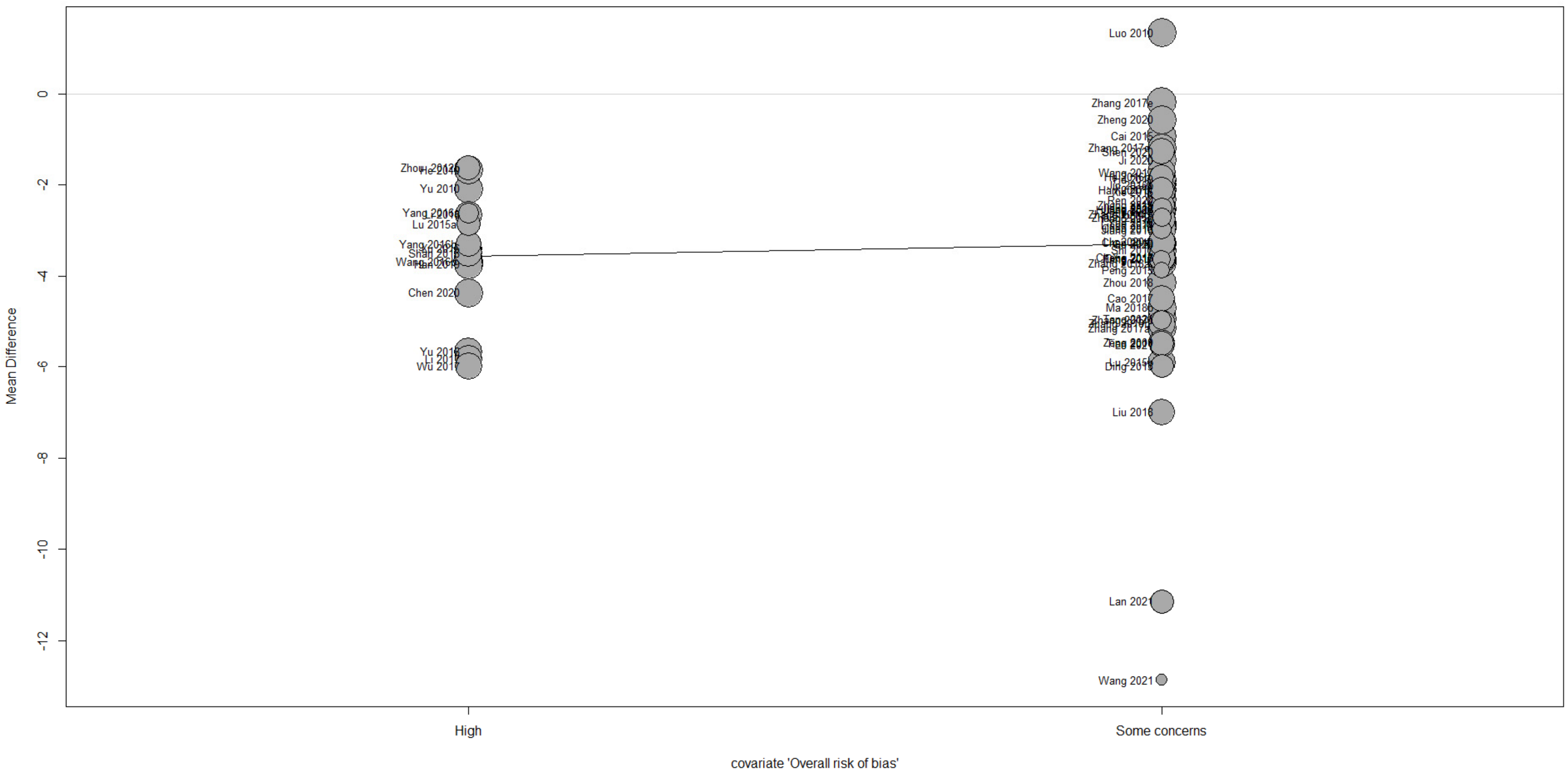

Figure S1 (D) Bubble plot of the meta-regression analysis for overall risk of bias.

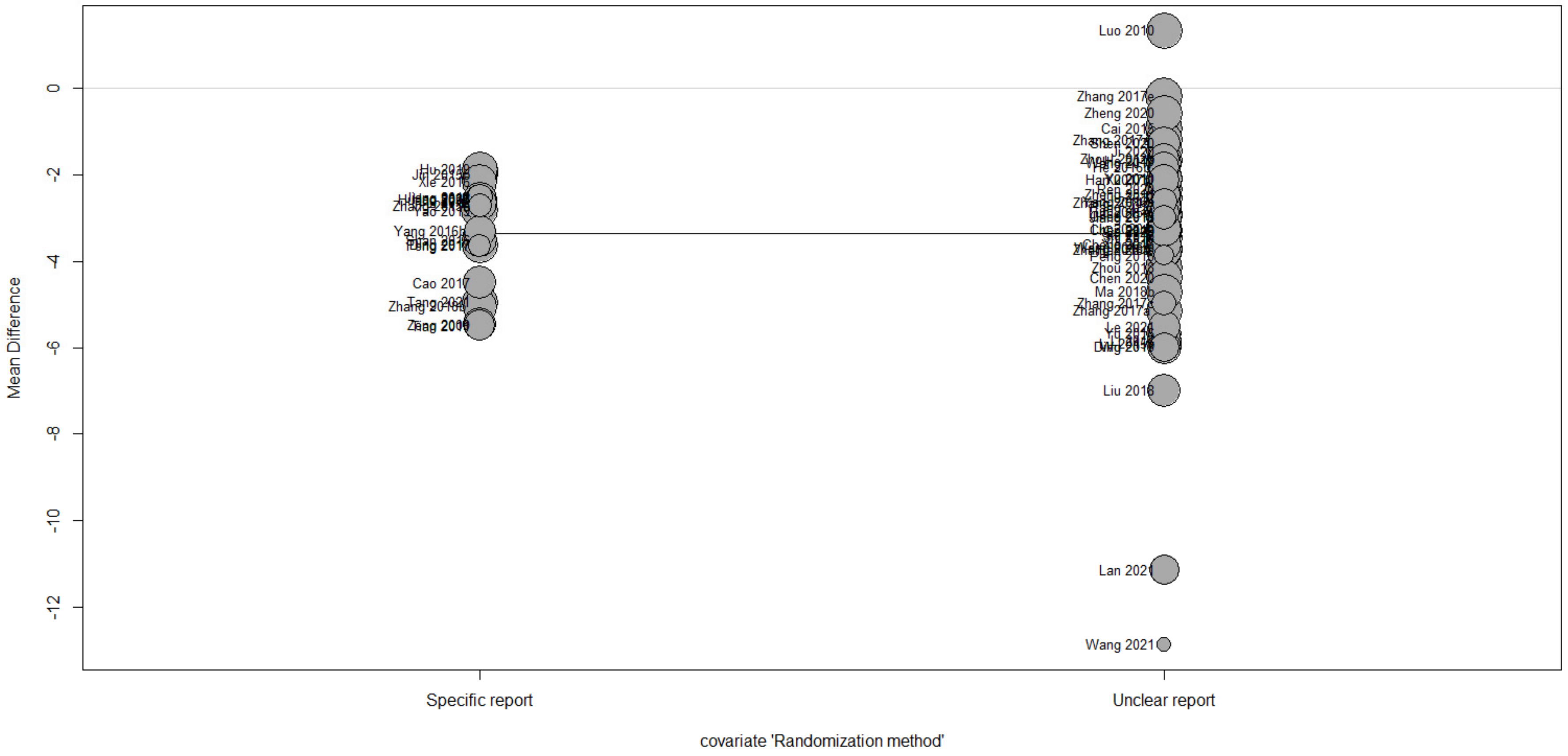

Figure S1 (E) Bubble plot of the meta-regression analysis for randomization method.
