# Supplementary material for: Efficacy and Key Materials of East Asian Herbal Medicine Combined with Conventional Medicine on Inflammatory Skin Lesion in Patients with Psoriasis Vulgaris: A Meta-Analysis, Integrated Data Mining, and Network Pharmacology
Source: Pharmaceuticals (Basel). 2023 Aug 15;16(8):1160. doi: 10.3390/ph16081160 (PMC10459676; doi:10.3390/ph16081160)
Supplement: Supplementary file 1 [file pharmaceuticals-16-01160-s001.zip › Supplementary Table S2.pdf]

Supplementary Table S2. Detailed information on East Asian herbal medicine utilized as a component of integrative medicine interventions.

Study	EAHM prescription name	Source	Ingredients of EAHM prescription (Latin name)	Ingredients of EAHM prescription (Scientific name)	Types of preparation	Quality control reported? (Y/N)	Chemical analysis reported? (Y/N)
Che (2004)	Erdonghuoxue decoction	Prepared by Researchers	*Asparagi tuber, Liriopis seu ophiopogonis tuber, Scrophulariae radix, Angelicae sinensis radix, Spatholobi caulis, Rehmanniae radix recens, Smilax glabrae rhizoma, Salviae miltiorrhizae radix, Dictamni radicis cortex. Indigo pulverata*	Asparagus cochinchinensis (Lour.) Merr. [Asparagaceae], Ophiopogon japonicus (Thunb.) Ker Gawl. [Asparagaceae], Scrophularia ningpoensis Hemsl. [Scrophulariaceae], Angelica sinensis (Oliv.) Diels [Apiaceae], Spatholobus suberectus Dunn	Decoction	N	N

			levis, Persicae semen	[Fabaceae], Rehmannia glutinosa (Gaertn.) DC. [Orobanchaceae](Rehmanniae Radix Recens), Smilax glabra Roxb. [Smilacaceae], Salvia miltiorrhiza Bunge [Lamiaceae], Dictamnus dasycarpus Turcz. [Rutaceae], Persicaria tinctoria (Aiton) Spach [Polygonaceae], Prunus persica (L.) Batsch [Rosaceae]			
Chen (2004)	Anti-psoriasis formula	Prepared by Researchers	*Lithospermi radix, Polygoni cuspidati rhizoma et radix, Lonicerae flos, Salviae miltiorrhizaeradix*	Arnebia euchroma (Royle ex Benth.) I.M.Johnst. [Boraginaceae], Reynoutria japonica Houtt. [Polygonaceae],	Decoction	N	N

, Rehmanniae radix recens, Platycodonis radix, Isatidis folium, Moutan radicis cortex, Paeoniae radix rubra, Sophorae flos, Angelicae sinensis radix, Spatholobi caulis	Lonicera japonica Thunb. [Caprifoliaceae], Salvia miltiorrhiza Bunge [Lamiaceae], Rehmannia glutinosa (Gaertn.) DC. [Orobanchaceae](Rehmanniae Radix Recens), Platycodon grandiflorus (Jacq.) A.DC. [Campanulaceae], Isatis tinctoria subsp. tinctoria [Brassicaceae], Paeonia × suffruticosa Andrews [Paeoniaceae], Paeonia anomala subsp. veitchii (Lynch) D.Y.Hong & K.Y.Pan [Paeoniaceae], Styphnolobium

				japonicum (L.) Schott [Fabaceae], Angelica sinensis (Oliv.) Diels [Apiaceae], Spatholobus suberectus Dunn [Fabaceae]			
Xu (2005)	Liangxuejiedu decoction	Prepared by Researchers	*Rehmanniae radix recens, Lithospermi radix, Sophorae flos, Scrophulariae radix, Smilax glabrae rhizoma, Dictamni radicis cortex, Sophorae radix, Hedyotidis herba, Scutellariae barbatae herba*	Rehmannia glutinosa (Gaertn.) DC. [Orobanchaceae](Rehmanniae Radix Recens), Arnebia euchroma (Royle ex Benth.) I.M.Johnst. [Boraginaceae], Styphnolobium japonicum (L.) Schott [Fabaceae], Scrophularia ningpoensis Hemsl. [Scrophulariaceae], Smilax glabra Roxb. [Smilacaceae], Dictamnus dasycarpus Turcz.	Decoction	N	N

				[Rutaceae], Sophora flavescens Aiton [Fabaceae], Scleromitrion diffusum (Willd.) R.J.Wang [Rubiaceae], Scutellaria barbata D.Don [Lamiaceae]			
Liu (2006)	Jianpiyishen decoction	Prepared by Researchers	*Poria s clerotium. Codonopsis pilosulae radix, Atractylodis rhizoma alba, Dolichoris semen, Dioscoreae rhizoma, Epimedii herba, Astragali radix, Smilax glabrae rhizoma, Hedyotidis herba*	Poria cocos Wolf [Polyporaceae], Codonopsis pilosula (Franch.) Nannf. [Campanulaceae], Atractylodes lancea (Thunb.) DC. [Asteraceae], Lablab purpureus subsp. purpureus [Fabaceae], Dioscorea japonica Thunb. [Dioscoreaceae], Epimedium brevicornu Maxim. [Berberidaceae],	Decoction	N	N

				Astragalus mongholicus Bunge [Fabaceae], Smilax glabra Roxb. [Smilacaceae], Scleromitrion diffusum (Willd.) R.J.Wang [Rubiaceae]			
Chen (2007)	Xiaoyin granule	Commercial supplier Shaanxi Kanghui Pharmaceutical Co., Ltd.	*Rehmanniae radix recens, Moutan radicis cortex, Paeoniae radix rubra, Angelicae sinensis radix, Sophorae radix, Lonicerae flos, Scrophulariae radix, Arctii fructus, Cicadidae periostracum, Dictamni radicis cortex, Isatidis folium, Carthami flos, Saposhnikoviae*	Rehmannia glutinosa (Gaertn.) DC. [Orobanchaceae](Rehmanniae Radix Recens), Paeonia × suffruticosa Andrews [Paeoniaceae], Paeonia anomala subsp. veitchii (Lynch) D.Y.Hong & K.Y.Pan [Paeoniaceae], Angelica sinensis (Oliv.) Diels [Apiaceae], Sophora flavescens Aiton [Fabaceae], Lonicera	Granule	N	N

			radix	japonica Thunb. [Caprifoliaceae], Scrophularia ningpoensis Hemsl. [Scrophulariaceae], Arctium lappa L. [Asteraceae], Cryptotympana dubia (Haupt) [Cicadidae], Dictamnus dasycarpus Turcz. [Rutaceae], Isatis tinctoria subsp. tinctoria [Brassicaceae], Carthamus tinctorius L. [Asteraceae], Saposhnikovia divaricata (Turcz. ex Ledeb.) Schischk. [Apiaceae]			
Huang (2007)	Yinxieling tablet	Prepared by Researcher	*Angelicae sinensis radix, Rehmanniae radix*	Angelica sinensis (Oliv.) Diels [Apiaceae],	Table	N	N

s	*recens, Cnidii rhizoma, Paeoniae radix rubra, Curcumae rhizoma, Lithospermi radix, Smilax glabrae rhizoma, Mume fructus, Glycyrrhizae radix et rhizoma*	Rehmannia glutinosa (Gaertn.) DC. [Orobanchaceae](Rehmanniae Radix Recens), Conioselinum anthriscoides 'Chuanxiong' [Apiaceae], Paeonia anomala subsp. veitchii (Lynch) D.Y.Hong & K.Y.Pan [Paeoniaceae], Curcuma phaeocaulis Valeton [Zingiberaceae], Arnebia euchroma (Royle ex Benth.) I.M.Johnst. [Boraginaceae], Smilax glabra Roxb. [Smilacaceae], Prunus mume (Siebold) Siebold & Zucc. [Rosaceae],

				Glycyrrhiza uralensis Fisch. ex DC. [Fabaceae]			
Zeng (2009)	Xiaoyin granule	Commercial supplier Shaanxi Kanghui Pharmaceutical Co., Ltd.	*Rehmanniae radix recens, Moutan radicis cortex, Paeoniae radix rubra, Angelicae sinensis radix, Sophorae radix, Lonicerae flos, Scrophulariae radix, Arctii fructus, Cicadidae periostracum, Dictamni radicis cortex, Isatidis folium, Carthami flos, Saposhnikoviae radix*	Rehmannia glutinosa (Gaertn.) DC. [Orobanchaceae](Rehmanniae Radix Recens), Paeonia × suffruticosa Andrews [Paeoniaceae], Paeonia anomala subsp. veitchii (Lynch) D.Y.Hong & K.Y.Pan [Paeoniaceae], Angelica sinensis (Oliv.) Diels [Apiaceae], Sophora flavescens Aiton [Fabaceae], Lonicera japonica Thunb. [Caprifoliaceae], Scrophularia ningpoensis Hemsl. [Scrophulariaceae],	Granule	N	N

				Arctium lappa L. [Asteraceae], Cryptotympana dubia (Haupt) [Cicadidae], Dictamnus dasycarpus Turcz. [Rutaceae], Isatis tinctoria subsp. tinctoria [Brassicaceae], Carthamus tinctorius L. [Asteraceae], Saposhnikovia divaricata (Turcz. ex Ledeb.) Schischk. [Apiaceae]			
Cao (2010)	Yangzhen decoction	Prepared by Researchers	*Rehmanniae radix recens, Carthami flos, Paeoniae radix rubra, Sparganii rhizoma, Curcumae rhizoma,*	Rehmannia glutinosa (Gaertn.) DC. [Orobanchaceae](Rehmanniae Radix Recens), Carthamus tinctorius L. [Asteraceae], Paeonia anomala subsp.	Decoction	N	N

Lonicerae flos, Smilax glabrae rhizoma, Schizonepetae spica, Saposhnikoviae radix, Tribuli fructus	veitchii (Lynch) D.Y.Hong & K.Y.Pan [Paeoniaceae], Sparganium stoloniferum (Buch.-Ham. ex Graebn.) Buch.-Ham. ex Juz. [Typhaceae], Curcuma phaeocaulis Valeton [Zingiberaceae], Lonicera japonica Thunb. [Caprifoliaceae], Smilax glabra Roxb. [Smilacaceae], Nepeta tenuifolia Benth. [Lamiaceae], Saposhnikovia divaricata (Turcz. ex Ledeb.) Schischk. [Apiaceae], Tribulus terrestris L. [Zygophyllaceae]

He (2010)	Xiaoranquadan feng	Prepared by Researchers	*Isatidis folium, Dryopteridis crassirhizomatis rhizoma, Taraxaci herba, Paeoniae radix rubra, Moutan radicis cortex, Lithospermi radix, Rehmanniae radix recens, Angelicae sinensis radix, Cnidii rhizoma, Schizonepetae spica, Saposhnikoviae radix, Dictamni radicis cortex, Dioscorea hypoglauca, Smilax glabrae rhizoma, Glycyrrhizae radix et rhizoma*	Isatis tinctoria subsp. tinctoria [Brassicaceae], Dryopteris crassirhizoma Nakai [Polypodiaceae], Taraxacum mongolicum Hand.-Mazz. [Asteraceae], Paeonia anomala subsp. veitchii (Lynch) D.Y.Hong & K.Y.Pan [Paeoniaceae], Paeonia × suffruticosa Andrews [Paeoniaceae], Arnebia euchroma (Royle ex Benth.) I.M.Johnst. [Boraginaceae], Rehmannia glutinosa (Gaertn.) DC. [Orobanchaceae](Rehmanniae Radix	Decoction	N	N

Recens), Angelica sinensis (Oliv.) Diels [Apiaceae], Conioselinum anthriscoides 'Chuanxiong' [Apiaceae], Nepeta tenuifolia Benth. [Lamiaceae], Saposhnikovia divaricata (Turcz. ex Ledeb.) Schischk. [Apiaceae], Dictamnus dasycarpus Turcz. [Rutaceae], Dioscorea collettii var. hypoglauca (Palib.) S.J.Pei & C.T.Ting [Dioscoreaceae], Smilax glabra Roxb. [Smilacaceae], Glycyrrhiza uralensis Fisch. ex DC. [Fabaceae]

Hua (2010)	Qingyinjiedu decoction	Prepared by Researchers	*Smilax glabrae rhizoma, Indigo pulverata levis, Lonicerae flos, Salviae miltiorrhizae radix, Gardenia jasminoides, Smilacis chinae rhizoma, Euphorbia humifusa, Gypsum fibrosum, Scutellariae radix, Rehmanniae radix recens, Sophorae Radix, Dictamni radicis cortex, Curcumae radix, Hedyotidis herba*	Smilax glabra Roxb. [Smilacaceae], Persicaria tinctoria (Aiton) Spach [Polygonaceae], Lonicera japonica Thunb. [Caprifoliaceae], Salvia miltiorrhiza Bunge [Lamiaceae], Gardenia jasminoides J.Ellis [Rubiaceae], Smilax china L. [Smilacaceae], Euphorbia hirta L. [Euphorbiaceae], Gypsum Fibrosum, Scutellaria baicalensis Georgi [Lamiaceae], Rehmannia glutinosa (Gaertn.) DC. [Orobanchaceae](Rehmanniae Radix Recens), Sophora flavescens Aiton [Fabaceae], Dictamnus	Decoction	N	N

				dasycarpus Turcz. [Rutaceae], Curcuma phaeocaulis Valeton [Zingiberaceae], Scleromitrion diffusum (Willd.) R.J.Wang [Rubiaceae]			
Luo (2010)	Piminxiao capsule	Pharmaceutical product (specific commercial supplier information not reported)	*Sophorae radix, Atractylodis rhizoma, Saposhnikoviae radix, Schizonepetae spica, Tribuli fructus, Dictamni radicis cortex, Cnidi fructus, Xanthii fructus, Scolopendra, Indigo pulverata levis, Taraxaci herba, Violae herba, Scutellariae radix, Phellodendri cortex, Coptis chinensis,*	Sophora flavescens Aiton [Fabaceae], Atractylodes lancea (Thunb.) DC. [Asteraceae](Atractylodis Rhizoma), Saposhnikovia divaricata (Turcz. ex Ledeb.) Schischk. [Apiaceae], Nepeta tenuifolia Benth. [Lamiaceae], Tribulus terrestris L. [Zygophyllaceae], Dictamnus dasycarpus Turcz. [Rutaceae], Cnidium monnieri (L.) Cusson [Apiaceae], Xanthium strumarium L.	Capsule	N	N

Cicadidae periostracum, Rehmanniae radix recens, Moutan radicis cortex, Tamarics cacumen, Lithospermi radix, Lycii radicis cortex	[Asteraceae], Scolopendra subspinipes mutilans Linné Koch [Scolopendridae], Persicaria tinctoria (Aiton) Spach [Polygonaceae], Taraxacum mongolicum Hand.-Mazz. [Asteraceae], Viola mandshurica W.Becker [Violaceae], Scutellaria baicalensis Georgi [Lamiaceae], Phellodendron chinense C.K.Schneid. [Rutaceae], Coptis chinensis Franch. [Ranunculaceae], Cryptotympana dubia (Haupt) [Cicadidae], Rehmannia glutinosa (Gaertn.) DC. [Orobanchaceae](Reh

				manniae Radix Recens), Paeonia × suffruticosa Andrews [Paeoniaceae], Tamarix chinensis Lour. [Tamaricaceae], Arnebia euchroma (Royle ex Benth.) I.M.Johnst. [Boraginaceae], Lycium barbarum L. [Solanaceae]			
Yu (2010)	Runzaozhiyang capsule	Commercial supplier Guizhou Tongji Tang Pharmaceutical Co., Ltd.	*Polygoni Multiflori radix, Rehmanniae radix recens, Mori folium, Sophorae radix, Girardiana heterophylla decne*	Reynoutria multiflora (Thunb.) Moldenke [Polygonaceae], Rehmannia glutinosa (Gaertn.) DC. [Orobanchaceae](Rehmanniae Radix Recens), Morus alba L. [Moraceae], Sophora flavescens Aiton [Fabaceae], Girardinia diversifolia subsp. diversifolia [Urticaceae]	Capsule	N	N

Liu (2011)	Fangfengtongsheng powder	Prepared by Researchers	*Cnidii rhizoma, Angelicae sinensis radix, Paeonia lactiflora, Rhei radix et rhizoma, Gypsum fibrosum, Scutellariae radix, Gardenia jasminoides, Forsythiae fructus, Natrii sulfas, Saposhnikoviae radix, Menthae herba, Schizonepetae spica, Ephedrae herba*	Conioselinum anthriscoides 'Chuanxiong' [Apiaceae], Angelica sinensis (Oliv.) Diels [Apiaceae], Paeonia lactiflora Pall. [Paeoniaceae](Paeonia lactiflora Alba), Rheum officinale Baill. [Polygonaceae], Gypsum Fibrosum, Scutellaria baicalensis Georgi [Lamiaceae], Gardenia jasminoides J.Ellis [Rubiaceae], Forsythia suspensa (Thunb.) Vahl [Oleaceae], Natrii sulfas, Saposhnikovia divaricata (Turcz. ex Ledeb.) Schischk. [Apiaceae], Mentha canadensis L. [Lamiaceae], Mentha canadensis L. [Lamiaceae], Nepeta	Granule	N	N

				tenuifolia Benth. [Lamiaceae], Ephedra sinica Stapf [Ephedraceae]			
Lu (2011)	Qingrejiedu decoction	Prepared by Researchers	*Moutan radicis cortex, Paeoniae radix rubra, Isatidis radix, Gardenia jasminoides, Smilax glabrae rhizoma, Sophorae flos, Dictamni radicis cortex, Angelicae sinensis radix, Spatholobi caulis, Tripterygium wilfordii, Scolopendra*	Paeonia × suffruticosa Andrews [Paeoniaceae], Paeonia anomala subsp. veitchii (Lynch) D.Y.Hong & K.Y.Pan [Paeoniaceae], Isatis tinctoria subsp. tinctoria [Brassicaceae](Isatidis Radix), Gardenia jasminoides J.Ellis [Rubiaceae], Smilax glabra Roxb. [Smilacaceae], Styphnolobium japonicum (L.) Schott [Fabaceae], Dictamnus dasycarpus Turcz. [Rutaceae], Angelica	Decoction	N	N

				sinensis (Oliv.) Diels [Apiaceae], Spatholobus suberectus Dunn [Fabaceae], Tripterygium wilfordii Hook.f. [Celastraceae], Scolopendra subspinipes mutilans Linné Koch [Scolopendridae]			
Tian (2011)	Qingfeiliangxue decoction	Prepared by Researchers	*Bubalus bubalis, Rehmanniae radix recens, Paeoniae radix rubra, Moutan radicis cortex, Angelicae sinensis radix, Dictamni radicis cortex, Spatholobi caulis, Smilax glabrae rhizoma, Lithospermi radix, Lonicerae flos, Scutellariae barbatae herba,*	Bubalus bubalis Linné [Bovidae], Rehmannia glutinosa (Gaertn.) DC. [Orobanchaceae](Rehmanniae Radix Recens), Paeonia anomala subsp. veitchii (Lynch) D.Y.Hong & K.Y.Pan [Paeoniaceae], Paeonia × suffruticosa Andrews [Paeoniaceae], Angelica sinensis	Decoction	N	N

	Hedyotidis herba, Zaocys, Salviae Miltiorrhizae radix, Glycyrrhizae radix et rhizoma	(Oliv.) Diels [Apiaceae], Dictamnus dasycarpus Turcz. [Rutaceae], Spatholobus suberectus Dunn [Fabaceae], Smilax glabra Roxb. [Smilacaceae], Arnebia euchroma (Royle ex Benth.) I.M.Johnst. [Boraginaceae], Lonicera japonica Thunb. [Caprifoliaceae], Scutellaria barbata D.Don [Lamiaceae], Scleromitrion diffusum (Willd.) R.J.Wang [Rubiaceae], Zaocys dhumnades Cantor [Colubridae], Salvia miltiorrhiza Bunge [Lamiaceae],

				Glycyrrhiza uralensis Fisch. ex DC. [Fabaceae]			
Xu (2011)	Qingxuanyin	Prepared by Researchers	*Rehmanniae radix recens, Lonicerae flos, Forsythiae fructus, Anemarrhenae rhizoma, Dictamni radicis cortex, Sophorae radix, Cicadidae periostracum, Tribuli fructus, Moutan radicis cortex, Paeoniae radix rubra, Gypsum fibrosum, Glycyrrhizae radix et rhizoma*	Rehmannia glutinosa (Gaertn.) DC. [Orobanchaceae](Rehmanniae Radix Recens), Lonicera japonica Thunb. [Caprifoliaceae], Forsythia suspensa (Thunb.) Vahl [Oleaceae], Anemarrhena asphodeloides Bunge [Asparagaceae], Dictamnus dasycarpus Turcz. [Rutaceae], Sophora flavescens Aiton [Fabaceae], Cryptotympana dubia (Haupt) [Cicadidae], Tribulus terrestris L.	Decoction	N	N

				[Zygophyllaceae], Paeonia × suffruticosa Andrews [Paeoniaceae], Paeonia anomala subsp. veitchii (Lynch) D.Y.Hong & K.Y.Pan [Paeoniaceae], Gypsum Fibrosum, Glycyrrhiza uralensis Fisch. ex DC. [Fabaceae]			
Yao (2011)	Xiaoyin granule	Commercial supplier Shaanxi Kanghui Pharmaceutical Co., Ltd.	*Rehmanniae radix recens, Moutan radicis cortex, Paeoniae radix rubra, Angelicae sinensis radix, Sophorae radix, Lonicerae flos, Scrophulariae radix, Arctii fructus, Cicadidae periostracum, Dictamni radicis cortex, Isatidis*	Rehmannia glutinosa (Gaertn.) DC. [Orobanchaceae](Rehmanniae Radix Recens), Paeonia × suffruticosa Andrews [Paeoniaceae], Paeonia anomala subsp. veitchii (Lynch) D.Y.Hong & K.Y.Pan [Paeoniaceae], Angelica sinensis (Oliv.) Diels	Granule	N	N

			folium, Carthami flos, Saposhnikoviae radix	[Apiaceae], Sophora flavescens Aiton [Fabaceae], Lonicera japonica Thunb. [Caprifoliaceae], Scrophularia ningpoensis Hemsl. [Scrophulariaceae], Arctium lappa L. [Asteraceae], Cryptotympana dubia (Haupt) [Cicadidae], Dictamnus dasycarpus Turcz. [Rutaceae], Isatis tinctoria subsp. tinctoria [Brassicaceae], Carthamus tinctorius L. [Asteraceae], Saposhnikovia divaricata (Turcz. ex Ledeb.) Schischk. [Apiaceae]			
Zheng (2011)	Xiaoyinkeji decoction	Prepared by	*Bubalus bubalis, Rehmanniae radix*	Bubalus bubalis Linné [Bovidae],	Decoction	N	N

Researchers	*recens , Moutan radicis cortex, Paeoniae radix rubra , Smilax glabrae rhizoma, Sophorae radix, Dictamni radicis cortex, Kochiae fructus, Lithospermi radix, Cicadidae periostracum, Scutellariae radix, Saposhnikoviae radix, Imperatae rhizoma, Atractylodis rhizoma*	Rehmannia glutinosa (Gaertn.) DC. [Orobanchaceae](Rehmanniae Radix Recens), Paeonia × suffruticosa Andrews [Paeoniaceae], Paeonia anomala subsp. veitchii (Lynch) D.Y.Hong & K.Y.Pan [Paeoniaceae] , Smilax glabra Roxb. [Smilacaceae], Sophora flavescens Aiton [Fabaceae], Dictamnus dasycarpus Turcz. [Rutaceae], Bassia scoparia (L.) A.J.Scott [Amaranthaceae], Arnebia euchroma (Royle ex Benth.) I.M.Johnst. [Boraginaceae], Cryptotympana dubia (Haupt)

				[Cicadidae], Scutellaria baicalensis Georgi [Lamiaceae], Saposhnikovia divaricata (Turcz. ex Ledeb.) Schischk. [Apiaceae], Imperata cylindrica (L.) Raeusch. [Poaceae], Atractylodes lancea (Thunb.) DC. [Asteraceae](Atractylodis Rhizoma)			
Jiang (2012)	Sendi particles	Prepared by Researchers	*Rehmanniae radix recens, Scrophulariae radix, Moutan radicis cortex, Paeoniae radix rubra, Chrysanthmi flos, Taraxaci herba, Isatidis radix, Violae herba, Gardenia jasminoides, Smilax glabrae*	Rehmannia glutinosa (Gaertn.) DC. [Orobanchaceae](Rehmanniae Radix Recens), Scrophularia ningpoensis Hemsl. [Scrophulariaceae], Paeonia × suffruticosa Andrews [Paeoniaceae], Paeonia anomala subsp. veitchii (Lynch) D.Y.Hong & K.Y.Pan	Granule	N	N

rhizoma, Fritillariae thunbergii bulbus, Trichosanthes kirilowii, Platycodonis radix, Glycyrrhizae radix et rhizoma	[Paeoniaceae], Chrysanthemum × morifolium (Ramat.) Hemsl. [Asteraceae], Taraxacum mongolicum Hand.-Mazz. [Asteraceae], Isatis tinctoria subsp. tinctoria [Brassicaceae](Isatidis Radix), Viola mandshurica W.Becker [Violaceae], Gardenia jasminoides J.Ellis [Rubiaceae], Smilax glabra Roxb. [Smilacaceae], Fritillaria thunbergii Miq. [Liliaceae], Trichosanthes kirilowii Maxim. [Cucurbitaceae], Platycodon grandiflorus (Jacq.) A.DC. [Campanulaceae], Glycyrrhiza uralensis

				Fisch. ex DC. [Fabaceae]			
Liu (2012)	Runzaozhiyang capsule	Commercial supplier Guizhou Tongji Tang Pharmaceutical Co., Ltd.	*Polygoni multiflori radix, Rehmanniae radix recens, Mori folium, Sophorae radix, Girardiana heterophylla decne*	Reynoutria multiflora (Thunb.) Moldenke [Polygonaceae], Rehmannia glutinosa (Gaertn.) DC. [Orobanchaceae](Rehmanniae Radix Recens), Morus alba L. [Moraceae], Sophora flavescens Aiton [Fabaceae], Girardinia diversifolia subsp. diversifolia [Urticaceae]	Capsule	N	N
Xie (2012)	Runzaozhiyang capsule	Commercial supplier Guizhou Tongji Tang Pharmaceutical Co.,	*Polygoni multiflori radix, Rehmanniae radix recens, Mori folium, Sophorae radix, Girardiana heterophylla*	Reynoutria multiflora (Thunb.) Moldenke [Polygonaceae], Rehmannia glutinosa (Gaertn.) DC. [Orobanchaceae](Rehmanniae Radix	Capsule	N	N

		Ltd.	*decne*	Recens), Morus alba L. [Moraceae], Sophora flavescens Aiton [Fabaceae], Girardinia diversifolia subsp. diversifolia [Urticaceae]			
Zhang (2012)	Yinxie capsule	Commercial supplier Xinjiang Weiatang Pharmaceutical Co., Ltd.	*Smilax glabrae rhizoma, Smilacis chinae rhizoma*	Smilax glabra Roxb. [Smilacaceae], Smilax china L. [Smilacaceae]	Capsule	N	N
Zhou (2012a)	Xiaoyin granule	Commercial supplier Shaanxi Kanghui Pharmaceutical Co., Ltd.	*Rehmanniae radix recens, Moutan radicis cortex, Paeoniae radix rubra, Angelicae sinensis radix, Sophorae radix, Lonicerae flos, Scrophulariae radix, Arctii fructus, Cicadidae periostracum,*	Rehmannia glutinosa (Gaertn.) DC. [Orobanchaceae](Rehmanniae Radix Recens), Paeonia × suffruticosa Andrews [Paeoniaceae], Paeonia anomala subsp. veitchii (Lynch) D.Y.Hong & K.Y.Pan [Paeoniaceae],	Granule	N	N

Dictamni radicis cortex, Isatidis folium, Carthami flos, Saposhnikoviae radix	Angelica sinensis (Oliv.) Diels [Apiaceae], Sophora flavescens Aiton [Fabaceae], Lonicera japonica Thunb. [Caprifoliaceae], Scrophularia ningpoensis Hemsl. [Scrophulariaceae], Arctium lappa L. [Asteraceae], Cryptotympana dubia (Haupt) [Cicadidae], Dictamnus dasycarpus Turcz. [Rutaceae], Isatis tinctoria subsp. tinctoria [Brassicaceae], Carthamus tinctorius L. [Asteraceae], Saposhnikovia divaricata (Turcz. ex Ledeb.) Schischk. [Apiaceae]

Zhou (2012b)	Xiaoyin granule	Commercial supplier Shaanxi Kanghui Pharmaceutical Co., Ltd.	*Rehmanniae radix recens, Moutan radicis cortex, Paeoniae radix rubra, Angelicae sinensis radix, Sophorae radix, Lonicerae flos, Scrophulariae radix, Arctii fructus, Cicadidae periostracum, Dictamni radicis cortex, Isatidis folium, Carthami flos, Saposhnikoviae radix*	Rehmannia glutinosa (Gaertn.) DC. [Orobanchaceae](Rehmanniae Radix Recens), Paeonia × suffruticosa Andrews [Paeoniaceae], Paeonia anomala subsp. veitchii (Lynch) D.Y.Hong & K.Y.Pan [Paeoniaceae], Angelica sinensis (Oliv.) Diels [Apiaceae], Sophora flavescens Aiton [Fabaceae], Lonicera japonica Thunb. [Caprifoliaceae], Scrophularia ningpoensis Hemsl. [Scrophulariaceae], Arctium lappa L. [Asteraceae], Cryptotympana dubia (Haupt) [Cicadidae],	Granule	N	N

				Dictamnus dasycarpus Turcz. [Rutaceae], Isatis tinctoria subsp. tinctoria [Brassicaceae], Carthamus tinctorius L. [Asteraceae], Saposhnikovia divaricata (Turcz. ex Ledeb.) Schischk. [Apiaceae]			
Chen (2013)	Xiaoyin decoction	Prepared by Researchers	*Paeoniae radix rubra, Rehmanniae radix recens, Smilax glabrae rhizoma, Lonicerae flos, Dictamni radicis cortex, Angelicae sinensis radix, Cnidii rhizoma, Lithospermi radix, Salviae miltiorrhizae radix*	Paeonia anomala subsp. veitchii (Lynch) D.Y.Hong & K.Y.Pan [Paeoniaceae], Rehmannia glutinosa (Gaertn.) DC. [Orobanchaceae](Rehmanniae Radix Recens), Smilax glabra Roxb. [Smilacaceae], Lonicera japonica Thunb. [Caprifoliaceae],	Decoction	N	N

				Dictamnus dasycarpus Turcz. [Rutaceae], Angelica sinensis (Oliv.) Diels [Apiaceae], Conioselinum anthriscoides 'Chuanxiong' [Apiaceae], Arnebia euchroma (Royle ex Benth.) I.M.Johnst. [Boraginaceae], Salvia miltiorrhiza Bunge [Lamiaceae]			
Ding (2013)	NiupixuanⅡ decoction	Prepared by Researchers	*Lonicerae flos, Isatidis radix, Lithospermi radix, Imperatae rhizoma, Rehmanniae radix recens, Rubiae radix, Moutan radicis cortex, Trichosanthes kirilowii, Smilax glabrae rhizoma, Persicae semen,*	Lonicera japonica Thunb. [Caprifoliaceae], Isatis tinctoria subsp. tinctoria [Brassicaceae](Isatidis Radix), Arnebia euchroma (Royle ex Benth.) I.M.Johnst. [Boraginaceae], Imperata cylindrica (L.) Raeusch. [Poaceae],	Decoction	N	N

	Carthami flos, Mume fructus, Dictamni radicis cortex, Glycyrrhizae radix et rhizoma	Rehmannia glutinosa (Gaertn.) DC. [Orobanchaceae](Rehmanniae Radix Recens), Rubia tinctorum L. [Rubiaceae], Paeonia × suffruticosa Andrews [Paeoniaceae], Trichosanthes kirilowii Maxim. [Cucurbitaceae], Smilax glabra Roxb. [Smilacaceae], Prunus persica (L.) Batsch [Rosaceae], Carthamus tinctorius L. [Asteraceae], Prunus mume (Siebold) Siebold & Zucc. [Rosaceae], Dictamnus dasycarpus Turcz. [Rutaceae], Glycyrrhiza uralensis Fisch. ex DC.	

				[Fabaceae]			
Mo (2013)	Total Glycosides of Paeoniae Alba capsule	Commercial supplier Ningbo Lihua Pharmaceutical Co., Ltd.	*Paeonia lactiflora*	Paeonia lactiflora Pall. [Paeoniaceae](Paeonia lactiflora Alba)	Capsule	N	N
Song (2013)	Xiaoyin granule	Commercial supplier Shaanxi Kanghui Pharmaceutical Co., Ltd.	*Rehmanniae radix recens, Moutan radicis cortex, Paeoniae radix rubra, Angelicae sinensis radix, Sophorae radix, Lonicerae flos, Scrophulariae radix, Arctii fructus, Cicadidae periostracum, Dictamni radicis cortex, Isatidis folium, Carthami*	Rehmannia glutinosa (Gaertn.) DC. [Orobanchaceae](Rehmanniae Radix Recens), Paeonia × suffruticosa Andrews [Paeoniaceae], Paeonia anomala subsp. veitchii (Lynch) D.Y.Hong & K.Y.Pan [Paeoniaceae], Angelica sinensis (Oliv.) Diels [Apiaceae], Sophora	Granule	N	N

	flos, Saposhnikoviae radix	flavescens Aiton [Fabaceae], Lonicera japonica Thunb. [Caprifoliaceae], Scrophularia ningpoensis Hemsl. [Scrophulariaceae], Arctium lappa L. [Asteraceae], Cryptotympana dubia (Haupt) [Cicadidae], Dictamnus dasycarpus Turcz. [Rutaceae], Isatis tinctoria subsp. tinctoria [Brassicaceae], Carthamus tinctorius L. [Asteraceae], Saposhnikovia divaricata (Turcz. ex Ledeb.) Schischk. [Apiaceae]

Zhang (2013)	Xiaoyin capsule	Commercial supplier Shaanxi Shaanxi Mormid Pharmaceutical Co., Ltd.	*Rehmanniae radix recens, Moutan radicis cortex, Paeoniae radix rubra, Angelicae sinensis radix, Sophorae radix, Lonicerae flos, Scrophulariae radix, Arctii fructus, Cicadidae periostracum, Dictamni radicis cortex, Isatidis folium, Carthami flos, Saposhnikoviae radix*	Rehmannia glutinosa (Gaertn.) DC. [Orobanchaceae](Rehmanniae Radix Recens), Paeonia × suffruticosa Andrews [Paeoniaceae], Paeonia anomala subsp. veitchii (Lynch) D.Y.Hong & K.Y.Pan [Paeoniaceae], Angelica sinensis (Oliv.) Diels [Apiaceae], Sophora flavescens Aiton [Fabaceae], Lonicera japonica Thunb. [Caprifoliaceae], Scrophularia ningpoensis Hemsl. [Scrophulariaceae], Arctium lappa L. [Asteraceae], Cryptotympana dubia (Haupt) [Cicadidae],	Capsule	N	N

				Dictamnus dasycarpus Turcz. [Rutaceae], Isatis tinctoria subsp. tinctoria [Brassicaceae], Carthamus tinctorius L. [Asteraceae], Saposhnikovia divaricata (Turcz. ex Ledeb.) Schischk. [Apiaceae]			
Cheng (2014)	Yanghe decoction	Prepared by Researchers	*Rehmanniae radix preparata, Cervi cornus colla, Zingiberis rhizoma, Cinnamomi cortex, Ephedrae herba, Sinapis alba semen, Glycyrrhizae radix et rhizoma*	Rehmannia glutinosa (Gaertn.) DC. [Orobanchaceae](Rehmanniae Radix Preparata), Cervi Cornus Colla, Zingiberis Rhizoma, Cinnamomi Cortex, Ephedra sinica Stapf [Ephedraceae], Sinapis alba L. [Brassicaceae], Glycyrrhiza uralensis Fisch. ex DC.	Decoction	N	N

				[Fabaceae]			
Du (2014)	Xiaoyin granule	Commercial supplier Shaanxi Kanghui Pharmaceutical Co., Ltd.	*Rehmanniae radix recens, Moutan radicis cortex, Paeoniae radix rubra, Angelicae sinensis radix, Sophorae fadix, Lonicerae flos, Scrophulariae radix, Arctii fructus, Cicadidae periostracum, Dictamni radicis cortex, Isatidis folium, Carthami flos,*	Rehmannia glutinosa (Gaertn.) DC. [Orobanchaceae](Rehmanniae Radix Recens), Paeonia × suffruticosa Andrews [Paeoniaceae], Paeonia anomala subsp. veitchii (Lynch) D.Y.Hong & K.Y.Pan [Paeoniaceae], Angelica sinensis (Oliv.) Diels [Apiaceae], Sophora flavescens Aiton	Granule	N	N

			Saposhnikoviae radix	[Fabaceae], Lonicera japonica Thunb. [Caprifoliaceae], Scrophularia ningpoensis Hemsl. [Scrophulariaceae], Arctium lappa L. [Asteraceae], Cryptotympana dubia (Haupt) [Cicadidae], Dictamnus dasycarpus Turcz. [Rutaceae], Isatis tinctoria subsp. tinctoria [Brassicaceae], Carthamus tinctorius L. [Asteraceae], Saposhnikovia divaricata (Turcz. ex Ledeb.) Schischk. [Apiaceae]			
Li (2014)	Huanglianjie du decoction	Prepared by Researcher s	*Coptis chinensis, Scutellariae radix, Phellodendri cortex, Gardenia*	Coptis chinensis Franch. [Ranunculaceae], Scutellaria baicalensis	Decoction	N	N

			jasminoides, Smilax glabrae rhizoma, Hedyotidis herba, Ligustri fructus, Ecliptae herba	Georgi [Lamiaceae], Phellodendron chinense C.K.Schneid. [Rutaceae], Gardenia jasminoides J.Ellis [Rubiaceae], Smilax glabra Roxb. [Smilacaceae], Scleromitrion diffusum (Willd.) R.J.Wang [Rubiaceae], Ligustrum lucidum W.T.Aiton [Oleaceae], Eclipta prostrata (L.) L. [Asteraceae]			
Liang (2014)	Qinzhuliangxue feng	Prepared by Researchers	*Scutellariae radix, Ecliptae herba, Margaritifera concha, Paeoniae radix rubra , Rehmanniae radix recens , Lithospermi radix, Moutan radicis cortex,*	Scutellaria baicalensis Georgi [Lamiaceae], Eclipta prostrata (L.) L. [Asteraceae], Pinctada imbricata Röding [Pteriidae], Paeonia anomala subsp. veitchii (Lynch) D.Y.Hong & K.Y.Pan	Decoction	N	N

Lonicerae flos, Cynanchi paniculati radix et rhizoma, Glycyrrhizae radix et rhizoma, Saposhnikoviae radix, Scrophulariae radix	[Paeoniaceae] , Rehmannia glutinosa (Gaertn.) DC. [Orobanchaceae](Rehmanniae Radix Recens) , Arnebia euchroma (Royle ex Benth.) I.M.Johnst. [Boraginaceae], Paeonia × suffruticosa Andrews [Paeoniaceae], Lonicera japonica Thunb. [Caprifoliaceae], Vincetoxicum mukdenense Kitag. [Apocynaceae], Glycyrrhiza uralensis Fisch. ex DC. [Fabaceae], Saposhnikovia divaricata (Turcz. ex Ledeb.) Schischk. [Apiaceae], Scrophularia ningpoensis Hemsl.

				[Scrophulariaceae]			
Liu (2014)	Runzaozhiyang capsule	Commercial supplier Guizhou Tongji Tang Pharmaceutical Co., Ltd.	*Polygoni multiflori radix, Rehmanniae radix recens, Mori folium, Sophorae radix, Girardiana heterophylla decne*	Reynoutria multiflora (Thunb.) Moldenke [Polygonaceae], Rehmannia glutinosa (Gaertn.) DC. [Orobanchaceae](Rehmanniae Radix Recens), Morus alba L. [Moraceae], Sophora flavescens Aiton [Fabaceae], Girardinia diversifolia subsp. diversifolia [Urticaceae]	Capsule	N	N
Qiu (2014)	Yangxierunfuyin	Prepared by Researchers	*Rehmanniae radix recens, Rehmanniae radix preparata, Scrophulariae radix, Salviae miltiorrhizae radix, Polygoni Multiflori radix, Liriopis seu ophiopogonis*	Rehmannia glutinosa (Gaertn.) DC. [Orobanchaceae](Rehmanniae Radix Recens), Rehmannia glutinosa (Gaertn.) DC. [Orobanchaceae](Rehmanniae Radix Preparata), Scrophularia	Decoction	N	N

	tuber, Asparagi tuber, Cannabis semen, Mori folium, Angelicae sinensis radix, Glycyrrhizae radix et rhizoma, Cicadidae periostracum	ningpoensis Hemsl. [Scrophulariaceae], Salvia miltiorrhiza Bunge [Lamiaceae], Reynoutria multiflora (Thunb.) Moldenke [Polygonaceae], Ophiopogon japonicus (Thunb.) Ker Gawl. [Asparagaceae], Asparagus cochinchinensis (Lour.) Merr. [Asparagaceae], Cannabis sativa L. [Cannabaceae], Morus alba L. [Moraceae], Angelica sinensis (Oliv.) Diels [Apiaceae], Glycyrrhiza uralensis Fisch. ex DC. [Fabaceae], Cryptotympana dubia (Haupt) [Cicadidae]	

Qiu (2014)	Duhuojisheng decoction	Prepared by Researchers	*Loranthi ramulus et folium, Angelica pubescens, Angelicae sinensis radix, Gentianae macrophyllae radix, Cinnamomi ramulus, Saposhnikoviae radix, Cnidii rhizoma, Paeoniae radix rubra, Smilax glabrae rhizoma, Spatholobi caulis, Clematidis radix, Achyranthis radix, Glycyrrhizae radix et rhizoma*	Loranthus parasticus Merr. [Loranthaceae], Angelica biserrata (R.H.Shan & C.Q.Yuan) C.Q.Yuan & R.H.Shan [Apiaceae], Angelica sinensis (Oliv.) Diels [Apiaceae], Gentiana macrophylla Pall. [Gentianaceae], Neolitsea cassia (L.) Kosterm. [Lauraceae], Saposhnikovia divaricata (Turcz. ex Ledeb.) Schischk. [Apiaceae], Conioselinum anthriscoides ‘Chuanxiong’ [Apiaceae], Paeonia anomala subsp. veitchii (Lynch) D.Y.Hong & K.Y.Pan [Paeoniaceae], Smilax glabra Roxb. [Smilacaceae],	Decoction	N	N

				Spatholobus suberectus Dunn [Fabaceae], Clematis chinensis Osbeck [Ranunculaceae], Achyranthes bidentata Blume [Amaranthaceae], Glycyrrhiza uralensis Fisch. ex DC. [Fabaceae]			
Qiu (2014)	Qingying decoction plus Runzaoyingyang decoction	Prepared by Researchers	*Gypsum fibrosum, Anemarrhenae rhizoma, Scrophulariae radix, Rehmanniae radix recens , Liriopis seu ophiopogonis tuber, Coptis chinensis, Phyllostachys nigra, Forsythiae fructus , Rulvis glycyrrhizae extractionis*	Gypsum Fibrosum, Anemarrhena asphodeloides Bunge [Asparagaceae], Scrophularia ningpoensis Hemsl. [Scrophulariaceae], Rehmannia glutinosa (Gaertn.) DC. [Orobanchaceae](Rehmanniae Radix Recens) , Ophiopogon japonicus (Thunb.) Ker Gawl. [Asparagaceae],	Decoction	N	N

			sedilis, Glycyrrhizae radix et rhizoma	Coptis chinensis Franch. [Ranunculaceae], Phyllostachys nigra var. henonis (Mitford) Rendle [Poaceae], Forsythia suspensa (Thunb.) Vahl [Oleaceae], Rulvis Glycyrrhizae Extractionis Sedilis, Glycyrrhiza uralensis Fisch. ex DC. [Fabaceae]			
Zhang (2014a)	Yinxie capsule	Commercial supplier Xinjiang Viatang Pharmaceutical Co., Ltd.	*Smilax glabrae rhizoma, Smilacis chinae rhizoma*	Smilax glabra Roxb. [Smilacaceae], Smilax china L. [Smilacaceae]	Capsule	N	N
Zhang (2014b)	Ziyinyangxue qufeng decoction	Prepared by Researchers	*Angelicae sinensis radix, Paeonia lactiflora, Cnidii rhizoma, Rehmanniae radix recens, Tribuli*	Angelica sinensis (Oliv.) Diels [Apiaceae], Paeonia lactiflora Pall. [Paeoniaceae](Paeonia lactiflora Alba),	Decoction	N	N

fructus, Saposhnikoviae radix, Schizonepetae spica, Polygoni multiflori radix, Astragali radix, Glycyrrhizae radix et rhizoma, Scolopendra, tripterygium wilfordii	Conioselinum anthriscoides 'Chuanxiong' [Apiaceae], Rehmannia glutinosa (Gaertn.) DC. [Orobanchaceae](Rehmanniae Radix Recens), Tribulus terrestris L. [Zygophyllaceae], Saposhnikovia divaricata (Turcz. ex Ledeb.) Schischk. [Apiaceae], Nepeta tenuifolia Benth. [Lamiaceae], Reynoutria multiflora (Thunb.) Moldenke [Polygonaceae], Astragalus mongholicus Bunge [Fabaceae], Glycyrrhiza uralensis Fisch. ex DC. [Fabaceae], Scolopendra

				subspinipes mutilans Linné Koch [Scolopendridae], Tripterygium wilfordii Hook.f. [Celastraceae]			
Zhang (2014c)	Qingfeiliangxue decoction	Prepared by Researchers	*Bubalus bubalis, Rehmanniae radix recens, Paeoniae radix rubra, Moutan radicis cortex, Angelicae sinensis radix, Dictamni radicis cortex, Spatholobi caulis, Smilax glabrae rhizoma, Lithospermi radix, Lonicerae flos, Scutellariae barbatae herba, Hedyotidis herba, Zaocys, Salviae miltiorrhizae radix*	Bubalus bubalis Linné [Bovidae], Rehmannia glutinosa (Gaertn.) DC. [Orobanchaceae](Rehmanniae Radix Recens), Paeonia anomala subsp. veitchii (Lynch) D.Y.Hong & K.Y.Pan [Paeoniaceae], Paeonia × suffruticosa Andrews [Paeoniaceae], Angelica sinensis (Oliv.) Diels [Apiaceae], Dictamnus dasycarpus Turcz. [Rutaceae], Spatholobus	Decoction	N	N

				suberectus Dunn [Fabaceae], Smilax glabra Roxb. [Smilacaceae], Arnebia euchroma (Royle ex Benth.) I.M.Johnst. [Boraginaceae], Lonicera japonica Thunb. [Caprifoliaceae], Scutellaria barbata D.Don [Lamiaceae], Scleromitrion diffusum (Willd.) R.J.Wang [Rubiaceae], Zaocys dhumnades Cantor [Colubridae], Salvia miltiorrhiza Bunge [Lamiaceae]			
Cai (2015)	Xiaoyin feng	Prepared by Researchers	*Salviae miltiorrhizae radix, Scrophulariae radix, Sophorae radix, Isatidis*	Salvia miltiorrhiza Bunge [Lamiaceae], Scrophularia ningpoensis Hemsl. [Scrophulariaceae], Sophora flavescens	Decoction	N	N

folium, Paeoniae radix rubra, Lithospermi radix, Moutan radicis cortex, Violae herba, Taraxaci herba, Paris polyphylla, Gardenia jasminoides, Forsythiae fructus, Gentianae scabrae radix et rhizoma, Zaocys, Cicadidae periostracum, Lonicerae folium et caulis, Smilax glabrae rhizoma, Dictamni radicis cortex, Euphorbia humifusa, Lonicerae flos, Persicae semen, Carthami flos, Glycyrrhizae radix et rhizoma,	Aiton [Fabaceae], Isatis tinctoria subsp. tinctoria [Brassicaceae], Paeonia anomala subsp. veitchii (Lynch) D.Y.Hong & K.Y.Pan [Paeoniaceae], Arnebia euchroma (Royle ex Benth.) I.M.Johnst. [Boraginaceae], Paeonia × suffruticosa Andrews [Paeoniaceae], Viola mandshurica W.Becker [Violaceae], Taraxacum mongolicum Hand.-Mazz. [Asteraceae], Paris polyphylla, Gardenia jasminoides J.Ellis [Rubiaceae], Forsythia suspensa (Thunb.) Vahl [Oleaceae], Gentiana

Chrysanthmi flos	scabra Bunge [Gentianaceae], Zaocys dhumnades Cantor [Colubridae], Cryptotympana dubia (Haupt) [Cicadidae], Lonicera japonica Thunb. [Caprifoliaceae], Smilax glabra Roxb. [Smilacaceae], Dictamnus dasycarpus Turcz. [Rutaceae], Euphorbia hirta L. [Euphorbiaceae], Lonicera japonica Thunb. [Caprifoliaceae], Prunus persica (L.) Batsch [Rosaceae], Carthamus tinctorius L. [Asteraceae], Glycyrrhiza uralensis Fisch. ex DC. [Fabaceae], Chrysanthemum ×

				morifolium (Ramat.) Hemsl. [Asteraceae]			
Jin (2015a)	Matrin tablet	Commercial supplier Guangdong Yishu Pharmaceutical Co., LTD.	*Sophorae radix*	Sophora flavescens Aiton [Fabaceae]	Tablet	N	N
Jin (2015b)	Liangxuequfeng powder	Prepared by Researchers	*Sophorae radix, Xanthii fructus, Kochiae fructus, Dictamni radicis cortex, Angelicae sinensis radix, Rehmanniae radix recens, Paeoniae radix rubra, Paeonia lactiflora, Zaocys, Scolopendra, Buthus martensii, Cicadidae periostracum, Saposhnikoviae radix, Gentianae scabrae radix et*	Sophora flavescens Aiton [Fabaceae], Xanthium strumarium L. [Asteraceae], Bassia scoparia (L.) A.J.Scott [Amaranthaceae], Dictamni Radicis, Cortex Angelica sinensis (Oliv.) Diels [Apiaceae], Rehmannia glutinosa (Gaertn.) DC. [Orobanchaceae](Rehmanniae Radix Recens), Paeonia anomala subsp.	Powder	N	N

	rhizoma, Thlaspi arvense, Violae herba, Salviae miltiorrhizae radix, Citri unshius pericarpium, Bupleuri radix, Glycyrrhizae radix et rhizoma	veitchii (Lynch) D.Y.Hong & K.Y.Pan [Paeoniaceae], Paeonia lactiflora Pall. [Paeoniaceae](Paeonia lactiflora Alba), Zaocys dhumnades Cantor [Colubridae], Scolopendra subspinipes mutilans Linné Koch [Scolopendridae], Buthus martensii Karsch [Buthidae], Cryptotympana dubia (Haupt) [Cicadidae], Saposhnikovia divaricata (Turcz. ex Ledeb.) Schischk. [Apiaceae], Gentiana scabra Bunge [Gentianaceae], Thlaspi arvense L. [Brassicaceae], Viola mandshurica	

				W.Becker [Violaceae], Salvia miltiorrhiza Bunge [Lamiaceae], Citrus × aurantium f. deliciosa (Ten.) M.Hiroe [Rutaceae], Bupleurum chinense DC. [Apiaceae], Glycyrrhiza uralensis Fisch. ex DC. [Fabaceae]			
Lu (2015a)	Qingying decoction	Prepared by Researchers	*Bubalus bubalis, Moutan radicis cortex, Forsythiae fructus, Lonicerae flos, Rehmanniae radix recens, Taraxaci herba, Scrophulariae radix, Spatholobi caulis, Liriopis seu ophiopogonis tuber, Lithospermi radix*	Bubalus bubalis Linné [Bovidae], Paeonia × suffruticosa Andrews [Paeoniaceae], Forsythia suspensa (Thunb.) Vahl [Oleaceae], Lonicera japonica Thunb. [Caprifoliaceae], Rehmannia glutinosa (Gaertn.) DC. [Orobanchaceae](Rehmanniae Radix Recens), Taraxacum mongolicum Hand.-	Decoction	N	N

				Mazz. [Asteraceae], Scrophularia ningpoensis Hemsl. [Scrophulariaceae], Spatholobus suberectus Dunn [Fabaceae], Ophiopogon japonicus (Thunb.) Ker Gawl. [Asparagaceae], Arnebia euchroma (Royle ex Benth.) I.M.Johnst. [Boraginaceae]			
Lu (2015b)	Yangxierunfuyin	Prepared by Researchers	*Rehmanniae radix recens, Rehmanniae radix Preparata, Angelicae sinensis radix, Paeoniae radix rubra, Asparagi tuber, Liriopis seu ophiopogonis tuber, Trichosanthes*	Rehmannia glutinosa (Gaertn.) DC. [Orobanchaceae](Rehmanniae Radix Recens), Rehmannia glutinosa (Gaertn.) DC. [Orobanchaceae](Rehmanniae Radix Preparata), Angelica sinensis (Oliv.) Diels [Apiaceae], Paeonia	Decoction	N	N

kirilowii, Moutan radicis cortex, Astragali radix, Buthus martensii , Spatholobi caulis, Zaocys, Batryticatus bombyx	anomala subsp. veitchii (Lynch) D.Y.Hong & K.Y.Pan [Paeoniaceae], Asparagus cochinchinensis (Lour.) Merr. [Asparagaceae], Ophiopogon japonicus (Thunb.) Ker Gawl. [Asparagaceae], Trichosanthes kirilowii Maxim. [Cucurbitaceae], Paeonia × suffruticosa Andrews [Paeoniaceae], Astragalus mongholicus Bunge [Fabaceae], Buthus martensii Karsch [Buthidae] , Spatholobus suberectus Dunn [Fabaceae], Zaocys dhumnades Cantor

				[Colubridae], Bombyx mori Linné [Bombycidae]			
Ma (2015)	Liangxuerunfu decoction	Prepared by Researchers	*Rehmanniae radix recens, Isatidis folium, Smilax glabrae rhizoma, Imperatae rhizoma, Sophorae flos, Moutan radicis cortex, Solani nigri herba, Glycyrrhizae radix et rhizoma*	Rehmannia glutinosa (Gaertn.) DC. [Orobanchaceae](Rehmanniae Radix Recens), Isatis tinctoria subsp. tinctoria [Brassicaceae], Smilax glabra Roxb. [Smilacaceae], Imperata cylindrica (L.) Raeusch. [Poaceae], Styphnolobium japonicum (L.) Schott [Fabaceae], Paeonia × suffruticosa Andrews [Paeoniaceae], Solanum nigrum L. [Solanaceae], Glycyrrhiza uralensis Fisch. ex DC. [Fabaceae]	Decoction	N	N
Peng	Yinxiping pill	Prepared	*Rehmanniae radix*	Rehmannia glutinosa	Pill `	N	N

(2015)	by Researcher s	*recens, Hedyotidis herba, Scutellariae barbatae herba, Isatidis folium, Paeoniae radix rubra , Moutan radicis cortex, Salviae miltiorrhizae radix, Lithospermi adix, Ligustri fructus, Ecliptae herba, Liriopis seu ophiopogonis tuber, Dioscoreae rhizoma, Dictamni radicis cortex, Glycyrrhizae radix et rhizoma*	(Gaertn.) DC. [Orobanchaceae](Rehmanniae Radix Recens), Scleromitrion diffusum (Willd.) R.J.Wang [Rubiaceae], Scutellaria barbata D.Don [Lamiaceae], Isatis tinctoria subsp. tinctoria [Brassicaceae], Paeonia anomala subsp. veitchii (Lynch) D.Y.Hong & K.Y.Pan [Paeoniaceae] , Paeonia × suffruticosa Andrews [Paeoniaceae], Salvia miltiorrhiza Bunge [Lamiaceae], Arnebia euchroma (Royle ex Benth.) I.M.Johnst. [Boraginaceae], Ligustrum lucidum

				W.T.Aiton [Oleaceae], Eclipta prostrata (L.) L. [Asteraceae], Ophiopogon japonicus (Thunb.) Ker Gawl. [Asparagaceae], Dioscorea japonica Thunb. [Dioscoreaceae], Dictamnus dasycarpus Turcz. [Rutaceae], Glycyrrhiza uralensis Fisch. ex DC. [Fabaceae]			
Sun (2015)	Qingrexiaoyin decoction	Prepared by Researchers	*Lonicerae flos, Smilax glabrae rhizoma, Hedyotidis herba, Scutellariae barbatae herba, Sophorae tonkinensis radix et rhizoma, Lithospermi radix, Rubiae*	Lonicera japonica Thunb. [Caprifoliaceae], Smilax glabra Roxb. [Smilacaceae], Scleromitrion diffusum (Willd.) R.J.Wang [Rubiaceae], Scutellaria barbata D.Don [Lamiaceae],	Decoction	N	N

			radix, Ecliptae herba, Sophorae flos, Zaocys, Dictamni radicis cortex, Sophorae radix, Cicadidae periostracum, Glycyrrhizae radix et rhizoma	Sophora tonkinensis Gagnep. [Fabaceae], Arnebia euchroma (Royle ex Benth.) I.M.Johnst. [Boraginaceae], Rubia tinctorum L. [Rubiaceae], Eclipta prostrata (L.) L. [Asteraceae], Styphnolobium japonicum (L.) Schott [Fabaceae], Zaocys dhumnades Cantor [Colubridae], Dictamnus dasycarpus Turcz. [Rutaceae], Sophora flavescens Aiton [Fabaceae], Cryptotympana dubia (Haupt) [Cicadidae], Glycyrrhiza uralensis Fisch. ex DC. [Fabaceae]			
Wang	Compound	Commerci	*Glycyrrhizae*	Glycyrrhiza uralensis	Liquid	N	N

(2015a)	glycyrrhizin	al supplier Henan Xin Shuaike Pharmaceutical Co., LTD.	*radix et rhizoma*	Fisch. ex DC. [Fabaceae]			
Wang (2015b)	Researcher prescription	Prepared by Researchers	*Bubalus bubalis, Lonicerae flos, Paeoniae radix rubra, Dictamni radicis cortex, Moutan radicis cortex, Coptis chinensis, Forsythiae fructus, Sophorae radix, Glycyrrhizae radix et rhizoma*	Bubalus bubalis Linné [Bovidae], Lonicera japonica Thunb. [Caprifoliaceae], Paeonia anomala subsp. veitchii (Lynch) D.Y.Hong & K.Y.Pan [Paeoniaceae], Dictamnus dasycarpus Turcz. [Rutaceae], Paeonia × suffruticosa Andrews [Paeoniaceae], Coptis chinensis Franch. [Ranunculaceae], Forsythia suspensa (Thunb.) Vahl [Oleaceae], Sophora flavescens Aiton	Decoction	N	N

				[Fabaceae], Glycyrrhiza uralensis Fisch. ex DC. [Fabaceae]			
Wang (2015c)	Danggui-yinzi granule	Commercial supplier Guangdong Yifang Chinese Herbal Pieces Co., LTD.	*Angelicae sinensis radix, Paeonia lactiflora, Cnidii rhizoma, Rehmanniae radix recens, Tribuli fructus, Polygoni multiflori radix, Astragali radix, Saposhnikoviae radix, Schizonepetae spica, Glycyrrhizae radix et rhizoma*	Angelica sinensis (Oliv.) Diels [Apiaceae], Paeonia lactiflora Pall. [Paeoniaceae](Paeonia lactiflora Alba), Conioselinum anthriscoides 'Chuanxiong' [Apiaceae], Rehmannia glutinosa (Gaertn.) DC. [Orobanchaceae](Rehmanniae Radix Recens), Tribulus terrestris L. [Zygophyllaceae], Reynoutria multiflora (Thunb.) Moldenke [Polygonaceae], Astragalus mongholicus Bunge [Fabaceae],	Granule	N	N

				Saposhnikovia divaricata (Turcz. ex Ledeb.) Schischk. [Apiaceae], Nepeta tenuifolia Benth. [Lamiaceae], Glycyrrhiza uralensis Fisch. ex DC. [Fabaceae]			
Yuan (2015)	Qingrexiaoyin decoction	Prepared by Researchers	*Lonicerae flos, Smilax glabrae rhizoma, Hedyotidis herba, Scutellariae barbatae herba, Sophorae tonkinensis radix et rhizoma, Lithospermi radix, Rubiae radix, Ecliptae herba, Sophorae flos, Zaocys, Dictamni radicis cortex, Sophorae radix, Cicadidae periostracum,*	Lonicera japonica Thunb. [Caprifoliaceae], Smilax glabra Roxb. [Smilacaceae], Scleromitrion diffusum (Willd.) R.J.Wang [Rubiaceae], Scutellaria barbata D.Don [Lamiaceae], Sophora tonkinensis Gagnep. [Fabaceae], Arnebia euchroma (Royle ex Benth.) I.M.Johnst. [Boraginaceae], Rubia tinctorum L.	Decoction	N	N

			Glycyrrhizae radix et rhizoma	[Rubiaceae], Eclipta prostrata (L.) L. [Asteraceae], Styphnolobium japonicum (L.) Schott [Fabaceae], Zaocys dhumnades Cantor [Colubridae], Dictamnus dasycarpus Turcz. [Rutaceae], Sophora flavescens Aiton [Fabaceae], Cryptotympana dubia (Haupt) [Cicadidae], Glycyrrhiza uralensis Fisch. ex DC. [Fabaceae]			
Zhang (2015a)	Liangxuerunfu decoction	Prepared by Researchers	*Smilax glabrae rhizoma, Isatidis folium, Rehmanniae radix recens, Salviae miltiorrhizae radix, Imperatae rhizoma, Paeoniae*	Smilax glabra Roxb. [Smilacaceae], Isatis tinctoria subsp. tinctoria [Brassicaceae], Rehmannia glutinosa (Gaertn.) DC. [Orobanchaceae](Reh	Decoction	N	N

radix rubra, Dictamni radicis cortex, Saposhnikoviae radix, Tribuli fructus, Sophorae flos, Solani nigri herba, Moutan radicis cortex, Glycyrrhizae radix et rhizoma	manniae Radix Recens), Salvia miltiorrhiza Bunge [Lamiaceae], Imperata cylindrica (L.) Raeusch. [Poaceae], Paeonia anomala subsp. veitchii (Lynch) D.Y.Hong & K.Y.Pan [Paeoniaceae], Dictamnus dasycarpus Turcz. [Rutaceae], Saposhnikovia divaricata (Turcz. ex Ledeb.) Schischk. [Apiaceae], Tribulus terrestris L. [Zygophyllaceae], Styphnolobium japonicum (L.) Schott [Fabaceae], Solanum nigrum L. [Solanaceae], Paeonia × suffruticosa Andrews

				[Paeoniaceae], Glycyrrhiza uralensis Fisch. ex DC. [Fabaceae]			
Zhang (2015b)	Yinxie capsule	Commercial supplier Xinjiang Weiatang Pharmaceutical Co., LTD.	*Smilax glabrae rhizoma, Smilacis chinae rhizoma*	Smilax glabra Roxb. [Smilacaceae], Smilax china L. [Smilacaceae]	Capsule	N	N
Zhang (2015c)	Zinyinqingrexiaofengsan	Prepared by Researchers	*Rehmanniae radix recens, Moutan radicis cortex, Liriopis seu ophiopogonis yuber, Scrophulariae radix, Salviae miltiorrhizae radix, Cannabis semen, Isatidis folium, Sophorae tonkinensis radix et rhizoma, Dictamni radicis cortex*	Rehmannia glutinosa (Gaertn.) DC. [Orobanchaceae](Rehmanniae Radix Recens), Paeonia × suffruticosa Andrews [Paeoniaceae], Ophiopogon japonicus (Thunb.) Ker Gawl. [Asparagaceae], Scrophularia ningpoensis Hemsl. [Scrophulariaceae], Salvia miltiorrhiza Bunge [Lamiaceae],	Decoction	N	N

				Cannabis sativa L. [Cannabaceae], Isatis tinctoria subsp. tinctoria [Brassicaceae], Sophora tonkinensis Gagnep. [Fabaceae], Dictamnus dasycarpus Turcz. [Rutaceae]			
Chen (2016)	Researcher prescription	Prepared by Researchers	*Persicae semen, Curcumae rhizoma, Sparganii rhizoma, Euonymi ramuli suberalatum , Spatholobi caulis, Citri nnshius pericarpium, Carthami flos, Prunellae spica, Coicis semen*	Prunus persica (L.) Batsch [Rosaceae], Curcuma phaeocaulis Valeton [Zingiberaceae], Sparganium stoloniferum (Buch.-Ham. ex Graebn.) Buch.-Ham. ex Juz. [Typhaceae], Euonymus alatus (Thunb.) Siebold [Celastraceae], Spatholobus suberectus Dunn [Fabaceae], Citrus × aurantium f. deliciosa	Decoction	N	N

				(Ten.) M.Hiroe [Rutaceae], Carthamus tinctorius L. [Asteraceae], Prunella vulgaris L. [Lamiaceae], Coix lacryma-jobi var. ma-yuen (Rom.Caill.) Stapf [Poaceae]			
He (2016a)	Liangxuerunfu decoction	Prepared by Researchers	*Smilax glabrae rhizoma, Isatidis folium, Rehmanniae radix recens, Salviae miltiorrhizae radix, Imperatae rhizoma, Paeoniae radix rubra, Dictamni radicis cortex, Saposhnikoviae radix, Tribuli fructus, Sophorae flos, Solani nigri herba, Moutan radicis cortex, Glycyrrhizae*	Smilax glabra Roxb. [Smilacaceae], Isatis tinctoria subsp. tinctoria [Brassicaceae], Rehmannia glutinosa (Gaertn.) DC. [Orobanchaceae](Rehmanniae Radix Recens), Salvia miltiorrhiza Bunge [Lamiaceae], Imperata cylindrica (L.) Raeusch. [Poaceae], Paeonia anomala subsp. veitchii (Lynch) D.Y.Hong & K.Y.Pan	Decoction	N	N

			radix et rhizoma	[Paeoniaceae], Dictamnus dasycarpus Turcz. [Rutaceae], Saposhnikovia divaricata (Turcz. ex Ledeb.) Schischk. [Apiaceae], Tribulus terrestris L. [Zygophyllaceae], Styphnolobium japonicum (L.) Schott [Fabaceae], Solanum nigrum L. [Solanaceae], Paeonia × suffruticosa Andrews [Paeoniaceae], Glycyrrhiza uralensis Fisch. ex DC. [Fabaceae]			
He (2016b)	Compound Qingdai pill	Commercial supplier Shaanxi Tianning Pharmaceutical Co.,	*Indigo pulverata levis, Mume fructus, Taraxaci herba, Lithospermi radix, Angelicae*	Persicaria tinctoria (Aiton) Spach [Polygonaceae], Prunus mume (Siebold) Siebold & Zucc. [Rosaceae],	Pill	N	N

	LTD.	*dahuricae radix, Salviae miltiorrhizae radix, Dictamni radicis cortex, Massa medicata fermentata, Dryopteridis crassirhizomatis rhizoma, Smilax glabrae rhizoma, Portulacae herba, Dioscorea hypoglauca, Crataegi fructus, Schisandrae fructus*	Taraxacum mongolicum Hand.-Mazz. [Asteraceae], Arnebia euchroma (Royle ex Benth.) I.M.Johnst. [Boraginaceae], Angelica dahurica (Hoffm.) Benth. & Hook.f. ex Franch. & Sav. [Apiaceae], Salvia miltiorrhiza Bunge [Lamiaceae], Dictamnus dasycarpus Turcz. [Rutaceae], Massa Medicata Fermentata, Dryopteris crassirhizoma Nakai [Polypodiaceae], Smilax glabra Roxb. [Smilacaceae], Portulaca oleracea L. [Portulacaceae], Dioscorea collettii var. hypoglauca (Palib.) S.J.Pei &

				C.T.Ting [Dioscoreaceae], Crataegus pinnatifida var. pinnatifida [Rosaceae], Schisandra chinensis (Turcz.) Baill. [Schisandraceae]			
Jiang (2016)	Yinxiping pill	Prepared by Researchers	*Rehmanniae radix recens, Hedyotidis herba, Scutellariae barbatae herba, Isatidis folium, Paeoniae radix rubra, Moutan radicis Ccortex, Salviae miltiorrhizae radix, Lithospermi radix, Ligustri fructus, Ecliptae herba, Liriopis seu ophiopogonis tuber, Dioscoreae rhizoma,*	Rehmannia glutinosa (Gaertn.) DC. [Orobanchaceae](Rehmanniae Radix Recens), Scleromitrion diffusum (Willd.) R.J.Wang [Rubiaceae], Scutellaria barbata D.Don [Lamiaceae], Isatis tinctoria subsp. tinctoria [Brassicaceae], Paeonia anomala subsp. veitchii (Lynch) D.Y.Hong & K.Y.Pan [Paeoniaceae],	Pill	N	N

			Dictamni radicis cortex, Glycyrrhizae radix et rhizoma	Paeonia × suffruticosa Andrews [Paeoniaceae], Salvia miltiorrhiza Bunge [Lamiaceae], Arnebia euchroma (Royle ex Benth.) I.M.Johnst. [Boraginaceae], Ligustrum lucidum W.T.Aiton [Oleaceae], Eclipta prostrata (L.) L. [Asteraceae], Ophiopogon japonicus (Thunb.) Ker Gawl. [Asparagaceae], Dioscorea japonica Thunb. [Dioscoreaceae], Dictamnus dasycarpus Turcz. [Rutaceae], Glycyrrhiza uralensis Fisch. ex DC. [Fabaceae]			
Shan (2016)	Compound glycyrrhizin	Commercial supplier	*Glycyrrhizae radix et rhizoma*	Glycyrrhiza uralensis Fisch. ex DC.	Liquid	N	N

		Fujian Mindong Lijixun Pharmaceutical Co., LTD.		[Fabaceae]			
Wang (2016a)	Xiaoyin granule	Commercial supplier Shaanxi Kanghui Pharmaceutical Co., Ltd.	*Rehmanniae radix recens, Moutan radicis cortex, Paeoniae radix rubra, Angelicae sinensis radix, Sophorae radix, Lonicerae flos, Scrophulariae radix, Arctii fructus, Cicadidae periostracum, Dictamni radicis cortex, Isatidis folium, Carthami flos, Saposhnikoviae radix*	Rehmannia glutinosa (Gaertn.) DC. [Orobanchaceae](Rehmanniae Radix Recens), Paeonia × suffruticosa Andrews [Paeoniaceae], Paeonia anomala subsp. veitchii (Lynch) D.Y.Hong & K.Y.Pan [Paeoniaceae], Angelica sinensis (Oliv.) Diels [Apiaceae], Sophora flavescens Aiton [Fabaceae], Lonicera japonica Thunb. [Caprifoliaceae], Scrophularia ningpoensis Hemsl.	Granule	N	N

				[Scrophulariaceae], Arctium lappa L. [Asteraceae], Cryptotympana dubia (Haupt) [Cicadidae], Dictamnus dasycarpus Turcz. [Rutaceae], Isatis tinctoria subsp. tinctoria [Brassicaceae], Carthamus tinctorius L. [Asteraceae], Saposhnikovia divaricata (Turcz. ex Ledeb.) Schischk. [Apiaceae]			
Wang (2016b)	Piminxiao capsule	Commercial supplier Shenyang Aohua Pharmaceutical Co., Ltd.	*Sophorae radix, Atractylodis rhizoma, Saposhnikoviae radix, Schizonepetae spica, Tribuli fructus, Dictamni radicis cortex,*	Sophora flavescens Aiton [Fabaceae], Atractylodes lancea (Thunb.) DC. [Asteraceae](Atractylodis Rhizoma), Saposhnikovia divaricata (Turcz. ex Ledeb.) Schischk.	Capsule	N	N

	Cnidi fructus, Xanthii fructus, Scolopendra, Indigo pulverata levis, Taraxaci herba, Violae herba, Scutellariae radix, Phellodendri cortex, Coptis chinensis, Cicadidae periostracum, Rehmanniae radix recens , Moutan radicis cortex, Chinese tamarisk twing, Lithospermi radix, Lycii radicis cortex	[Apiaceae], Nepeta tenuifolia Benth. [Lamiaceae], Tribulus terrestris L. [Zygophyllaceae], Dictamnus dasycarpus Turcz. [Rutaceae], Cnidium monnieri (L.) Cusson [Apiaceae], Xanthium strumarium L. [Asteraceae], Scolopendra subspinipes mutilans Linné Koch [Scolopendridae], Persicaria tinctoria (Aiton) Spach [Polygonaceae], Taraxacum mongolicum Hand.-Mazz. [Asteraceae], Viola mandshurica W.Becker [Violaceae], Scutellaria baicalensis Georgi [Lamiaceae], Phellodendron	

				chinense C.K.Schneid. [Rutaceae], Coptis chinensis Franch. [Ranunculaceae], Cryptotympana dubia (Haupt) [Cicadidae], Rehmannia glutinosa (Gaertn.) DC. [Orobanchaceae](Rehmanniae Radix Recens) , Paeonia × suffruticosa Andrews [Paeoniaceae], Tamarix chinensis Lour. [Tamaricaceae], Arnebia euchroma (Royle ex Benth.) I.M.Johnst. [Boraginaceae], Lycium barbarum L. [Solanaceae]			
Wu (2016)	Xiaoyin granule	Commercial supplier Shaanxi Kanghui	*Rehmanniae radix recens, Moutan radicis cortex, Paeoniae radix*	Rehmannia glutinosa (Gaertn.) DC. [Orobanchaceae](Rehmanniae Radix	Granule	N	N

Pharmaceutical Co., Ltd.	*rubra, Angelicae sinensis radix, Sophorae radix, Lonicerae flos, Scrophulariae radix, Arctii fructus, Cicadidae periostracum, Dictamni radicis cortex, Isatidis folium, Carthami flos, Saposhnikoviae radix*	Recens), Paeonia × suffruticosa Andrews [Paeoniaceae], Paeonia anomala subsp. veitchii (Lynch) D.Y.Hong & K.Y.Pan [Paeoniaceae], Angelica sinensis (Oliv.) Diels [Apiaceae], Sophora flavescens Aiton [Fabaceae], Lonicera japonica Thunb. [Caprifoliaceae], Scrophularia ningpoensis Hemsl. [Scrophulariaceae], Arctium lappa L. [Asteraceae], Cryptotympana dubia (Haupt) [Cicadidae], Dictamnus dasycarpus Turcz. [Rutaceae], Isatis tinctoria subsp.

				tinctoria [Brassicaceae], Carthamus tinctorius L. [Asteraceae], Saposhnikovia divaricata (Turcz. ex Ledeb.) Schischk. [Apiaceae]			
Xie (2016)	Xiaoyin granule	Commercial supplier Shaanxi Kanghui Pharmaceutical Co., Ltd.	*Rehmanniae radix recens, Moutan radicis cortex, Paeoniae radix rubra, Angelicae sinensis radix, Sophorae radix, Lonicerae flos, Scrophulariae radix, Arctii fructus, Cicadidae periostracum, Dictamni radicis cortex, Isatidis folium, Carthami flos, Saposhnikoviae radix*	Rehmannia glutinosa (Gaertn.) DC. [Orobanchaceae](Rehmanniae Radix Recens), Paeonia × suffruticosa Andrews [Paeoniaceae], Paeonia anomala subsp. veitchii (Lynch) D.Y.Hong & K.Y.Pan [Paeoniaceae], Angelica sinensis (Oliv.) Diels [Apiaceae], Sophora flavescens Aiton [Fabaceae], Lonicera japonica Thunb. [Caprifoliaceae],	Granule	N	N

				Scrophularia ningpoensis Hemsl. [Scrophulariaceae], Arctium lappa L. [Asteraceae], Cryptotympana dubia (Haupt) [Cicadidae], Dictamnus dasycarpus Turcz. [Rutaceae], Isatis tinctoria subsp. tinctoria [Brassicaceae], Carthamus tinctorius L. [Asteraceae], Saposhnikovia divaricata (Turcz. ex Ledeb.) Schischk. [Apiaceae]			
Xu (2016)	Compound glycyrrhizin	Commercial supplier Fujian Mindong Lijixun Pharmaceutical Co.,	*Glycyrrhizae radix et rhizoma*	Glycyrrhiza uralensis Fisch. ex DC. [Fabaceae]	Liquid	N	N

		LTD.					
Yang (2016a)	Qinmei granule	Prepared by Researchers	*Scutellariae radix, Indigo pulverata levis, Angelicae sinensis radix, Astragali radix, Mume fructus*	Scutellaria baicalensis Georgi [Lamiaceae], Persicaria tinctoria (Aiton) Spach [Polygonaceae], Angelica sinensis (Oliv.) Diels [Apiaceae], Astragalus mongholicus Bunge [Fabaceae], Prunus mume (Siebold) Siebold & Zucc. [Rosaceae]	Granule	N	N
Yang (2016b)	Qingreliangxue decoction	Prepared by Researchers	*Bubalus bubalis, Rehmanniae radix recens, Lithospermi radix, Moutan radicis cortex, Sophorae flos, Isatidis folium, Smilax glabrae fhizoma, Scutellariae radix, Rhei radix et*	Bubalus bubalis Linné [Bovidae], Rehmannia glutinosa (Gaertn.) DC. [Orobanchaceae](Rehmanniae Radix Recens), Arnebia euchroma (Royle ex Benth.) I.M.Johnst. [Boraginaceae], Paeonia × suffruticosa Andrews	Decoction	N	N

			rhizoma, Curcumae radix, Vespae nidus	[Paeoniaceae], Styphnolobium japonicum (L.) Schott [Fabaceae], Isatis tinctoria subsp. tinctoria [Brassicaceae], Smilax glabra Roxb. [Smilacaceae], Scutellaria baicalensis Georgi [Lamiaceae], Rheum officinale Baill. [Polygonaceae], Curcuma phaeocaulis Valeton [Zingiberaceae], Vespae Nidus			
Yu (2016)	Qingreliangxue decoction	Prepared by Researchers	*Bubalus bubalis, Rehmanniae radix recens, Lithospermi radix, Moutan radicis cortex, Sophorae flos, Isatidis folium, Smilax glabrae rhizoma,*	Bubalus bubalis Linné [Bovidae], Rehmannia glutinosa (Gaertn.) DC. [Orobanchaceae](Rehmanniae Radix Recens), Arnebia euchroma (Royle ex Benth.) I.M.Johnst. [Boraginaceae],	Decoction	N	N

			Scutellariae adixm, Rhei radix et rhizoma, Curcumae radix, Vespae nidus	Paeonia × suffruticosa Andrews [Paeoniaceae], Styphnolobium japonicum (L.) Schott [Fabaceae], Isatis tinctoria subsp. tinctoria [Brassicaceae], Smilax glabra Roxb. [Smilacaceae], Scutellaria baicalensis Georgi [Lamiaceae], Rheum officinale Baill. [Polygonaceae], Curcuma phaeocaulis Valeton [Zingiberaceae], Vespae Nidus			
Cao (2017)	Compound glycyrrhizin	Commercial supplier Henan Xin Shuaike Pharmaceutical Co., LTD.	*Glycyrrhizae radix et rhizoma*	Glycyrrhiza uralensis Fisch. ex DC. [Fabaceae]	Tablet	N	N
Cheng	Compound	Commerci	*Glycyrrhizae*	Glycyrrhiza uralensis	Tablet	N	N

(2017)	glycyrrhizin	al supplier Henan Xin Shuaike Pharmaceutical Co., LTD.	*radix et rhizoma*	Fisch. ex DC. [Fabaceae]			
Ding (2017)	Ziyinhuoxuerunzao decoction	Prepared by Researchers	*Testudinis chinemis plastrum et carapax, Ostreae testa, Rehmanniae radix recens , Scrophulariae radix, Asparagi tuber, Liriopis seu ophiopogonis tuber, Polygoni multiflori radix, Spatholobi caulis, Paeoniae radix rubra , Prunellae spica, Hedyotidis herba, Curcumae rhizoma, Trichosanthes kirilowii,*	Chinemys reevesii Gray [Emydidae], Ostrea gigas Thunberg [Ostreidae], Rehmannia glutinosa (Gaertn.) DC. [Orobanchaceae](Rehmanniae Radix Recens) , Scrophularia ningpoensis Hemsl. [Scrophulariaceae], Asparagus cochinchinensis (Lour.) Merr. [Asparagaceae], Ophiopogon japonicus (Thunb.) Ker Gawl. [Asparagaceae],	Decoction	N	N

	Forsythiae fructus, Glycyrrhizae radix et rhizoma	Reynoutria multiflora (Thunb.) Moldenke [Polygonaceae], Spatholobus suberectus Dunn [Fabaceae], Paeonia anomala subsp. veitchii (Lynch) D.Y.Hong & K.Y.Pan [Paeoniaceae] , Prunella vulgaris L. [Lamiaceae], Scleromitrion diffusum (Willd.) R.J.Wang [Rubiaceae], Curcuma phaeocaulis Valeton [Zingiberaceae], Trichosanthes kirilowii Maxim. [Cucurbitaceae], Forsythia suspensa (Thunb.) Vahl [Oleaceae], Glycyrrhiza uralensis Fisch. ex DC.	

				[Fabaceae]			
Du (2017)	Total Glycosides of Paeoniae Alba capsule	Commercial supplier Ningbo Lihua Pharmaceutical Co., LTD.	*Paeonia lactiflora*	Paeonia lactiflora Pall. [Paeoniaceae](Paeonia lactiflora Alba)	Capsule	N	N
Feng (2017)	Yinxiping pill	Prepared by Researchers	*Rehmanniae radix recens, Hedyotidis herba, Scutellariae barbatae herba, Isatidis folium, Paeoniae radix rubra, Moutan radicis cortex, Salviae miltiorrhizae radix, Lithospermi radix, Ligustri fructus, Ecliptae herba, Liriopis seu ophiopogonis tuber, Dioscoreae rhizoma,*	Rehmannia glutinosa (Gaertn.) DC. [Orobanchaceae](Rehmanniae Radix Recens), Scleromitrion diffusum (Willd.) R.J.Wang [Rubiaceae], Scutellaria barbata D.Don [Lamiaceae], Isatis tinctoria subsp. tinctoria [Brassicaceae], Paeonia anomala subsp. veitchii (Lynch) D.Y.Hong & K.Y.Pan [Paeoniaceae],	Pill	N	N

			Dictamni radicis cortex, Glycyrrhizae radix et rhizoma	Paeonia × suffruticosa Andrews [Paeoniaceae], Salvia miltiorrhiza Bunge [Lamiaceae], Arnebia euchroma (Royle ex Benth.) I.M.Johnst. [Boraginaceae], Ligustrum lucidum W.T.Aiton [Oleaceae], Eclipta prostrata (L.) L. [Asteraceae], Ophiopogon japonicus (Thunb.) Ker Gawl. [Asparagaceae], Dioscorea japonica Thunb. [Dioscoreaceae], Dictamnus dasycarpus Turcz. [Rutaceae], Glycyrrhiza uralensis Fisch. ex DC. [Fabaceae]			
Han (2017a)	Xiaoyin granule	Commercial supplier	*Rehmanniae radix recens, Moutan*	Rehmannia glutinosa (Gaertn.) DC.	Granule	N	N

	Shaanxi Kanghui Pharmaceutical Co., Ltd.	*radicis cortex, Paeoniae radix rubra, Angelicae sinensis radix, Sophorae radix, Lonicerae flos, Scrophulariae radix, Arctii fructus, Cicadidae periostracum, Dictamni radicis cortex, Isatidis folium, Carthami flos, Saposhnikoviae radix*	[Orobanchaceae](Rehmanniae Radix Recens), Paeonia × suffruticosa Andrews [Paeoniaceae], Paeonia anomala subsp. veitchii (Lynch) D.Y.Hong & K.Y.Pan [Paeoniaceae], Angelica sinensis (Oliv.) Diels [Apiaceae], Sophora flavescens Aiton [Fabaceae], Lonicera japonica Thunb. [Caprifoliaceae], Scrophularia ningpoensis Hemsl. [Scrophulariaceae], Arctium lappa L. [Asteraceae], Cryptotympana dubia (Haupt) [Cicadidae], Dictamnus dasycarpus Turcz.	

				[Rutaceae], Isatis tinctoria subsp. tinctoria [Brassicaceae], Carthamus tinctorius L. [Asteraceae], Saposhnikovia divaricata (Turcz. ex Ledeb.) Schischk. [Apiaceae]			
Han (2017b)	Compound Qingdai capsule	Commercial supplier Shaanxi Tianning Pharmaceutical Co., Ltd.	*Indigo pulverata levis, Mume fructus, Taraxaci herba, Lithospermi radix, Angelicae dahuricae radix, Salviae miltiorrhizae Radix, Dictamni radicis cortex, Massa medicata fermentata, Dryopteridis crassirhizomatis rhizoma, Smilax glabrae rhizoma,*	Persicaria tinctoria (Aiton) Spach [Polygonaceae], Prunus mume (Siebold) Siebold & Zucc. [Rosaceae], Taraxacum mongolicum Hand.-Mazz. [Asteraceae], Arnebia euchroma (Royle ex Benth.) I.M.Johnst. [Boraginaceae], Angelica dahurica (Hoffm.) Benth. & Hook.f. ex Franch. & Sav. [Apiaceae],	Capsule	N	N

			Portulacae herba, Dioscorea hypoglauca, Crataegi fructus, Schisandrae fructus	Salvia miltiorrhiza Bunge [Lamiaceae], Dictamnus dasycarpus Turcz. [Rutaceae], Massa Medicata Fermentata, Dryopteris crassirhizoma Nakai [Polypodiaceae], Smilax glabra Roxb. [Smilacaceae], Portulaca oleracea L. [Portulacaceae], Dioscorea collettii var. hypoglauca (Palib.) S.J.Pei & C.T.Ting [Dioscoreaceae], Crataegus pinnatifida var. pinnatifida [Rosaceae], Schisandra chinensis (Turcz.) Baill. [Schisandraceae]			
Li (2017)	Qingrejiedu decoction	Prepared by Researcher	*Smilax glabrae rhizoma, Rehmanniae radix*	Smilax glabra Roxb. [Smilacaceae], Rehmannia glutinosa	Decoction	N	N

s	*recens , Scrophulariae radix, Isatidis folium, Isatidis radix, Scutellariae radix, Lonicerae flos, Forsythiae fructus , Carthami flos, Sophorae flos, Moutan radicis cortex, Angelicae sinensis radix, Liriopis seu ophiopogonis tuber, Glycyrrhizae radix et rhizoma*	(Gaertn.) DC. [Orobanchaceae](Rehmanniae Radix Recens), Scrophularia ningpoensis Hemsl. [Scrophulariaceae], Isatis tinctoria subsp. tinctoria [Brassicaceae], Isatis tinctoria subsp. tinctoria [Brassicaceae](Isatidis Radix), Scutellaria baicalensis Georgi [Lamiaceae], Lonicera japonica Thunb. [Caprifoliaceae], Forsythia suspensa (Thunb.) Vahl [Oleaceae] , Carthamus tinctorius L. [Asteraceae], Styphnolobium japonicum (L.) Schott [Fabaceae], Paeonia × suffruticosa Andrews [Paeoniaceae],	

				Angelica sinensis (Oliv.) Diels [Apiaceae], Ophiopogon japonicus (Thunb.) Ker Gawl. [Asparagaceae], Glycyrrhiza uralensis Fisch. ex DC. [Fabaceae]			
Liu (2017)	Yinxie capsule	Commercial supplier Xinjiang Wei-A-Tang Pharmaceutical Co., Ltd.	*Smilax glabrae rhizoma, Smilacis chinae rhizoma*	Smilax glabra Roxb. [Smilacaceae], Smilax china L. [Smilacaceae]	Capsule	N	N
Luo (2017)	Compound glycyrrhizin	Commercial supplier Henan Xin Shuaike Pharmaceutical Co., LTD.	*Glycyrrhizae radix et rhizoma*	Glycyrrhiza uralensis Fisch. ex DC. [Fabaceae]	Tablet	N	N
Pang (2017)	Compound Qingdai	Commercial supplier	*Indigo pulverata levis, Mume*	Persicaria tinctoria (Aiton) Spach	Capsule	N	N

	capsule	Shaanxi Pharmaceutical Holdings Group Tianning Pharmaceutical Co., LTD.	*fructus, Taraxaci herba, Lithospermi radix, Angelicae dahuricae radix, Salviae miltiorrhizae radix, Dictamni radicis cortex, Massa medicata fermentata, Dryopteridis crassirhizomatis rhizoma, Smilax glabrae rhizoma, Portulacae herba, Dioscorea hypoglauca, Crataegi fructus, Schisandrae fructus*	[Polygonaceae], Prunus mume (Siebold) Siebold & Zucc. [Rosaceae], Taraxacum mongolicum Hand.-Mazz. [Asteraceae], Arnebia euchroma (Royle ex Benth.) I.M.Johnst. [Boraginaceae], Angelica dahurica (Hoffm.) Benth. & Hook.f. ex Franch. & Sav. [Apiaceae], Salvia miltiorrhiza Bunge [Lamiaceae], Dictamnus dasycarpus Turcz. [Rutaceae], Massa Medicata Fermentata, Dryopteris crassirhizoma Nakai [Polypodiaceae], Smilax glabra Roxb. [Smilacaceae], Portulaca oleracea L.

				[Portulacaceae], Dioscorea collettii var. hypoglauca (Palib.) S.J.Pei & C.T.Ting [Dioscoreaceae], Crataegus pinnatifida var. pinnatifida [Rosaceae], Schisandra chinensis (Turcz.) Baill. [Schisandraceae]			
Shi (2017)	Liangxuexiao feng decoction	Prepared by Researcher s	*Rehmanniae radix recens, Paeonia lactiflora, Scrophulariae radix, Anemarrhenae rhizoma, Gypsum fibrosum, Imperatae rhizoma, Saposhnikoviae radix, Schizonepetae spica, Cimicifugae rhizoma,*	Rehmannia glutinosa (Gaertn.) DC. [Orobanchaceae](Reh manniae Radix Recens), Paeonia lactiflora Pall. [Paeoniaceae](Paeoni a lactiflora Alba), Scrophularia ningpoensis Hemsl. [Scrophulariaceae], Anemarrhena asphodeloides Bunge [Asparagaceae], Gypsum Fibrosum,	Decoction	N	N

			Glycyrrhizae radix et rhizoma , Arctii fructus, Lonicerae flos	Imperata cylindrica (L.) Raeusch. [Poaceae], Saposhnikovia divaricata (Turcz. ex Ledeb.) Schischk. [Apiaceae], Nepeta tenuifolia Benth. [Lamiaceae], Actaea heracleifolia (Kom.) J.Compton [Ranunculaceae], Glycyrrhiza uralensis Fisch. ex DC. [Fabaceae] . Arctium lappa L. [Asteraceae], Lonicera japonica Thunb. [Caprifoliaceae]			
Song (2017)	Yangxuetongluo decoction	Prepared by Researchers	*Smilax glabrae rhizoma, Sophorae flos, Angelicae sinensis radix, Spatholobi caulis, Saposhnikoviae radix,*	Smilax glabra Roxb. [Smilacaceae], Styphnolobium japonicum (L.) Schott [Fabaceae], Angelica sinensis (Oliv.) Diels [Apiaceae], Spatholobus	Decoction	N	N

			Rehmanniae radix recens, Liriopis seu ophiopogonis tuber	suberectus Dunn [Fabaceae], Saposhnikovia divaricata (Turcz. ex Ledeb.) Schischk. [Apiaceae], Rehmannia glutinosa (Gaertn.) DC. [Orobanchaceae](Rehmanniae Radix Recens), Ophiopogon japonicus (Thunb.) Ker Gawl. [Asparagaceae]			
Wang (2017)	Yinxie capsule	Commercial supplier Xinjiang Wei-A-Tang Pharmaceutical Co., Ltd.	*Smilax glabrae rhizoma, Smilacis chinae rhizoma*	Smilax glabra Roxb. [Smilacaceae], Smilax china L. [Smilacaceae]	Capsule	N	N
Wu (2017)	Shentongzhuyu decoction	Prepared by Researchers	*Persicae semen, Carthami flos , Cnidii rhizoma, Gentianae macrophyllae*	Prunus persica (L.) Batsch [Rosaceae], Carthamus tinctorius L. [Asteraceae], Conioselinum	Decoction	N	N

	radix, Osterici seu notopterygii radix et rhizoma, Achyranthis radix, Lumbricus, Angelicae sinensis radix, Commiphora myrrha, Trogopterorum faeces, Cyperi rhizoma, Glycyrrhizae radix et rhizoma	anthriscoides 'Chuanxiong' [Apiaceae], Gentiana macrophylla Pall. [Gentianaceae], Hansenia weberbaueriana (Fedde ex H.Wolff) Pimenov & Kljuykov [Apiaceae], Achyranthes bidentata Blume [Amaranthaceae], Pericaeta communisma Gate et Hatai [Lumbricidae], Angelica sinensis (Oliv.) Diels [Apiaceae], Commiphora myrrha (T.Nees) Engl. [Burseraceae], Trogopterorum Faeces, Cyperus rotundus L. [Cyperaceae], Glycyrrhiza uralensis	

				Fisch. ex DC. [Fabaceae]			
Yang (2017)	Xiaoyin granule	Commercial supplier Shaanxi Kanghui Pharmaceutical Co., Ltd.	*Rehmanniae radix recens, Moutan radicis cortex, Paeoniae radix rubra, Angelicae sinensis radix, Sophorae radix, Lonicerae flos, Scrophulariae radix, Arctii fructus, Cicadidae periostracum, Dictamni radicis cortex, Isatidis folium, Carthami flos, Saposhnikoviae radix*	Rehmannia glutinosa (Gaertn.) DC. [Orobanchaceae](Rehmanniae Radix Recens), Paeonia × suffruticosa Andrews [Paeoniaceae], Paeonia anomala subsp. veitchii (Lynch) D.Y.Hong & K.Y.Pan [Paeoniaceae], Angelica sinensis (Oliv.) Diels [Apiaceae], Sophora flavescens Aiton [Fabaceae], Lonicera japonica Thunb. [Caprifoliaceae], Scrophularia ningpoensis Hemsl. [Scrophulariaceae], Arctium lappa L. [Asteraceae], Cryptotympana	Granule	N	N

				dubia (Haupt) [Cicadidae], Dictamnus dasycarpus Turcz. [Rutaceae], Isatis tinctoria subsp. tinctoria [Brassicaceae], Carthamus tinctorius L. [Asteraceae], Saposhnikovia divaricata (Turcz. ex Ledeb.) Schischk. [Apiaceae]			
Zeng (2017)	Compound glycyrrhizin	Commercial supplier Beijing Kain Technology Co., Ltd.	*Glycyrrhizae radix et rhizoma*	Glycyrrhiza uralensis Fisch. ex DC. [Fabaceae]	Capsule	N	N
Zhang (2017a)	Taohongershao decoction	Prepared by Researchers	*Persicae semen, Carthami flos, Paeoniae radix rubra, Paeonia lactiflora, Sparganii rhizoma,*	Prunus persica (L.) Batsch [Rosaceae], Carthamus tinctorius L. [Asteraceae], Paeonia anomala subsp. veitchii (Lynch) D.Y.Hong &	Decoction	N	N

	Curcumae rhizoma, Salviae miltiorrhizae radix, Cnidii rhizoma, Euonymi ramuli suberalatum, Rhei radix et rhizoma, Spatholobi caulis, Rehmanniae radix recens, Sophorae flos, Glycyrrhizae radix et rhizoma	K.Y.Pan [Paeoniaceae], Paeonia lactiflora Pall. [Paeoniaceae](Paeonia lactiflora Alba), Sparganium stoloniferum (Buch.-Ham. ex Graebn.) Buch.-Ham. ex Juz. [Typhaceae], Curcuma phaeocaulis Valeton [Zingiberaceae], Salvia miltiorrhiza Bunge [Lamiaceae], Conioselinum anthriscoides 'Chuanxiong' [Apiaceae], Euonymus alatus (Thunb.) Siebold [Celastraceae], Rheum officinale Baill. [Polygonaceae], Spatholobus suberectus Dunn	

				[Fabaceae], Rehmannia glutinosa (Gaertn.) DC. [Orobanchaceae](Rehmanniae Radix Recens), Styphnolobium japonicum (L.) Schott [Fabaceae], Glycyrrhiza uralensis Fisch. ex DC. [Fabaceae]			
Zhang (2017b)	Taohongershao decoction	Prepared by Researchers	*Persicae semen, Carthami flos, Paeoniae radix rubra, Paeonia lactiflora, Sparganii rhizoma, Curcumae rhizoma, Salviae miltiorrhizae radix, Cnidii rhizoma, Euonymi ramuli suberalatum, Rhei radix et rhizoma,*	Prunus persica (L.) Batsch [Rosaceae], Carthamus tinctorius L. [Asteraceae], Paeonia anomala subsp. veitchii (Lynch) D.Y.Hong & K.Y.Pan [Paeoniaceae], Paeonia lactiflora Pall. [Paeoniaceae](Paeonia lactiflora Alba), Sparganium stoloniferum (Buch.-	Decoction	N	N

	Spatholobi caulis, Rehmanniae radix recens, Sophorae flos, Glycyrrhizae radix et rhizoma	Ham. ex Graebn.) Buch.-Ham. ex Juz. [Typhaceae], Curcuma phaeocaulis Valeton [Zingiberaceae], Salvia miltiorrhiza Bunge [Lamiaceae], Conioselinum anthriscoides 'Chuanxiong' [Apiaceae], Euonymus alatus (Thunb.) Siebold [Celastraceae], Rheum officinale Baill. [Polygonaceae], Spatholobus suberectus Dunn [Fabaceae], Rehmannia glutinosa (Gaertn.) DC. [Orobanchaceae](Rehmanniae Radix Recens), Styphnolobium japonicum (L.) Schott	

				[Fabaceae], Glycyrrhiza uralensis Fisch. ex DC. [Fabaceae]			
Zhang (2017c)	Taohongershao decoction	Prepared by Researchers	*Persicae semen, Carthami flos, Paeoniae radix rubra, Paeonia lactiflora, Sparganii rhizoma, Curcumae rhizoma, Salviae miltiorrhizae radix, Cnidii rhizoma, Euonymi ramuli suberalatum, Rhei radix et rhizoma, Spatholobi caulis, Rehmanniae radix recens, Sophorae flos, Glycyrrhizae radix et rhizoma*	Prunus persica (L.) Batsch [Rosaceae], Carthamus tinctorius L. [Asteraceae], Paeonia anomala subsp. veitchii (Lynch) D.Y.Hong & K.Y.Pan [Paeoniaceae], Paeonia lactiflora Pall. [Paeoniaceae](Paeonia lactiflora Alba), Sparganium stoloniferum (Buch.-Ham. ex Graebn.) Buch.-Ham. ex Juz. [Typhaceae], Curcuma phaeocaulis Valeton [Zingiberaceae], Salvia miltiorrhiza Bunge [Lamiaceae],	Decoction	N	N

				Conioselinum anthriscoides 'Chuanxiong' [Apiaceae], Euonymus alatus (Thunb.) Siebold [Celastraceae], Rheum officinale Baill. [Polygonaceae], Spatholobus suberectus Dunn [Fabaceae], Rehmannia glutinosa (Gaertn.) DC. [Orobanchaceae](Rehmanniae Radix Recens), Styphnolobium japonicum (L.) Schott [Fabaceae], Glycyrrhiza uralensis Fisch. ex DC. [Fabaceae]			
Zhang (2017d)	Liangxuexiaofeng	Prepared by Researchers	*Rehmanniae radix recens, Gypsum fibrosum, Bubalus bubalis,*	Rehmannia glutinosa (Gaertn.) DC. [Orobanchaceae](Rehmanniae Radix	Granule	N	N

	Scrophulariae radix, Imperatae rhizoma, Paeonia lactiflora, Anemarrhenae rhizoma, Lonicerae flos, Arctii fructus, Schizonepetae spica, Saposhnikoviae radix	Recens), Gypsum Fibrosum, Bubalus bubalis Linné [Bovidae], Scrophularia ningpoensis Hemsl. [Scrophulariaceae], Imperata cylindrica (L.) Raeusch. [Poaceae], Paeonia lactiflora Pall. [Paeoniaceae](Paeonia lactiflora Alba), Anemarrhena asphodeloides Bunge [Asparagaceae], Lonicera japonica Thunb. [Caprifoliaceae], Arctium lappa L. [Asteraceae], Nepeta tenuifolia Benth. [Lamiaceae], Saposhnikovia divaricata (Turcz. ex Ledeb.) Schischk. [Apiaceae]	

Zhang (2017e)	Taohongershao decoction	Prepared by Researchers	*Persicae semen, Carthami flos, Paeoniae radix rubra, Paeonia lactiflora, Sparganii rhizoma, Curcumae rhizoma, Salviae miltiorrhizae radix, Cnidii rhizoma, Euonymi ramuli suberalatum, Rhei radix et rhizoma, Spatholobi caulis, Rehmanniae radix recens, Sophorae flos, Glycyrrhizae radix et rhizoma*	Prunus persica (L.) Batsch [Rosaceae], Carthamus tinctorius L. [Asteraceae], Paeonia anomala subsp. veitchii (Lynch) D.Y.Hong & K.Y.Pan [Paeoniaceae], Paeonia lactiflora Pall. [Paeoniaceae](Paeonia lactiflora Alba), Sparganium stoloniferum (Buch.-Ham. ex Graebn.) Buch.-Ham. ex Juz. [Typhaceae], Curcuma phaeocaulis Valeton [Zingiberaceae], Salvia miltiorrhiza Bunge [Lamiaceae], Conioselinum anthriscoides 'Chuanxiong' [Apiaceae],	Decoction	N	N

				Euonymus alatus (Thunb.) Siebold [Celastraceae], Rheum officinale Baill. [Polygonaceae], Spatholobus suberectus Dunn [Fabaceae], Rehmannia glutinosa (Gaertn.) DC. [Orobanchaceae](Rehmanniae Radix Recens), Styphnolobium japonicum (L.) Schott [Fabaceae], Glycyrrhiza uralensis Fisch. ex DC. [Fabaceae]			
Zhao (2017)	Yinxie capsule	Commercial supplier Xinjiang Wei-A-Tang Pharmaceutical Co., Ltd.	*Smilax glabrae rhizoma, Smilacis chinae rhizoma*	Smilax glabra Roxb. [Smilacaceae], Smilax china L. [Smilacaceae]	Capsule	N	N

Chai (2018)	Xiaoyin decoction	Prepared by Researchers	*Salviae miltiorrhizae radix, Spatholobi caulis, Sophorae flos, Lithospermi radix, Rehmanniae radix recens, Rehmanniae radix preparata, Imperatae rhizoma, Moutan radicis cortex, Angelicae sinensis radix, Glycyrrhizae radix et rhizoma*	Salvia miltiorrhiza Bunge [Lamiaceae], Spatholobus suberectus Dunn [Fabaceae], Styphnolobium japonicum (L.) Schott [Fabaceae], Arnebia euchroma (Royle ex Benth.) I.M.Johnst. [Boraginaceae], Rehmannia glutinosa (Gaertn.) DC. [Orobanchaceae](Rehmanniae Radix Recens), Rehmannia glutinosa (Gaertn.) DC. [Orobanchaceae](Rehmanniae Radix Preparata), Imperata cylindrica (L.) Raeusch. [Poaceae], Paeonia × suffruticosa Andrews [Paeoniaceae], Angelica sinensis	Decoction	N	N

				(Oliv.) Diels [Apiaceae], Glycyrrhiza uralensis Fisch. ex DC. [Fabaceae]			
Li (2018)	Yinxie capsule	Commercial supplier Shaanxi Dongtai Manufacturing Pharmaceutical Co., Ltd.	*Smilax glabrae rhizoma, Smilacis chinae rhizoma*	Smilax glabra Roxb. [Smilacaceae], Smilax china L. [Smilacaceae]	Capsule	N	N
Liu (2018)	Banzhilian decoction	Prepared by Researchers	*Scutellariae barbatae herba, Lithospermi radix, Taraxaci herba, Chrysanthmi flos, Violae herba, Dioscorea hypoglauca, Schizonepetae spica, Saposhnikoviae radix, Cicadidae*	Scutellaria barbata D.Don [Lamiaceae], Arnebia euchroma (Royle ex Benth.) I.M.Johnst. [Boraginaceae], Taraxacum mongolicum Hand.-Mazz. [Asteraceae], Chrysanthemum × morifolium (Ramat.) Hemsl. [Asteraceae], Viola mandshurica	Decoction	N	N

			periostracum, Cnidi fructus, Dictamni radicis cortex, Kochiae fructus	W.Becker [Violaceae], Dioscorea collettii var. hypoglauca (Palib.) S.J.Pei & C.T.Ting [Dioscoreaceae], Nepeta tenuifolia Benth. [Lamiaceae], Saposhnikovia divaricata (Turcz. ex Ledeb.) Schischk. [Apiaceae], Cryptotympana dubia (Haupt) [Cicadidae], Cnidium monnieri (L.) Cusson [Apiaceae], Dictamnus dasycarpus Turcz. [Rutaceae], Bassia scoparia (L.) A.J.Scott [Amaranthaceae]			
Luo (2018)	Zicaohuoxue decoction	Prepared by Researcher s	*Lithospermi radix, Pinelliae tuber, Coptis chinensis, Eupolyphaga,*	Arnebia euchroma (Royle ex Benth.) I.M.Johnst. [Boraginaceae], Pinellia ternata	Decoction	N	N

	Cicadidae periostracum, Moutan radicis cortex, Indigo Pulverata levis citri Unshius pericarpium, Magnoliae cortex, Kochiae fructus, Dictamni radicis cortex, Angelicae sinensis radix, Lumbricus	(Thunb.) Makino [Araceae], Coptis chinensis Franch. [Ranunculaceae], Eupolyphaga sinensis Walker [Blattidae], Cryptotympana dubia (Haupt) [Cicadidae], Paeonia × suffruticosa Andrews [Paeoniaceae], Persicaria tinctoria (Aiton) Spach [Polygonaceae], Citrus × aurantium f. deliciosa (Ten.) M.Hiroe [Rutaceae], Magnolia officinalis Rehder & E.H.Wilson [Magnoliaceae], Bassia scoparia (L.) A.J.Scott [Amaranthaceae], Dictamnus dasycarpus Turcz. [Rutaceae], Angelica	

				sinensis (Oliv.) Diels [Apiaceae], Pericaeta communisma Gate et Hatai [Lumbricidae]			
Ma (2018a)	Xiaoyin granule	Commercial supplier Shaanxi Kanghui Pharmaceutical Co., Ltd.	*Rehmanniae radix recens, Moutan radicis cortex, Paeoniae radix rubra, Angelicae sinensis radix, Sophorae radix, Lonicerae flos, Scrophulariae radix, Arctii fructus, Cicadidae periostracum, Dictamni radicis cortex, Isatidis folium, Carthami flos, Saposhnikoviae radix*	Rehmannia glutinosa (Gaertn.) DC. [Orobanchaceae](Rehmanniae Radix Recens), Paeonia × suffruticosa Andrews [Paeoniaceae], Paeonia anomala subsp. veitchii (Lynch) D.Y.Hong & K.Y.Pan [Paeoniaceae], Angelica sinensis (Oliv.) Diels [Apiaceae], Sophora flavescens Aiton [Fabaceae], Lonicera japonica Thunb. [Caprifoliaceae], Scrophularia ningpoensis Hemsl. [Scrophulariaceae], Arctium lappa L.	Granule	N	N

				[Asteraceae], Cryptotympana dubia (Haupt) [Cicadidae], Dictamnus dasycarpus Turcz. [Rutaceae], Isatis tinctoria subsp. tinctoria [Brassicaceae], Carthamus tinctorius L. [Asteraceae], Saposhnikovia divaricata (Turcz. ex Ledeb.) Schischk. [Apiaceae]			
Ma (2018b)	Qingrequshi decoction	Prepared by Researchers	*Sophorae flos, Atractylodis rhizoma lba, Smilax glabrae rhizoma, Angelicae sinensis radix, Stephania tetrandra, Isatidis folium, Gentianae macrophyllae*	Styphnolobium japonicum (L.) Schott [Fabaceae], Atractylodes lancea (Thunb.) DC. [Asteraceae], Smilax glabra Roxb. [Smilacaceae], Angelica sinensis (Oliv.) Diels [Apiaceae], Stephania	Decoction	N	N

			radix, Scutellariae barbatae herba, Glycyrrhizae radix et rhizoma	tetrandra S.Moore [Menispermaceae], Isatis tinctoria subsp. tinctoria [Brassicaceae], Gentiana macrophylla Pall. [Gentianaceae], Scutellaria barbata D.Don [Lamiaceae], Glycyrrhiza uralensis Fisch. ex DC. [Fabaceae]			
Xiao (2018)	Xiaoyin decoction	Prepared by Researchers	*Rehmanniae radix recens, Smilax glabrae rhizoma, Lonicerae flos, Mume fructus, Lithospermi radix, Zaocys, Imperatae rhizoma, Spatholobi caulis, Ecliptae herba, Sophorae tonkinensis radix et rhizoma,*	Rehmannia glutinosa (Gaertn.) DC. [Orobanchaceae](Rehmanniae Radix Recens), Smilax glabra Roxb. [Smilacaceae], Lonicera japonica Thunb. [Caprifoliaceae], Prunus mume (Siebold) Siebold & Zucc. [Rosaceae], Arnebia euchroma	Decoction	N	N

			Sophorae flos, Sophorae radix, Glycyrrhizae radix et rhizoma	(Royle ex Benth.) I.M.Johnst. [Boraginaceae], Zaocys dhumnades Cantor [Colubridae], Imperata cylindrica (L.) Raeusch. [Poaceae], Spatholobus suberectus Dunn [Fabaceae], Eclipta prostrata (L.) L. [Asteraceae], Sophora tonkinensis Gagnep. [Fabaceae], Styphnolobium japonicum (L.) Schott [Fabaceae], Sophora flavescens Aiton [Fabaceae], Glycyrrhiza uralensis Fisch. ex DC. [Fabaceae]			
Xie (2018)	Qingying decoction	Commercial supplier Guangdong Yifang	*Bubalus bubalis, Rehmanniae radix recens, Lonicerae flos, Forsythiae*	Bubalus bubalis Linné [Bovidae], Rehmannia glutinosa (Gaertn.) DC.	Granule	N	N

	Pharmaceutical Co., Ltd.	*fructus, Scrophulariae radix, Salviae miltiorrhizae radix, Liriopis seu ophiopogonis tuber, Moutan radicis cortex, Dictamni radicis cortex, Astragali radix, Ganoderma lucidum, Glycyrrhizae radix et rhizoma*	[Orobanchaceae](Rehmanniae Radix Recens), Lonicera japonica Thunb. [Caprifoliaceae], Forsythia suspensa (Thunb.) Vahl [Oleaceae], Scrophularia ningpoensis Hemsl. [Scrophulariaceae]m, Salvia miltiorrhiza Bunge [Lamiaceae], Ophiopogon japonicus (Thunb.) Ker Gawl. [Asparagaceae], Paeonia × suffruticosa Andrews [Paeoniaceae], Dictamnus dasycarpus Turcz. [Rutaceae], Astragalus mongholicus Bunge [Fabaceae], Ganoderma lucidum	

				Karsten [Polyporaceae], Glycyrrhiza uralensis Fisch. ex DC. [Fabaceae]			
Zhang (2018a)	Compound glycyrrhizin	Commercial supplier Minophagen Pharmaceutical Co., Ltd.	*Glycyrrhizae radix et rhizoma*	Glycyrrhiza uralensis Fisch. ex DC. [Fabaceae]	Tablet	N	N
Zhang (2018b)	Xiaoyin granule	Commercial supplier Shaanxi Kanghui Pharmaceutical Co., Ltd.	*Rehmanniae radix recens, Moutan radicis cortex, Paeoniae radix rubra, Angelicae sinensis radix, Sophorae radix, Lonicerae flos, Scrophulariae radix, Arctii fructus, Cicadidae periostracum, Dictamni radicis cortex, Isatidis folium, Carthami*	Rehmannia glutinosa (Gaertn.) DC. [Orobanchaceae](Rehmanniae Radix Recens), Paeonia × suffruticosa Andrews [Paeoniaceae], Paeonia anomala subsp. veitchii (Lynch) D.Y.Hong & K.Y.Pan [Paeoniaceae], Angelica sinensis (Oliv.) Diels [Apiaceae], Sophora	Granule	N	N

			flos, Saposhnikoviae radix	flavescens Aiton [Fabaceae], Lonicera japonica Thunb. [Caprifoliaceae], Scrophularia ningpoensis Hemsl. [Scrophulariaceae], Arctium lappa L. [Asteraceae], Cryptotympana dubia (Haupt) [Cicadidae], Dictamnus dasycarpus Turcz. [Rutaceae], Isatis tinctoria subsp. tinctoria [Brassicaceae], Carthamus tinctorius L. [Asteraceae], Saposhnikovia divaricata (Turcz. ex Ledeb.) Schischk. [Apiaceae]			
Zhou (2018)	Keyin pills	Commercial supplier Jilin	*Smilax glabrae rhizoma, Menispermum*	Smilax glabra Roxb. [Smilacaceae], Menispermum	Pill	N	N

		Tiantai Pharmaceutical Co., Ltd.	*dauricum, Dictamni radicis cortex, Polygonum bistorta*	dauricum DC. [Menispermaceae], Dictamnus dasycarpus Turcz. [Rutaceae], Bistorta officinalis Delarbre [Polygonaceae]			
Chen (2019)	Compound glycyrrhizin	Commercial supplier Lepu Pharmaceutical Co., Ltd.	*Glycyrrhizae radix et rhizoma*	Glycyrrhiza uralensis Fisch. ex DC. [Fabaceae]	Tablet	N	N
Ge (2019)	Compound glycyrrhizin	Commercial supplier Ruiyang Pharmaceutical Co., Ltd.	*Glycyrrhizae radix et rhizoma*	Glycyrrhiza uralensis Fisch. ex DC. [Fabaceae]	Capsule	N	N
Han (2019)	Qinzhuliangxue feng	Prepared by Researchers	*Scutellariae radix, Ecliptae herba, Rehmanniae radix recens, Scrophulariae radix, Saposhnikoviae radix,*	Scutellaria baicalensis Georgi [Lamiaceae], Eclipta prostrata (L.) L. [Asteraceae], Rehmannia glutinosa (Gaertn.) DC. [Orobanchaceae](Rehmanniae Radix	Decoction	N	N

	Lithospermi radix, Glycyrrhizae radix et rhizoma, Cynanchi paniculati radix et rhizoma, Paeoniae radix rubra, Moutan radicis cortex, Lonicerae flos, Margaritifera concha	Recens), Scrophularia ningpoensis Hemsl. [Scrophulariaceae], Saposhnikovia divaricata (Turcz. ex Ledeb.) Schischk. [Apiaceae], Arnebia euchroma (Royle ex Benth.) I.M.Johnst. [Boraginaceae], Glycyrrhiza uralensis Fisch. ex DC. [Fabaceae], Vincetoxicum mukdenense Kitag. [Apocynaceae], Paeonia anomala subsp. veitchii (Lynch) D.Y.Hong & K.Y.Pan [Paeoniaceae], Paeonia × suffruticosa Andrews [Paeoniaceae], Lonicera japonica Thunb. [Caprifoliaceae],	

				Pinctada imbricata Röding [Pteriidae]			
Hu (2019)	Liangxuexiaobi decoction	Prepared by Researchers	*Rehmanniae radix recens, Rehmanniae radix preparata, Bubalus bubalis, Sophorae flos, Scrophulariae radix, Duchesnea indica, Scutellariae barbatae herba, Moutan radicis cortex, Paeoniae radix rubra, Imperatae rhizoma, Hedyotidis herba*	Rehmannia glutinosa (Gaertn.) DC. [Orobanchaceae](Rehmanniae Radix Recens), Rehmannia glutinosa (Gaertn.) DC. [Orobanchaceae](Rehmanniae Radix Preparata), Bubalus bubalis Linné [Bovidae], Styphnolobium japonicum (L.) Schott [Fabaceae], Scrophularia ningpoensis Hemsl. [Scrophulariaceae], Duchesnea indica, Scutellaria barbata D.Don [Lamiaceae], Paeonia × suffruticosa Andrews [Paeoniaceae], Paeonia anomala	Decoction	N	N

				subsp. veitchii (Lynch) D.Y.Hong & K.Y.Pan [Paeoniaceae], Imperata cylindrica (L.) Raeusch. [Poaceae], Scleromitrion diffusum (Willd.) R.J.Wang [Rubiaceae]			
Lu (2019)	Yangxuequfeng granule	Commercial supplier Shanghai China Resources 999 Pharmaceutical Co., Ltd.	*Angelicae sinensis radix, Paeonia lactiflora, Cnidii rhizoma, Rehmanniae radix recens, Spatholobi caulis, Polygoni multiflori radix, Tribuli fructus, Saposhnikoviae radix, Schizonepetae spica, Glycyrrhizae radix et rhizoma*	Angelica sinensis (Oliv.) Diels [Apiaceae], Paeonia lactiflora Pall. [Paeoniaceae](Paeonia lactiflora Alba), Conioselinum anthriscoides 'Chuanxiong' [Apiaceae], Rehmannia glutinosa (Gaertn.) DC. [Orobanchaceae](Rehmanniae Radix Recens), Spatholobus suberectus Dunn [Fabaceae],	Granule	N	N

				Reynoutria multiflora (Thunb.) Moldenke [Polygonaceae], Tribulus terrestris L. [Zygophyllaceae], Saposhnikovia divaricata (Turcz. ex Ledeb.) Schischk. [Apiaceae], Nepeta tenuifolia Benth. [Lamiaceae], Glycyrrhiza uralensis Fisch. ex DC. [Fabaceae]			
Xun (2019)	Yinxie capsule	Commercial supplier Xinjiang Wei-A-Tang Pharmaceutical Co., Ltd.	*Smilax glabrae rhizoma, Smilacis chinae rhizoma*	Smilax glabra Roxb. [Smilacaceae], Smilax china L. [Smilacaceae]	Capsule	N	N
Yang (2019)	Compound Qingdai capsule	Commercial supplier Shaanxi Pharmaceutical	*Indigo pulverata levis, Mume fructus, Taraxaci herba, Lithospermi*	Persicaria tinctoria (Aiton) Spach [Polygonaceae], Prunus mume (Siebold) Siebold &	Capsule	N	N

	Holdings Group Tianning Pharmaceutical Co., LTD.	*radix, Angelicae dahuricae radix, Salviae miltiorrhizae radix, Dictamni radicis cortex, Massa medicata fermentata, Dryopteridis crassirhizomatis rhizoma, Smilax glabrae rhizoma, Portulacae herba, Dioscorea hypoglauca, Crataegi fructus, Schisandrae fructus*	Zucc. [Rosaceae], Taraxacum mongolicum Hand.-Mazz. [Asteraceae], Arnebia euchroma (Royle ex Benth.) I.M.Johnst. [Boraginaceae], Angelica dahurica (Hoffm.) Benth. & Hook.f. ex Franch. & Sav. [Apiaceae], Salvia miltiorrhiza Bunge [Lamiaceae], Dictamnus dasycarpus Turcz. [Rutaceae], Massa Medicata Fermentata, Dryopteris crassirhizoma Nakai [Polypodiaceae], Smilax glabra Roxb. [Smilacaceae], Portulaca oleracea L. [Portulacaceae], Dioscorea collettii var. hypoglauca	

				(Palib.) S.J.Pei & C.T.Ting [Dioscoreaceae], Crataegus pinnatifida var. pinnatifida [Rosaceae], Schisandra chinensis (Turcz.) Baill. [Schisandraceae]			
Yao (2019)	Liangxuerun zao decoction	Prepared by Researchers	*Smilax glabrae rhizoma, Lonicerae folium et caulis, Isatidis radix, Paris polyphylla, Dictamni radicis cortex, Rehmanniae radix recens, Clematidis radix, Anemarrhenae rhizoma, Sophorae tonkinensis radix et rhizoma, Glycyrrhizae radix et rhizoma*	Smilax glabra Roxb. [Smilacaceae], Lonicera japonica Thunb. [Caprifoliaceae], Isatis tinctoria subsp. tinctoria [Brassicaceae](Isatidis Radix), Paris polyphylla, Dictamnus dasycarpus Turcz. [Rutaceae], Rehmannia glutinosa (Gaertn.) DC. [Orobanchaceae](Rehmanniae Radix Recens), Clematis	Decoction	N	N

				chinensis Osbeck [Ranunculaceae], Anemarrhena asphodeloides Bunge [Asparagaceae], Sophora tonkinensis Gagnep. [Fabaceae], Glycyrrhiza uralensis Fisch. ex DC. [Fabaceae]			
Zhong (2019)	Piminxiao capsule	Commercial supplier Sichuan Defeng Pharmaceutical Co., Ltd.	*Sophorae radix, Atractylodis rhizoma, Saposhnikoviae radix, Schizonepetae spica, Tribuli fructus, Dictamni radicis cortex, Cnidi fructus, Xanthii fructus, Scolopendra, Indigo pulverata levis, Taraxaci herba, Violae herba, Scutellariae radix,*	Sophora flavescens Aiton [Fabaceae], Atractylodes lancea (Thunb.) DC. [Asteraceae](Atractylodis Rhizoma), Saposhnikovia divaricata (Turcz. ex Ledeb.) Schischk. [Apiaceae], Nepeta tenuifolia Benth. [Lamiaceae], Tribulus terrestris L. [Zygophyllaceae], Dictamnus dasycarpus Turcz. [Rutaceae], Cnidium	Capsule	N	N

	Phellodendri cortex, Coptis chinensis, Cicadidae periostracum, Rehmanniae radix recens, Moutan radicis cortex, Tamarics cacumen, Lithospermi radix, Lycii radicis cortex	monnieri (L.) Cusson [Apiaceae], Xanthium strumarium L. [Asteraceae], Scolopendra subspinipes mutilans Linné Koch [Scolopendridae], Persicaria tinctoria (Aiton) Spach [Polygonaceae], Taraxacum mongolicum Hand.-Mazz. [Asteraceae], Viola mandshurica W.Becker [Violaceae], Scutellaria baicalensis Georgi [Lamiaceae], Phellodendron chinense C.K.Schneid. [Rutaceae], Coptis chinensis Franch. [Ranunculaceae], Cryptotympana dubia (Haupt) [Cicadidae],	

				Rehmannia glutinosa (Gaertn.) DC. [Orobanchaceae](Rehmanniae Radix Recens), Paeonia × suffruticosa Andrews [Paeoniaceae], Tamarix chinensis Lour. [Tamaricaceae], Arnebia euchroma (Royle ex Benth.) I.M.Johnst. [Boraginaceae], Lycium barbarum L. [Solanaceae]			
Chen (2020)	Compound glycyrrhizin	Commercial supplier Ruiyang Pharmaceutical Co., Ltd.	*Glycyrrhizae radix et rhizoma*	Glycyrrhiza uralensis Fisch. ex DC. [Fabaceae]	Capsule	N	N
Hao (2020)	Liangxuexiaobi Pill	Prepared by Researchers	*Rehmanniae radix recens, Paeoniae radix rubra, Saposhnikoviae radix, Hedyotidis herba,*	Rehmannia glutinosa (Gaertn.) DC. [Orobanchaceae](Rehmanniae Radix Recens), Paeonia anomala subsp.	Pill	N	N

			Scutellariae rarbatae herba, Isatidis folium, Salviae miltiorrhizae radix, Astragali radix, Glycyrrhizae radix et rhizoma	veitchii (Lynch) D.Y.Hong & K.Y.Pan [Paeoniaceae], Saposhnikovia divaricata (Turcz. ex Ledeb.) Schischk. [Apiaceae], Scleromitrion diffusum (Willd.) R.J.Wang [Rubiaceae], Scutellaria barbata D.Don [Lamiaceae], Isatis tinctoria subsp. tinctoria [Brassicaceae], Salvia miltiorrhiza Bunge [Lamiaceae], Astragalus mongholicus Bunge [Fabaceae], Glycyrrhiza uralensis Fisch. ex DC. [Fabaceae]			
Ji (2020)	Compound Qingdai capsule	Commercial supplier Shaanxi	*Indigo pulverata levis, Mume fructus, Taraxaci*	Persicaria tinctoria (Aiton) Spach [Polygonaceae],	Capsule	N	N

	Pharmaceutical Holdings Group Tianning Pharmaceutical Co., LTD.	*herba, Lithospermi radix, Angelicae dahuricae radix, Salviae miltiorrhizae radix, Dictamni radicis cortex, Massa medicata fermentata, Dryopteridis crassirhizomatis rhizoma, Smilax glabrae rhizoma, Portulacae herba, Dioscorea hypoglauca, Crataegi fructus, Schisandrae fructus*	Prunus mume (Siebold) Siebold & Zucc. [Rosaceae], Taraxacum mongolicum Hand.-Mazz. [Asteraceae], Arnebia euchroma (Royle ex Benth.) I.M.Johnst. [Boraginaceae], Angelica dahurica (Hoffm.) Benth. & Hook.f. ex Franch. & Sav. [Apiaceae], Salvia miltiorrhiza Bunge [Lamiaceae], Dictamnus dasycarpus Turcz. [Rutaceae], Massa Medicata Fermentata, Dryopteris crassirhizoma Nakai [Polypodiaceae], Smilax glabra Roxb. [Smilacaceae], Portulaca oleracea L. [Portulacaceae],

				Dioscorea collettii var. hypoglauca (Palib.) S.J.Pei & C.T.Ting [Dioscoreaceae], Crataegus pinnatifida var. pinnatifida [Rosaceae], Schisandra chinensis (Turcz.) Baill. [Schisandraceae]			
Liu (2020a)	Compound glycyrrhizin	Commercial supplier Eisai Pharmaceutical Co., Ltd.	*Glycyrrhizae radix et rhizoma*	Glycyrrhiza uralensis Fisch. ex DC. [Fabaceae]	Tablet	N	N\
Liu (2020b)	Xiaoyin decoction	Prepared by Researchers	*Spatholobi caulis, Salviae miltiorrhizae radix, Sophorae flos, Imperatae rhizoma, Rehmanniae radix recens , Rehmanniae radix preparata,*	Spatholobus suberectus Dunn [Fabaceae], Salvia miltiorrhiza Bunge [Lamiaceae], Styphnolobium japonicum (L.) Schott [Fabaceae], Imperata cylindrica (L.) Raeusch. [Poaceae],	Decoction	N	N

			Angelicae sinensis radix, Moutan radicis cortex, Lithospermi radix, Glycyrrhizae radix et rhizoma	Rehmannia glutinosa (Gaertn.) DC. [Orobanchaceae](Rehmanniae Radix Recens), Rehmannia glutinosa (Gaertn.) DC. [Orobanchaceae](Rehmanniae Radix Preparata), Angelica sinensis (Oliv.) Diels [Apiaceae], Paeonia × suffruticosa Andrews [Paeoniaceae], Arnebia euchroma (Royle ex Benth.) I.M.Johnst. [Boraginaceae], Glycyrrhiza uralensis Fisch. ex DC. [Fabaceae]			
Lu (2020)	Total Glycosides of Paeoniae Alba capsule	Commercial supplier Ningbo Lihua Pharmaceutical Co.,	*Paeonia lactiflora*	Paeonia lactiflora Pall. [Paeoniaceae](Paeonia lactiflora Alba)	Capsule	N	N

		Ltd.					
Qu (2020)	Yinxie capsule	Commercial supplier Shaanxi Dongtai Pharmaceutical Co., Ltd.	*Smilax glabrae rhizoma, Smilacis chinae rhizoma*	Smilax glabra Roxb. [Smilacaceae], Smilax china L. [Smilacaceae]	Capsule	N	N
Ren (2020)	Xiaoyin granule	Prepared by Researchers	*Sophorae radix, Angelicae sinensis radix, Rehmanniae radix recens, Scrophulariae radix, Moutan radicis cortex, Paeoniae radix rubra, Isatidis folium, Carthami flos, Gardenia jasminoides*	Sophora flavescens Aiton [Fabaceae], Angelica sinensis (Oliv.) Diels [Apiaceae], Rehmannia glutinosa (Gaertn.) DC. [Orobanchaceae](Rehmanniae Radix Recens), Scrophularia ningpoensis Hemsl. [Scrophulariaceae], Paeonia × suffruticosa Andrews [Paeoniaceae], Paeonia anomala subsp. veitchii (Lynch) D.Y.Hong & K.Y.Pan	Granule	N	N

				[Paeoniaceae], Isatis tinctoria subsp. tinctoria [Brassicaceae], Carthamus tinctorius L. [Asteraceae], Gardenia jasminoides J.Ellis [Rubiaceae]			
Shen (2020)	Liangxuejiedu decoction	Prepared by Researchers	*Smilax glabrae rhizoma, Isatidis folium, Lonicerae flos, Rehmanniae radix recens , Paeoniae radix rubra, Paeonia lactiflora, Cnidii rhizoma, Angelicae sinensis radix, Thujae orientalis folium, Moutan radicis cortex, Lithospermi radix, Sophorae flos, Glycyrrhizae radix et rhizoma*	Smilax glabra Roxb. [Smilacaceae], Isatis tinctoria subsp. tinctoria [Brassicaceae], Lonicera japonica Thunb. [Caprifoliaceae], Rehmannia glutinosa (Gaertn.) DC. [Orobanchaceae](Rehmanniae Radix Recens) , Paeonia anomala subsp. veitchii (Lynch) D.Y.Hong & K.Y.Pan [Paeoniaceae], Paeonia lactiflora Pall.	Decoction	N	N

				[Paeoniaceae](Paeonia lactiflora Alba), Conioselinum anthriscoides ‘Chuanxiong’ [Apiaceae], Angelica sinensis (Oliv.) Diels [Apiaceae], Thujae Orientalis Folium, Paeonia × suffruticosa Andrews [Paeoniaceae], Arnebia euchroma (Royle ex Benth.) I.M.Johnst. [Boraginaceae], Styphnolobium japonicum (L.) Schott [Fabaceae], Glycyrrhiza uralensis Fisch. ex DC. [Fabaceae]			
Wu (2020)	Compound glycyrrhizin	Commercial supplier Lepu Pharmaceutical Co.,	*Glycyrrhizae radix et rhizoma*	Glycyrrhiza uralensis Fisch. ex DC. [Fabaceae]	Tablet	N	N

		Ltd.					
Yang (2020)	Xiaoyin granule	Commercial supplier Shaanxi Kanghui Pharmaceutical Co., Ltd.	*Rehmanniae radix recens, Moutan radicis cortex, Paeoniae radix rubra, Angelicae sinensis radix, Sophorae radix, Lonicerae flos, Scrophulariae radix, Arctii fructus, Cicadidae periostracum, Dictamni radicis cortex, Isatidis folium, Carthami flos, Saposhnikoviae radix*	Rehmannia glutinosa (Gaertn.) DC. [Orobanchaceae](Rehmanniae Radix Recens), Paeonia × suffruticosa Andrews [Paeoniaceae], Paeonia anomala subsp. veitchii (Lynch) D.Y.Hong & K.Y.Pan [Paeoniaceae], Angelica sinensis (Oliv.) Diels [Apiaceae], Sophora flavescens Aiton [Fabaceae], Lonicera japonica Thunb. [Caprifoliaceae], Scrophularia ningpoensis Hemsl. [Scrophulariaceae], Arctium lappa L. [Asteraceae], Cryptotympana dubia (Haupt)	Granule	N	N

				[Cicadidae], Dictamnus dasycarpus Turcz. [Rutaceae], Isatis tinctoria subsp. tinctoria [Brassicaceae], Carthamus tinctorius L. [Asteraceae], Saposhnikovia divaricata (Turcz. ex Ledeb.) Schischk. [Apiaceae]			
Zheng (2020)	Jueyin granule	Commercial supplier Shenzhen China Resources Sanjiu Modern Chinese Medicine Co., Ltd.	*Lonicerae flos, Concha haliotidis, Moutan radicis cortex, Rehmanniae radix recens, Isatidis folium, Hedyotidis herba, Curcumae radix*	Lonicera japonica Thunb. [Caprifoliaceae], Concha haliotidis, Paeonia × suffruticosa Andrews [Paeoniaceae], Rehmannia glutinosa (Gaertn.) DC. [Orobanchaceae](Rehmanniae Radix Recens), Isatis tinctoria subsp. tinctoria	Granule	N	N

				[Brassicaceae], Scleromitrion diffusum (Willd.) R.J.Wang [Rubiaceae], Curcuma phaeocaulis Valeton [Zingiberaceae]			
Jin (2021)	Xiaoyin decoction	Prepared by Researchers	*Rehmanniae radix recens, Lithospermi radix, Scutellariae radix, Lonicerae flos, Smilax glabrae rhizoma, Sanguisorbae radix, Hedyotidis herba, Persicae semen, Zaocys, Hirudo*	Rehmannia glutinosa (Gaertn.) DC. [Orobanchaceae](Rehmanniae Radix Recens), Arnebia euchroma (Royle ex Benth.) I.M.Johnst. [Boraginaceae], Scutellaria baicalensis Georgi [Lamiaceae], Lonicera japonica Thunb. [Caprifoliaceae], Smilax glabra Roxb. [Smilacaceae], Sanguisorbae Radix, Scleromitrion diffusum (Willd.) R.J.Wang	Decoction	N	N

				[Rubiaceae], Prunus persica (L.) Batsch [Rosaceae], Zaocys dhumnades Cantor [Colubridae], Hirudo niponica Whitman [Hirudinidae]			
Lan (2021)	Dangguiyinzi	Prepared by Researchers	*Angelicae sinensis radix, Paeonia lactiflora, Lithospermi radix, Rehmanniae radix recens, Saposhnikoviae radix, Schizonepetae spica, Tribuli fructus, Polygoni multiflori radix, Asparagi tuber, Liriopis seu ophiopogonis tuber, Salviae miltiorrhizae radix, Sophorae radix,*	Angelica sinensis (Oliv.) Diels [Apiaceae], Paeonia lactiflora Pall. [Paeoniaceae](Paeonia lactiflora Alba), Arnebia euchroma (Royle ex Benth.) I.M.Johnst. [Boraginaceae], Rehmannia glutinosa (Gaertn.) DC. [Orobanchaceae](Rehmanniae Radix Recens), Saposhnikovia divaricata (Turcz. ex Ledeb.) Schischk. [Apiaceae], Nepeta tenuifolia Benth.	Decoction	N	N

			Glycyrrhizae radix et rhizoma	[Lamiaceae], Tribulus terrestris L. [Zygophyllaceae], Reynoutria multiflora (Thunb.) Moldenke [Polygonaceae], Asparagus cochinchinensis (Lour.) Merr. [Asparagaceae], Ophiopogon japonicus (Thunb.) Ker Gawl. [Asparagaceae], Salvia miltiorrhiza Bunge [Lamiaceae], Sophora flavescens Aiton [Fabaceae], Glycyrrhiza uralensis Fisch. ex DC. [Fabaceae]			
Le (2021)	Tianxian decoction	Prepared by Researchers	*Smilacis chinae rhizoma, Rehmanniae radix recens, Trichosanthes kirilowii,*	Smilax china L. [Smilacaceae], Rehmannia glutinosa (Gaertn.) DC. [Orobanchaceae](Rehmanniae Radix	Granule	N	N

	Anemarrhenae rhizoma, Clerodendrum cyrtophyllum, Phyllostachys nigra, Paeoniae radix rubra , Dictamni radicis cortex, Saposhnikoviae radix, Tripterygium wilfordii, Hedyotidis herba, Glycyrrhizae radix et rhizoma	Recens), Trichosanthes kirilowii Maxim. [Cucurbitaceae], Anemarrhena asphodeloides Bunge [Asparagaceae], Clerodendrum cyrtophyllum Turcz. [Lamiaceae] , Phyllostachys nigra var. henonis (Mitford) Rendle [Poaceae], Paeonia anomala subsp. veitchii (Lynch) D.Y.Hong & K.Y.Pan [Paeoniaceae] , Dictamnus dasycarpus Turcz. [Rutaceae], Saposhnikovia divaricata (Turcz. ex Ledeb.) Schischk. [Apiaceae], Tripterygium wilfordii Hook.f.	

				[Celastraceae], Scleromitrion diffusum (Willd.) R.J.Wang [Rubiaceae], Glycyrrhiza uralensis Fisch. ex DC. [Fabaceae]			
Tang (2021)	Qingreyangxuejiedu decoction	Prepared by Researchers	*Rehmanniae radix recens, Trichosanthes kirilowii, Angelicae sinensis radix, Salviae miltiorrhizae radix, Paeonia lactiflora, Paeoniae radix rubra, Spatholobi caulis, Dictamni radicis cortex, Smilax glabrae rhizoma, Lycii radicis cortex*	Rehmannia glutinosa (Gaertn.) DC. [Orobanchaceae](Rehmanniae Radix Recens), Trichosanthes kirilowii Maxim. [Cucurbitaceae], Angelica sinensis (Oliv.) Diels [Apiaceae], Salvia miltiorrhiza Bunge [Lamiaceae], Paeonia lactiflora Pall. [Paeoniaceae](Paeonia lactiflora Alba), Paeonia anomala subsp. veitchii (Lynch) D.Y.Hong &	Decoction	N	N

				K.Y.Pan [Paeoniaceae], Spatholobus suberectus Dunn [Fabaceae], Dictamnus dasycarpus Turcz. [Rutaceae], Smilax glabra Roxb. [Smilacaceae], Lycium barbarum L. [Solanaceae]			
Wang (2021)	Qingreliangxue decoction	Prepared by Researchers	*Astragali Radix, Poria sclerotium, Atractylodis rhizoma alba, Rehmanniae radix recens, Moutan radicis cortex, Paeoniae radix r0ubra, Kochiae fructus, Dictamni radicis cortex, Isatidis radix, Isatidis folium, Rubiae radix, Prunellae spica,*	Astragalus mongholicus Bunge [Fabaceae, Poria cocos Wolf [Polyporaceae], Atractylodes lancea (Thunb.) DC. [Asteraceae], Rehmannia glutinosa (Gaertn.) DC. [Orobanchaceae](Rehmanniae Radix Recens), Paeonia × suffruticosa Andrews [Paeoniaceae],	Decoction	N	N

	Curcumae radix, Polygoni multiflori radix, Ecliptae herba	Paeonia anomala subsp. veitchii (Lynch) D.Y.Hong & K.Y.Pan [Paeoniaceae], Bassia scoparia (L.) A.J.Scott [Amaranthaceae], Dictamnus dasycarpus Turcz. [Rutaceae], Isatis tinctoria subsp. tinctoria [Brassicaceae](Isatidis Radix), Isatis tinctoria subsp. tinctoria [Brassicaceae], Rubia tinctorum L. [Rubiaceae], Prunella vulgaris L. [Lamiaceae], Curcuma phaeocaulis Valeton [Zingiberaceae], Reynoutria multiflora (Thunb.) Moldenke [Polygonaceae], Eclipta prostrata (L.)	

L. [Asteraceae]

Latin names of herbs are based on the standard nomenclature of the Korean Pharmacopoeia (https://nifds.go.kr/nhmi/main.do); scientific names of herbs are based on the Medicinal Plant Names Services (MPNS, https://mpns.science.kew.org/mpns-portal/?_ga=1.111763972.1427522246.1459077346) provided by the Royal Botanic Gardens, Kew.
